# Online Media Use and COVID-19 Vaccination in Real-World Personal Networks: Quantitative Study

**DOI:** 10.2196/58257

**Published:** 2024-10-25

**Authors:** Iulian Oană, Marian-Gabriel Hâncean, Matjaž Perc, Jürgen Lerner, Bianca-Elena Mihăilă, Marius Geantă, José Luis Molina, Isabela Tincă, Carolina Espina

**Affiliations:** 1 Department of Sociology University of Bucharest Bucharest Romania; 2 Center for Innovation in Medicine Bucharest Romania; 3 The Research Institute of the University of Bucharest University of Bucharest Bucharest Romania; 4 Faculty of Natural Sciences and Mathematics University of Maribor Maribor Slovenia; 5 Community Healthcare Center Dr. Adolf Drolc Maribor Maribor Slovenia; 6 Complexity Science Hub Vienna Vienna Austria; 7 Department of Physics Kyung Hee University Seoul Republic of Korea; 8 Department of Computer and Information Science University of Konstanz Konstanz Germany; 9 Research Group on Fundamental and Oriented Anthropology (GRAFO), Department of Social and Cultural Anthropology Universitat Autònoma de Barcelona Barcelona Spain; 10 Environment and Lifestyle Epidemiology Branch International Agency for Research on Cancer Lyon France

**Keywords:** vaccine hesitancy, online media, social media, assortative mixing, personal network analysis, social network analysis, Romania, vaccination, health information, COVID-19

## Abstract

**Background:**

Most studies assessing the impact of online media and social media use on COVID-19 vaccine hesitancy predominantly rely on survey data, which often fail to capture the clustering of health opinions and behaviors within real-world networks. In contrast, research using social network analysis aims to uncover the diverse communities and discourse themes related to vaccine support and hesitancy within social media platforms. Despite these advancements, there is a gap in the literature on how a person’s social circle affects vaccine acceptance, wherein an important part of social influence stems from offline interactions.

**Objective:**

We aimed to examine how online media consumption influences vaccination decisions within real-world social networks by analyzing unique quantitative network data collected from Romania, an Eastern European state and member of the European Union.

**Methods:**

We conducted 83 face-to-face interviews with participants from a living lab in Lerești, a small rural community in Romania, using a personal network analysis framework. This approach involved gathering data on both the respondents and individuals within their social circles (referred to as *alters*). After excluding cases with missing data, our analysis proceeded with 73% (61/83) of the complete personal networks. To examine the hierarchical structure of alters nested within ego networks, we used a mixed multilevel logistic regression model with random intercepts. The model aimed to predict vaccination status among alters, with the focal independent variable being the respondents’ preferred source of health and prevention information. This variable was categorized into 3 types: traditional media, online media (including social media), and a combination of both, with traditional media as the reference category.

**Results:**

In this study, we analyzed 61 personal networks, encompassing between 15 and 25 alters each, totaling 1280 alters with valid data across all variables of interest. Our primary findings indicate that alters within personal networks, whose respondents rely solely on online media for health information, exhibit lower vaccination rates (odds ratio [OR] 0.37, 95% CI 0.15-0.92; *P*=.03). Conversely, the transition from exclusive traditional media use to a combination of both traditional and online media does not significantly impact vaccination rate odds (OR 0.75, 95% CI 0.32-1.78; *P*=.52). In addition, our analysis revealed that alters in personal networks of respondents who received the vaccine are more likely to have received the vaccine themselves (OR 3.75, 95% CI 1.79-7.85; *P*<.001).

**Conclusions:**

Real-world networks combine diverse human interactions and attributes along with consequences on health opinions and behaviors. As individuals’ vaccination status is influenced by how their social alters use online media and vaccination behavior, further insights are needed to create tailored communication campaigns and interventions regarding vaccination in areas with low levels of digital health literacy and vaccination rates, as Romania exposes.

## Introduction

### Background

Currently, COVID-19 is not as prominent on the public agenda despite the fact that infections with SARS-CoV-2 are still occurring. At the 76th World Health Assembly, held between May 21 and May 30, 2023, in Geneva, the chief of the World Health Organization (WHO) Tedros Adhanom Ghebreyesus stated that the threat of other pandemics, either related to mutations of SARS‑CoV‑2 or other pathogens, is still present [1]. Moreover, all the debates regarding vaccination during the COVID-19 pandemic affected how people approach other vaccines, such as routine childhood vaccines [2] and raised questions about the impact of human papillomavirus and hepatitis B virus vaccination [3]. Vaccine hesitancy has been a significant threat to public health, especially in high-income nations [4]. The Strategic Advisory Group of Experts on Immunization (SAGE) of the WHO defined vaccine hesitancy as the “delay in acceptance or refusal of vaccination despite the availability of vaccination services” [5]. The SAGE working group adds that vaccination hesitancy is context-specific, a complex of beliefs and behaviors conditional on culture, history, and vaccine type.

While vaccine hesitancy is not a new phenomenon [6,7], the landscape in which it exists today is new. With the advent of the internet, social media, and people interconnected in a global network, which further scales up the phenomenon’s complexity, the WHO has stressed the importance of managing *infodemics* for efficient interventions against the COVID-19 pandemic [8]. The WHO defines an *infodemic* as “too much information, including false or misleading information in digital and physical environments during a disease outbreak” [9]. Working by misinformation or disinformation can lead to “confusion and risk-taking behaviors that can harm health. It also leads to mistrust in health authorities and undermines the public health response” [9]. Compared to traditional media (eg, official news channels, radio, or newspapers), online media, in general, and social media, in particular, facilitate the spread of unverified medical information [10,11] and the creation of echo chambers where false information, conspiracy theories, and fears are reinforced [12].

Evidence on how online media and social media use affects COVID-19 vaccination is mixed. For instance, extant research indicates that these media types are either positively [13-19] or negatively correlated with vaccine hesitancy [20,21]. However, most studies presented in systematic reviews [22,23] or systematic reviews of reviews [24] suggest that online and social media use tends to increase vaccination hesitancy. Similar conclusions were also drawn from analyses of cross-national surveys [13,19]. For studies that found a correlation between social media use and vaccination acceptance, it is generally observed that such results typically emerge from specific samples, such as adolescents [25], who are more inclined to seek information online or within unique cultural contexts, such as countries like China [21], where the interaction with social media content significantly differs [26].

Supplementary results are also provided by scholars who analyzed the effects of social media in comparison with traditional media. Some studies have found that the use of legacy media has a positive effect on vaccine acceptance, alongside the negative effect associated with social media [14,15,18]. In other studies, the focus was primarily on the correlation with traditional media, revealing that the use of social media had no significant effect [27,28]. Interestingly, a study found that not even traditional media as a whole category can correlate with vaccine acceptance. Viswanath et al [20] distinguished between the use of mainstream broadcast media and mainstream print media and found that only the latter positively affected the acceptance of the COVID-19 vaccine, the former being nonsignificant. In other research, no direct association was found between online media or social media use and vaccine hesitancy, or it was observed that the effects of social media vary due to indirect influences. Specifically, it was demonstrated that social media consumption can positively impact vaccine hesitancy when mediated by confidence in the vaccine’s safety [27]. Lee and You [29] also emphasized the importance of indirect associations. Their study found that even if social media, in general, has a positive impact on vaccine hesitancy, this effect can be amplified by the respondents’ perceived risk of vaccine-induced side effects or reversed through the respondents’ perceived risk of being infected with SARS-CoV-2.

Given that most data on vaccination hesitancy during the COVID-19 pandemic were collected through standard surveys, statistical analyses have been conducted to assess the effects of media use on vaccine hesitancy. These analyses control for various sociodemographic traits (such as gender, income, education, and ethnic group) and other individual-level characteristics, including trust in government and health experts and vaccine confidence. Nevertheless, it is crucial to recognize that information dissemination is not a one-way process from the media outlets to isolated individuals. People’s opinions and attitudes are significantly shaped by their social circles, including family, peers, close friends, and other social groups they trust and interact with frequently. Opinions and behaviors regarding COVID-19 vaccination tend to be clustered within social groups rather than distributed randomly, as evidenced by studies conducted by Klaus et al [30] and Hâncean et al [31]. This observation underscores the necessity of considering real-world social clusters when examining the impact of media use on vaccination hesitancy. In line with this, the SAGE Working Group on Vaccine Hesitancy has acknowledged the significant role of social influence within the matrix of determinants for vaccine hesitancy [5].

Complementing studies that rely solely on standard survey data and focus on the attributes of individuals, network science introduces a more comprehensive approach by integrating attributes with relationships between units of analysis. Within this field, research on social actors falls into social network analysis (SNA) and personal network analysis (PNA), both of which conceptualize social actors as nodes and their relationships as ties. SNA concentrates on specific relationships within a defined population of nodes (eg, students within a school or users reposting a particular hashtag) in a bounded context, adopting a *sociocentric* design that encapsulates all nodes within a singular network. In contrast, PNA explores the wider social circles of individuals through an *egocentric* design, focusing on the networks that revolve around central nodes (or *egos*) and their connections to various social *alters*. These alters may not be part of the same group boundaries, leading to multiple, distinct networks that often cannot be aggregated into a single network [32].

Studies using SNA to examine COVID-19 vaccine hesitancy predominantly use datasets that capture user interactions within social media platforms. These studies typically concentrate on analyzing the content of discussions related to vaccine hesitancy and identifying key participants or groups within these conversations through community detection algorithms. This approach allows a nuanced understanding of the discourse dynamics and the social structures influencing vaccine hesitancy among online communities. Scholars found that while positive and negative discourses surrounding COVID-19 vaccination are present on social media platforms [26,33-36], they depend on political partisanship and the quality of sources [12,35]. Such studies are of great value for content analysis and mapping different communities inside online spaces. They identify online behavior patterns that, in the end, may affect day-to-day health decisions and give insights into how to create better communication campaigns for vaccine acceptance. Furthermore, they add nuance to the discussion of the relationship between the use of online platforms and vaccine hesitancy. Exposure to different sources in these online spaces (whom people choose to follow), the tendency to comment on posts presenting similar views [37], as well as prior biases (eg, general level of distrust [28], trust in government [29], or political partisanship [20]), stress the idea that health outcomes are contextual and contingent on how people engage with these online spaces and on what they bring to these spaces from offline influences.

However, research on the impact of real-world networks on COVID-19 vaccine hesitancy or acceptance [31] as well as on the extent to which these networks reflect online stances toward COVID-19 vaccination remains limited. This gap is significant given the potential for network structures and compositions to influence the transmission dynamics of SARS-CoV-2 [38-40]. Past studies have demonstrated that health outcomes are associated with *assortative mixing*. In many instances, assortative mixing, wherein nodes with similar traits form connections more frequently, serves as an indication of a network’s structure and composition, emphasizing the tendency for similar individuals to be more closely interconnected. Drivers of assortative mixing can be represented by *social influence* (or *contagion*), where behaviors or traits spread through the network; *social* *selection* (*homophily*), where individuals form connections based on similar characteristics; or *social context* (*confounding*), where external factors related to the environment or setting influence network formation by limiting with whom a node can create a tie [41,42]. Social contagion seeks to explain how nodal characteristics (behaviors, opinions, and other traits) change as a function of a node’s relations inside the network [43]. Homophily principles state that nodes tend to create ties with others who are similar to them [44]. Contextual influences refer to effects brought by macrolevel changes (or the lack of macrolevel changes) in the social environment that influence individual or group behavior [41]. As examples of health outcomes related to the aforementioned mechanisms, we can name obesity [45] or the adoption of weight loss behaviors [46], the adoption of clean cooking methods [47], and how physicians adopt treatment plans and screening practices [48,49]. Regarding vaccination, previous studies have shown that network assortativity has a positive correlation with influenza vaccinations [50] and opinions about COVID-19 vaccination [31] and that network structures can facilitate, in general, one’s acceptance or hesitancy through the influence of neighboring nodes [51].

Previous research has shown that a person’s social environment can influence their health behavior, including COVID-19 vaccine acceptance. Some studies relied on measures of individual social capital to investigate vaccine acceptance. Various methods to account for someone’s individual social capital include measures of interpersonal trust (social cohesion), civic participation (being a member of various organizations or clubs), and reciprocity (giving and receiving social support) [52-56]. The scholars using this approach found that higher levels of individual social capital are positively correlated with COVID-19 vaccine acceptance. Specifically, they found that persons with a higher individual social capital had a higher chance of being vaccinated or were willing to receive a booster shot [52], had a higher chance of wanting to receive vaccine [53], had a higher trust in COVID-19 vaccines [56], were more likely to volunteer for COVID-19 vaccine clinical trials [55], or received lower scores on various indices of vaccine hesitance [54,56]. Other studies investigated the relationship between vaccine hesitancy and one’s social circle by using the concept of *homophily* through diverse measures accounting for the association between respondents’ vaccination acceptance and that of their peers. They found that someone’s declared vaccination status is associated with the overall perceived uptake among their family and friends [57] or that the number of doses received by a person is positively correlated with the perceived number of doses among their social contacts [58]. Using experimental conditions, Leonhardt and Pezzuti [59] found that participants were more willing to receive vaccine when presented with cases of persons afflicted by COVID-19 who shared similar personality, behavioral, or ideological dimensions. Complementarily, Campbell et al [60] found that in the prospect of seeking a romantic partner, respondents’ vaccination status was associated with that of their hypothetical partner.

Qualitative studies supplement such findings, describing instances where one’s decision to receive vaccine against COVID-19 was influenced by seeing persons from their close social circle (family and friends) getting vaccinated [61] or hearing about their positive experience with the vaccine [62,63]. At the other end of the spectrum, scholars found through in-depth interviews that peer influences enacted in social groups can also act as a deterrent for vaccine acceptance [64].

The aforementioned approaches, which rely on standard surveys or qualitative interviews, bring important insights into the relationship between vaccine acceptance and peer influences. They support the general hypothesis referring to assortative mixing found in personal networks as a result of social contagion, social selection, or both. However, these studies use indicators that do not fully account for the features of a social circle. PNA brings the possibility of combining the compositional characteristics (eg, the proportion of contacts with a certain attribute) with structural features that are captured through alter-alter ties [32], for example, the density of a network indicating social cohesion or the fragmentation indicating the number of disconnected subgroups. Furthermore, it allows for expanding the assortativity measures from ego-alter ties (ie, the similarity between respondents and their contacts) to assortativity between alters on a given attribute. Using a PNA design, our study takes advantage of combining measures of structure and composition and analyzes them while controlling for their multilevel structure, with alters embedded in egos.

To investigate how consumption of online media influences vaccination decisions within real-world social networks, we used unique network data collected from Romania, a European Union (EU) member state situated in Eastern Europe. As a general context, Romania has the second lowest COVID-19 vaccination rate when compared to other EU countries. The latest data recorded by Our World in Data on June 10, 2022, showed that 41.27% of Romania’s population was fully vaccinated [65]. Such low vaccination rates are also troubling in the context of low digital literacy rates. Data from Eurostat show that in 2023 Romania ranked last among the EU states in terms the proportion of persons with basic or above basic overall digital skills, with 31.09% of Romanians compared with the EU average of 60.55% among individuals who used the internet in the last 3 months [66]. Among EU states, in 2023, Romania was fourth in internet use for social media platforms, with 84.35% of Romanians compared with the EU average of 64.74% for individuals who used the internet in the last 3 months [67]. Internet use for seeking health information is low but rapidly increasing. In 2022, Romania ranked last in this EU ranking, with 33.82% of Romanians compared with the EU average of 57.74% for individuals who used the internet in the last 3 months [67]. In 2023, Romania was placed third to last in the EU ranking of seeking health information online, with 51.71% of Romanians compared with the EU average of 61.51% for individuals who used the internet in the last 3 months [67].

Proxy indicators on Romanians’ digital health literacy show worrying facts about online media use. In 2023, the proportion of persons who checked the truthfulness of information from internet news sites or social media was the second lowest in the EU, with 11.24% of Romanians compared with the EU average of 26.38% for individuals who used the internet in the last 3 months [68]. The proportion of persons who checked the truthfulness of such information by checking the sources or comparing it with other information found on the internet further reinforces the ranking of Romania as second lowest in the EU, with 8.96% of Romanians compared with the EU average of 23.29% for individuals who used the internet in the last 3 months [68]. Eurostat figures about “evaluating data, information, and digital content” also indirectly show Romanians’ ability to identify misinformation or disinformation. The percentage of individuals reporting having seen untrue or doubtful information on the internet is the lowest in Romania, with 32.83% of Romanians compared with the EU average of 53.67% for individuals who used the internet in the last 3 months [68]. Such low numbers raise the question of whether Romanians truly do not encounter untrue information when using online media or cannot discern them from what is presented as accurate information.

### This Study

Our study focuses on the critical role of online media in influencing vaccination behaviors, using PNA on real-world social data. It specifically examines how the exclusive use of online media for health information correlates with the vaccination tendencies among individuals’ social contacts in rural Romania. The investigation uncovers that within the personal networks of those who rely solely on online sources, there is a discernible decrease in vaccination rates. This finding sheds light on the dynamics prevalent in regions marked by low COVID-19 vaccination uptake, limited digital health literacy, and overall digital literacy challenges.

Our work stands out for its application of unique quantitative data, offering a fresh perspective on the interaction between digital information consumption and health behavior, particularly in the context of vaccine hesitancy. Hopefully, we make a significant contribution by mixing online media use and personal network influences on vaccination decisions, thereby filling a crucial gap in the existing literature.

## Methods

### Ethical Considerations

The research was performed in accordance with the Declaration of Helsinki. The research protocol was approved by a named institutional and licensing committee. Specifically, the Center for Innovation in Medicine Ethics Committee reviewed and approved all the study procedures (EC-INOMED decision number D001/09-06-2023 and number D001/19-01-2024). All participants provided written informed consent. The privacy rights of the study participants were observed. The authors did not have access to information that could identify participants. After each interview, information that could identify the participants was anonymized. The study participants did not receive monetary compensation. Interviewees were rewarded for their participation with free access to a local educational program focused on health topics and a hotline number that they could use if they wanted expert opinions regarding medical problems or second opinions regarding a medical diagnostic.

### Data Collection

The data presented in this study were collected during the pilot phase of the 4P-CAN project (personalized cancer primary prevention research through citizen participation and digitally-enabled social innovation; HORIZON-MISS-2022-CANCER-01, project ID 101104432, program HORIZON) [69]. We collected data from one of 4P-CAN’s living lab in Lerești, a rural locality in Argeș county, Romania (N=4557).

The data collection process took place between September 13 and 30, 2023. We interviewed 83 persons following a PNA design [32]. PNA is a special type of network analysis; that is, the research design starts from nodes of interest, dubbed *egos*, and samples from their diverse social circles, composed of *alters*, through *name generators* (the questionnaire part wherein the respondents are asked to list their social contacts through various prompts that vary according to each research design in their formulation and number of elicited alters); *name interpreters* (the questionnaire sections wherein the egos give information about alters); and alter-alter ties (the questionnaire part where the respondents are asked to map various kinds of ties between elicited alters). In this manner, we can consider both the composition and structure of someone’s personal network.

Study participants were recruited using a respondent-driven link-tracing sampling methodology [70,71]. At the time of the interview, every interviewee was aged ≥18 years. Our study was carried out in accordance with appropriate recommendations, relevant guidelines, and regulations (specifically, those provided by the Romanian Sociologists Society, ie, the professional association of Romanian sociologists [72]).

Before data collection, the community was informed about the 4P-CAN project through social media posts on Facebook (Meta Platforms, Inc) and press conferences held by the local media. Furthermore, we had meetings with local authorities, citizens from the community, and key actors (eg, teachers, community leaders, and local physicians).

According to our data collection strategy, first, we aimed to avoid a low response rate due to the length of the questionnaire. Second, we intended to delineate a panel of respondents for future data collection. Given these reasons, we preferred a network-oriented sampling method to a probabilistic nonnetwork strategy, using respondent-driven link-tracing sampling [70,71]. Specifically, we started with 6 individuals known as *seeds*. These persons were selected based on ethnographic fieldwork preceding the data collection process. We sought to have seeds who were different in their characteristics. The sociodemographic profile of the 6 seeds differed by *sex* (n=4, 67% male and n=2, 33% female), *age* (n=1, 17% 34 years old; n=1, 17% 50 years old; n=1, 17% 52 years old; n=1, 17% 55 years old; and n=2, 33% 64 years old), *education* (n=5, 83% with university degrees and n=1, 17% with high school diploma), *personal income* (n=3, 50% below the average net salary and n=3, 50% above the average net salary), and *employment sector* (n=3, 50% employed in the public sector; n=1, 17% employed in the private sector; n=1, 17% self-employed; and n=1, 17% retired). After responding to our questionnaire, we asked the seeds to recommend other persons who might be interested in participating in our study. Then, we asked the recommended persons (who agreed to participate) to further nominate other persons, and so on. This created referee-referral chains inside the link-tracing network. Of the 6 seeds, 2 (33%) refused to participate but still made recommendations. In the end, the response rate was 54.2% (83/153). We also report a selection bias, which was induced by the tendency of participants to recommend others who are similar to them [70,71]. Related to the attribute of being vaccinated against COVID-19, we analyzed 87 dyads in the link-tracing network, for which we had information on COVID-19 vaccination for both referees and referrals (ie, both were study participants). In 76% (66/87) of cases, vaccinated participants referred those who were also vaccinated. In the rest of the 24% (21/87) of dyads, we observed disassortative recommendations, with 20 (23%) dyads representing cases where vaccinated persons recommended unvaccinated individuals and 1 (1%) case with the reverse.

To keep complete transparency about the research activities and to facilitate citizens’ engagement, before each interview, we discussed with the participants about the 4P-CAN project. Each participant received a paper dossier with informative materials about the project, including a consent form and a General Data Protection Regulation form, which they read and freely signed. They were also informed that they could opt out at any moment, that all data would be anonymized, and that they could consult the project’s web page, with a dedicated section (in Romanian) containing details about the methodology [69]. The participants were provided the contact details (phone numbers and email addresses) of the project director and research team in case they had or have future questions.

Each participant responded to a questionnaire that collected information about their opinions, behaviors, and characteristics of individuals from their social circle. Each interviewee was asked to nominate up to 25 people with whom they interacted frequently (face-to-face or via other communication methods) and who were aged ≥18 years. The prompt that we used was as follows: “Please nominate 25 people (18 years old plus) you interact with (or meet). You can start with the people you interact with most often. These may be family members, friends, acquaintances, neighbors, work colleagues, etc.” We did not collect separate data about face-to-face interactions and interactions via other communication methods. A respondent (ego) can consider that they have frequent interaction with an alter if they meet monthly but, on the other hand, communicate almost daily via calls or SMS text messages. The nominated persons could be *family members*, *partners,* *close friends, casual friends*, or *acquaintances.* Afterward, the respondents were asked about certain characteristics of the people they nominated in their social circle. All interviews were conducted face to face, having an average duration of 80 (SD 27.7) minutes. The questionnaires were applied using Network Canvas (Complex Data Collective) [73], a PNA research software. Network analysis, in general, distinguishes between 2 data types: attribute and relational. Attribute data refer to characteristics measured individually for each person (*node*) within the network, whereas relational data describe properties of the relations between the nodes, also known as ties [74]. In the context of PNA research, the nodes are the egos and alters, and ties refer to ego-alter and alter-alter relations. For each study participant (*ego*), we collected attribute data about them (eg, age, marital status, various health behaviors, and opinions), attribute data about persons in their social circle (*alters*), relational data capturing ego-alter relationships, and relational data about alter-alter ties. It is important to note that all aforementioned data types were captured from the perspective of the egos.

### Attribute Data

Of the attribute data, for both egos and alters, we collected information on their *sex* (1=female participant and 0=male participant), *age in years* (≥18 y), *being single* (1=yes and 0=no), and *COVID-19 vaccination status* (0=unvaccinated and 1=vaccinated). The education of egos and alters was measured by an ordinal scale capturing the last education level, with the values of 1=*no school*, 2=*less than primary school*, 3=*primary school*, 4=*less than secondary school*, 5=*secondary school*, 6=*arts and crafts school*, 7=*10 obligatory years*, 8=*high school (unfinished)*, 9=*high school (finished with diploma)*, 10=*posthigh school (nontertiary)*, 11=*bachelor’s degree or equivalent level*, 12=*master’s degree or equivalent level*, and 13=*Doctor of Philosophy or equivalent level*. *Employment status* (1=employed and 0=other) was measured only for egos. Additional information regarding egos’ or alters’ motives for getting vaccinated or their opinions about the COVID-19 vaccine or vaccination campaign were not collected.

We asked the egos (respondents) to provide details about their information sources related to medical topics. The egos had to respond to this question: “What are your sources of information about health and prevention (lifestyle)?” The respondents could select multiple answers from (1) “central television stations”; (2) “local press”; (3) “online, using Google (Google LLC), Bing (Microsoft Corp), or other search engines”; (4) “online, using social media (eg, Facebook, TikTok, and Instagram, etc)”; and (5) “online influencers.” In our analyses, this was introduced as a factor variable distinguishing between those who used *only traditional media* (options *1, 2,* or both), those who used *only online media* (*3*, *4,* *5,* or any combination of the 3 options), and those who used *both types of media*. Reliance on traditional media only was used as a reference level. While we acknowledge a difference between reliance on social media for news and information and other internet media, we chose to group the egos in the general category of *online media* because only 1 (1%) of the 83 initial respondents stated that they use only social media for information about health and prevention. Others who mentioned social media also mentioned search engines (and grouped as *online media* users) or legacy media (and grouped as *both media* users).

### Network Data

Regarding ego-alter relations, we considered the *emotional closeness* between egos and alters (“How emotionally close do you feel to this person?” with answer options “not at all close,” “not very close,” “close,” and “very close”) and their *direct interaction frequency* (“How often do you typically meet with this person?” with answer options “less than once a year,” “once a year,” “a few times a year,” “once a month,” “every 2 weeks,” “weekly,” and “daily”). This question took into account only face-to-face meetings and did not include interactions via other media (eg, phone calls or instant messaging through various platforms). In both cases, the variables were binarized. For emotional closeness, 1 represents situations where the ego said they have a *very close* relation with the alter (0=*less than very close*). For direct interaction frequency, 1 represents a situation where egos and alters meet *at least twice a month* (0=*less than twice a month*). We used the product of the 2 binary variables to derive another binary variable, dubbed *ego-alter intensity*, where 1 represents the situation in which the ego feels very close to the alter, *and* they meet at least twice a month.

For the relations between alters, the participants were presented with the following question for each alter dyad: “Please tell me if these persons, even if they are related to each other, are acquaintances, casual friends, or close friends.” From the alter-alter tie data, we derived *structural* network-level and node-level measurements after the ego was excluded from the personal network, its presence being redundant. All alter-alter ties were binarized, 1 representing the existence of a relation while 0 denoting situations where alters do not know each other.

Node-level structural measures are represented by alters’ *betweenness centrality* and *vaccination assortativity score*. Betweenness centrality quantifies the number of times a node acts as a bridge along the shortest path between 2 other nodes [75]. Thus, betweenness centrality can also be viewed as the power to control the flow of information and opinions about vaccination inside the personal network [31]. The betweenness scores were normalized to account for differing network sizes.

The *vaccination assortativity score* combines attribute and relational data to account for subgroupings inside the personal network. It informs whether alters of similar traits (vaccinated or not) tend to share a social tie. This score was computed for each alter *i*, from the personal network of an ego *j*, taking the difference between the proportion of all of the *j*’s alters that are connected to *i* and vaccinated and the proportion of all of the *j*’s alters that are vaccinated. In computing the proportions, *i* was not included. For example, let us suppose that ego *j* has nominated 25 alters, of whom 14 (56%) are vaccinated against COVID-19 and 11 (44%) are not. In this personal network, alter *i,* who is vaccinated*,* is directly connected with 9 other alters, of which 6 (67%) are vaccinated and 3 (33%) are unvaccinated. The assortativity score for alter *i* (which is excluded from the computation) is as follows:

6/9 – 13/24 = 0.666 – 0.542 = 0.142.

This indicates that the alter’s *i* network neighbors have an above-average vaccination rate compared to the overall proportion of vaccinated alters in the personal network. Given that an alter’s vaccination status is measured via a binary variable (1=vaccinated and 0=unvaccinated), a positive correlation coefficient indicates assortative mixing (ie, the alter’s vaccination status is related to the vaccination status of its direct neighbors in the network). The computation method was previously advanced to detect assortative mixing of opinions related to COVID-19 vaccination [31] and processed food intake [76].

Network-level measurements describe personal networks as a whole, accounting for the distribution of alter-alter ties. Network *size* represents the number of nodes inside a network (in our case, the alters). *Density* sums up the proportion of present ties out of the theoretical number of ties (ie, if all nodes are connected). Density can also be interpreted as a measure of cohesion inside the network [77]. Finally, the *number of components* is an indicator of network fragmentation. An unfragmented network is a network with 1 component where all nodes are directly or indirectly connected to each other. If a network has *n≥2* components, it indicates the number of *n* clusters of alters that are separated from each other. Furthermore, isolated nodes represent stand-alone components [75].

### Statistical Analysis

#### Overview

Regarding the statistical modeling, the objective of our analyses was to assess the impact of egos’ online information consumption in predicting alters’ vaccination status: vaccinated or not. Given the nature of the dependent variable (binary) and the nested structure of the data, alters embedded in egos’ networks, a mixed multilevel logistic regression model with random intercepts was used [78]. In this framework, alter-level data and ego-alter relations can be regarded as level 1 predictors (individual), while egos’ attributes and network-level data are level 2 predictors (group) [79]. This type of nesting must be controlled for, as the data about egos and alters are reported by egos, for which unobserved covariates could influence the results [31]. Level 1 predictors include alters’ sex, education level, age, relationship status (being single), betweenness centrality, relation intensity with ego, and vaccination assortativity. Level 2 predictors include egos’ sex, education, age, employment status, and media use alongside network size, density, and number of strong components. To avoid scaling problems in the estimation of models, numeric variables were mean centered and standardized. Even if the education variable was measured via an ordinal scale, it was introduced in the statistical model as a numeric variable, given its large interval (from 1 to 13), and to let it have variation.

#### Data Exclusion

The initial sample comprised 83 respondents, with varying network sizes and missing data about alters’ being vaccinated against COVID-19. To ensure that we have enough data at each level 2 group, we chose to focus our analyses only on those personal networks that have ≥15 alters for whom data about vaccination were present and who are not isolates. Not being an isolate (having ≥1 tie with another alter) is an important condition, given that isolated alters, for which the assortativity score could not be computed (as they have no network neighbors), were dropped from the analysis. After filtering the data according to these conditions, we ended up with a subset of 64 personal networks. Of the 64 personal networks, the regression models and some bivariate analyses presented in Multimedia Appendix 1 considered only 61 (95%) egos because data about media use for health and prevention were missing for 3 (5%) of them. Egos’ relationship status was not introduced in the multilevel regression models, given the vast overrepresentation of those who were not single. Of the 61 egos, only 7 (11%) reported that they were single.

## Results

### Descriptive Statistics

Table 1 presents descriptive statistics for characteristics of egos, personal networks, and alters.

Most respondents (egos; 50/64, 78%) were vaccinated. They had an average age of approximately 53 years (SD 51.86), were almost equally split with respect to sex (33/64, 52% were female respondents), a large majority (56/64, 88%) were in a relationship (being married or as a couple), and a little more than half (36/64, 56%) were employed. The average value for education is 9.7 (SD 1.78), in between secondary (finished high school) and postsecondary (nontertiary) education on the ordinal scale. Distribution of responses for the type of media used for information about health and prevention shows that most egos used a combination of online and traditional media (29/64, 45%), 21 (33%) out of 64 used online media exclusively, and 11 (17%) used only traditional media as their information sources. The respondents’ social contacts (alters) had a similar average age of approximately 53 (SD 16.06) years, similar average education (mean 9.35, SD 1.93), and approximately equally split on their sex (820/1561, 52.5% were female). Furthermore, similar to egos, the vast majority (1242/1561, 80%) of alters were in a relationship. Regarding their vaccination status, 64% (991/1561) of egos’ social contacts were reported as being vaccinated against COVID-19. For relations between egos and their social contacts, we report that for 22% (347/1561; mean 0.22, SD 0.42) of ego-alter ties, the respondents mentioned that they were “very close” and met at least “twice a month” with the respective alter.

**Table 1:** Descriptive statistics for egos and alters.

Characteristic			Ego and network data (n=64)		Alter data (n=1561)
Age (y), mean (SD)			53.33 (15.86)		52.64 (16.06)
**Education level**					
	Mean (SD)	9.70 (1.78; 0^a^)		9.35 (1.93; 52^a^)	
	Median (IQR)	10 (2; 0^a^)		9 (2; 52^a^)	
**Vaccinated against COVID-19, n (%)**					
	No	14 (21.88)		394 (25.24)	
	Yes	50 (78.12)		991 (63.48)	
	Missing data	0 (0)		176 (11.27)	
**Sex, n (%)**					
	Male	31 (48.44)		741 (47.47)	
	Female	33 (51.56)		820 (52.53)	
**Single, n (%)**					
	No	56 (87.5)		1242 (79.56)	
	Yes	8 (12.5)		318 (20.37)	
	Missing data	0 (0)		1 (0.06)	
**Employed, n (%)**					
	No	28 (43.75)		—^b^	
	Yes	36 (56.25)		—	
**Media used for health and prevention, n (%)**					
	Traditional media	11 (17.19)		—	
	Online media	21 (32.81)		—	
	Both media	29 (45.31)		—	
	Missing data	3 (4.69)		—	
Ego-alter intensity, mean (SD)			—		0.22 (0.42)
Alter betweenness centrality, mean (SD)			—		0.04 (0.07)
Vaccination assortativity, mean (SD)			—		0.02 (0.11; 181^a^)
Network size, mean (SD)			24.39 (1.70)		—
Network density, mean (SD)			0.65 (0.21)		—
Network components, mean (SD)			1.16 (0.51)		—

^a^Missing data (represents the number of cases for which the information is unknown).

^b^Not available.

Summary statistics for personal networks’ structural features show that, on average, they are composed of 24 alters (56/64, 88% have ≥24 alters), while the rest have 15 (1/64, 2%), 20 (3/64, 5%), 22 (2/64, 3%), and 23 (2/64, 3%) alters, with an average density of 0.65 (SD 0.21) and 1.16 (SD 0.51) number of strong components, indicating high cohesiveness (density) and low fragmentation (components). A mean betweenness score of 0.04 (SD 0.07; scaled by the network’s size) tells us that the alters had a low level of control over the potential information flow between the otherwise unconnected alters. The mean assortativity score for vaccination is 0.02 (SD 0.11), indicating that, on average, the proportion of an alter’s direct network neighbors who were vaccinated is similar to the overall proportion inside the personal network (minus the alter of reference).

More detailed descriptive statistics regarding the distributions of presented variables are provided in Tables S3, S4, S7, and S13 in Multimedia Appendix 1. Bivariate tests for some of the presented variables are also reported in Multimedia Appendix 1 . First, looking at the alters’ vaccination status contingent on egos’ vaccination status, we observe that the frequency distributions are not independent. Vaccinated respondents tended to have more vaccinated social contacts in their personal networks (*χ^2^*_1_=76.2; *P*<.001; Table S15 in Multimedia Appendix 1). Second, the frequency distribution of the alters’ vaccination status by egos’ type of media used for health information also indicates a nonindependent distribution (*χ^2^*_2_=28.2; *P*<.001; Table S16 in Multimedia Appendix 1). Egos using online media only present the highest proportion of unvaccinated alters (172/457, 37.6%), compared with egos using only traditional media (74/284, 29.8%) or both (140/616, 22.7%). However, it must be mentioned that there was no association between the egos’ vaccination status and their preferred media for information about health and prevention distribution (*χ^2^*_2_=2.5, Fisher *P*=.32 Table S17 in Multimedia Appendix 1). Two-sided *t* tests of independent samples, comparing the average assortativity score grouped by alters’ or egos’ vaccination status, showed that in both cases, assortative mixing was lower for unvaccinated alters (t_1378_=–2.3835; *P*=.02; Table S19 in Multimedia Appendix 1) and unvaccinated egos (t_1378_=–3.5391; *P*<.001; Table S21 in Multimedia Appendix 1). Similarly, by performing a chi-square test comparing the distribution of alters’ assortativity quartiles by egos’ media use and vaccination status, we found that the distribution is nonindependent (*χ*^2^_15_=83.2; *P*<.001; Table S22 in Multimedia Appendix 1). Egos who were unvaccinated and used only online media present the highest proportion of alters in quartiles 1 (33/106, 31.1%) and 2 (43/106, 40.6%).

### Statistical Modeling

In Table 2, we report the results of the full mixed multilevel logistic regression model, using alters’ vaccination status as an outcome variable for 61 groups and 1280 alters (ie, cases without missing data on the variables included in the model).

Egos’ relationship status (being single or not) was not included in the models due to its lack of variation. The intraclass correlation coefficient of 0.2 indicates that we have within-group homogeneity and intergroup heterogeneity. The grouping dependence is also confirmed by the marginal and conditional *R*^2^ coefficients. The fixed effects explain 21.5% of the variance, while the random effects account for an extra 15.9% of the total variance. Regarding alters’ attributes, education increases the probability of an alter being vaccinated (odds ratio [OR] 1.87, 95% CI 1.58-2.22; *P*<.001), while being single lowers it (OR 0.67, 95% CI 0.46-0.96; *P*=.03). Among egos’ attributes as level 2 predictors, we observed that being in the personal network of an interviewee who was vaccinated increases an alter’s chance of also being vaccinated (OR 3.75, 95% CI 1.79-7.85; *P*<.001). Network-level structural characteristics, such as size (OR 0.96, 95% CI 0.74-1.26; *P*=.79), density (OR 1.23, 95% CI 0.87-1.74; *P*=.24), and number of components (OR 1.04, 95% CI 0.77-1.41; *P*=.80), have no significant effects on the outcome variable. Between the node-level structural features (alter’s betweenness centrality and vaccination assortativity), only the variable accounting for assortative mixing presents a positive effect (OR 1.17, 95% CI 1.01-1.35; *P*=.04). For every SD increase in the assortativity score, alters are 17% more likely to be vaccinated.

**Figure 1:**
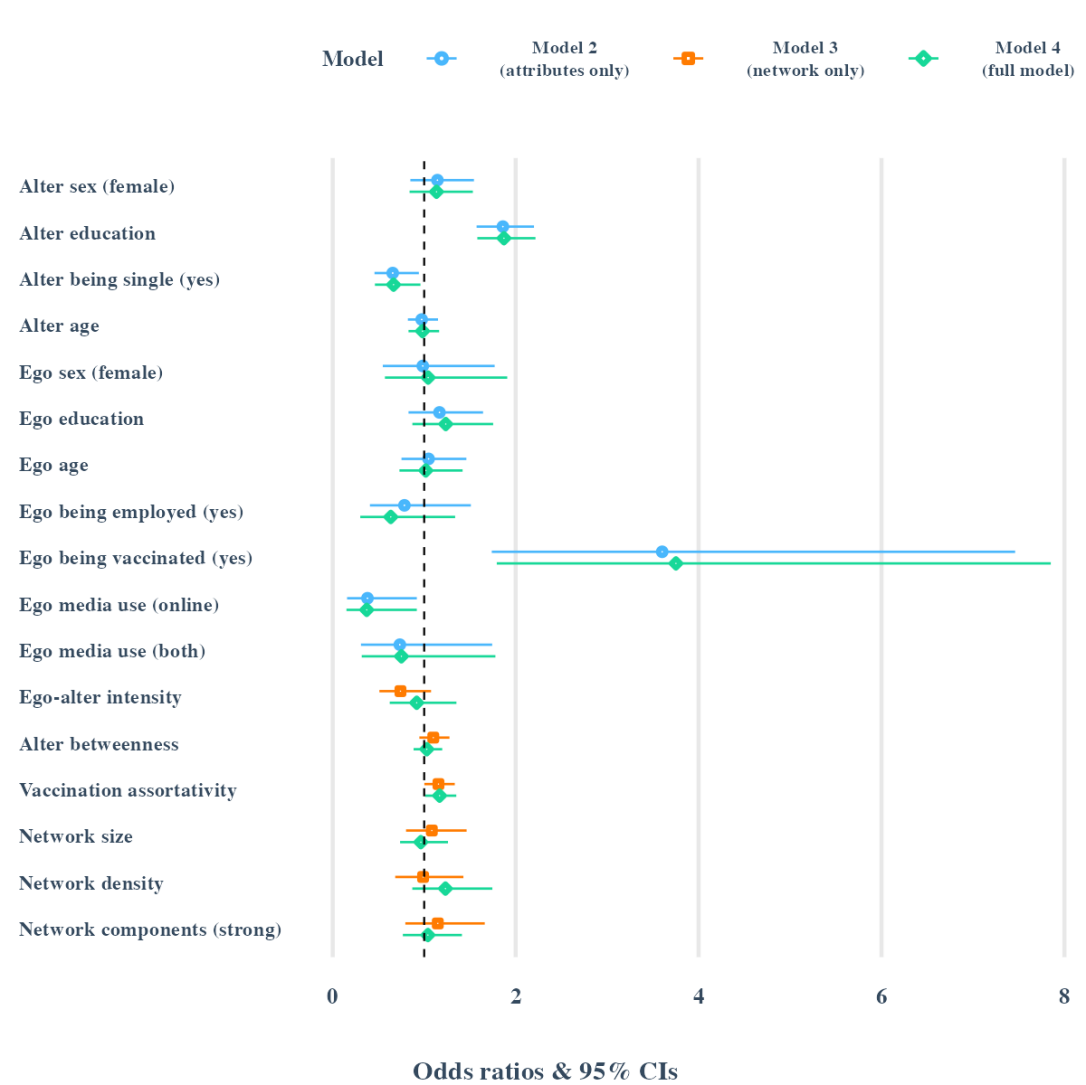
Comparison of mixed multilevel logistic regression models. Contrasts between the attributes only model (model 2), network only model (model 3), and the full model (model 4).

Egos’ media use for health and prevention information is our variable of interest, using traditional media as the reference category. Results indicate a negative effect for switching from the exclusive use of legacy media to the exclusive use of online media (OR 0.37, 95% CI 0.15-0.92; *P*=.03). The change from “traditional only” to “both” types of media has no effect (*P*=.52). For comparison, Figure 1 presents a visual summary of multiple models, with ORs and 95% CIs. Model 2 represents a model where we included only predictors referring to egos and alters’ attributes. Model 3 is a model where we included only predictors that consider relational data, such as ego-alter relation intensity, node-level structural features (betweenness centrality and vaccination assortativity), and network-level structural features. Table S23 in Multimedia Appendix 1 presents in detail the full model (model 4) in comparison with the null model (model 1) and models 2 (attributes only) and 3 (network only). The variable of interest, that is the ego’s media use for health information, remained significant in model 2 (OR 0.38, 95% CI 0.16-0.92; *P*=.03) and model 4 (OR 0.37, 95% CI 0.15-0.92; *P*=.03), with similar odds levels while controlling for the other level 1 and 2 factors.

Besides the multilevel logistic regression models, we also performed a logistic general linear model with robust SEs (ie, SEs clustered by egos). Table S24 in Multimedia Appendix 1 presents the following results. Comparing the full model from Table 2 with the full model from Table S24 (general linear model 4), we observed similar results with regards to alters’ education (OR 1.82, 95% CI 1.46-2.26; *P*<.001), egos being vaccinated (OR 1.34, 95% CI 1.06-1.68; *P*=.01), egos using online media for information about health (OR 0.66, 95% CI 0.44-0.97; *P*=.04), and alters’ vaccination assortativity (OR 1.21, 95% CI 1.01-1.46; *P*=.04). To account for not having random ego intercepts, this model also included the proportion of vaccinated alters minus the alter of interest. Furthermore, for this predictor, we observed a positive and significant effect (OR 2.48, 95% CI 2.05-3.01; *P*<.001).

**Table 2:** Full mixed multilevel logistic regression model^a^ predicting alters’ vaccination status (n=1280 alters and 61 egos).

Predictors^b^				OR^c^ (95% CI)		*P* value
Intercept				2.29 (0.85-6.15)		.10
**Alter level (1)**						
	**Sex**					
		Male	Reference		—^d^	
		Female	1.13 (0.84-1.53)		.41	
	Education			1.87 (1.58-2.22)		<.001
	**Single**					
		No	Reference		—	
		Yes	0.67 (0.46-0.96)		.03	
	Alter age			0.98 (0.83-1.16)		.83
	Ego-alter intensity			0.92 (0.62-1.35)		.66
	Alter betweenness			1.03 (0.88-1.20)		.71
	Vaccination assortativity			1.17 (1.01-1.35)		.04
**Ego level (2)**						
	**Sex**					
		Male	Reference		—	
		Female	1.04 (0.57-1.91)		.89	
	Education			1.24 (0.87-1.75)		.24
	Age			1.02 (0.73-1.42)		.92
	**Employed**					
		No	Reference		—	
		Yes	0.63 (0.30-1.34)		.23	
	**Vaccinated**					
		No	Reference		—	
		Yes	3.75 (1.79-7.85)		<.001	
	**Media type**					
		Traditional	Reference		—	
		Online	0.37 (0.15-0.92)		.03	
		Both	0.75 (0.32-1.78)		.52	
**Network level (2)**						
	Network size			0.96 (0.74-1.26)		.79
	Network density			1.23 (0.87-1.75)		.24
	Network components (strong)			1.04 (0.77-1.41)		.80

^a^Model indices: variance=0.83 (SD 0.913); intraclass correlation coefficient=0.2; observations=1280; groups=61; marginal *R*^2^=0.215; and conditional *R*^2^=0.374.

^b^With the exception of categorical variables and ego-alter intensity, all variables were mean-centered and scaled. The outcome variable remained in its original units.

^c^OR: odds ratio.

^d^Reference category.

## Discussion

### Principal Findings

Our study investigated the impact of online media consumption on COVID-19 vaccination behaviors using a PNA approach. We aimed to explore the influence of real-world network structures and compositions on vaccine hesitancy, with a particular focus on the role of individuals’ (egos’) reliance on online media for health and prevention information as the primary predictor. Through the application of mixed multilevel logistic regression models, we assessed the vaccination status of individuals (alters) within 61 personal networks. Our findings indicate that alters within networks of egos who exclusively use online media for health and prevention information are less likely to be vaccinated compared to those in networks where egos depend on traditional media sources. In addition, for alters in networks where egos access both online and traditional media, the effect on vaccination status was not statistically significant. Our results are in line with previous studies that suggest social media use lowers COVID-19 vaccine acceptance [13,19]. The results of the mixed multilevel logistic regression are also supported by general linear models using robust SEs.

Moreover, our results support European-level statistics, which indirectly measure digital health literacy. In Romania, in 2023, the proportion of individuals who had at least basic digital skills was 31.09% [66]; however, the proportion of individuals who used internet for social media platforms was at 84.35% [67], and only 32.83% reported seeing, in online spaces, information or content which was untrue or doubtful (compared with the EU average of 53.67%) [68]. Secondary results from our analyses relate to some of the alters’ sociodemographic characteristics and egos’ vaccination status. Alters’ level of education and relationship status (being in a relationship) increased their odds of being vaccinated, as found in systematic reviews and meta-analyses [80,81]. Similarly, being in the personal network of an ego who is vaccinated increased the odds of alters being vaccinated, which is in line with another PNA study on a sample of Romanian respondents, testing for the assortative mixing of opinions on the COVID-19 vaccine and finding a positive association [31]. In this context, online misinformation and disinformation campaigns can find fertile ground and be enhanced by social influence in real-world networks when they mirror such opinions.

The ways individuals use the internet and the effects of information obtained through this medium, when it comes to health opinions and behaviors, are diverse. For example, Moon et al [82] found that while a higher frequency of social media use increased the odds of hesitancy, the higher frequency of internet use (for all purposes) decreased them. Allington et al [18] found that social media use loses its effect on vaccine hesitancy when controlling for trust in government, health professionals, and scientists and the COVID-19 pandemic is perceived as a risk. In our models, using both types of media did not differ from using only traditional media, presenting a nonsignificant effect in predicting alters’ vaccination status. On one hand, this could indicate that exposure to multiple information sources can decrease hesitancy. On the other hand, this also indicates exogenous factors that are not considered when discussing online behavior. In our supplementary analyses, the media used by egos for health and prevention are not associated with their vaccination status. This could be due to the small sample size of 61 respondents.

An additional finding from our study highlights the impact of assortative mixing on vaccination outcomes. Specifically, alters who had a higher proportion of vaccinated direct network neighbors, relative to the vaccination rate of the entire network, were more likely to be vaccinated themselves. This finding aligns with other research using PNA to examine assortative mixing concerning attitudes toward COVID-19 vaccination [31] and processed food consumption [76]. Such alignment underlines the potential for further exploratory and comparative studies. Notably, our additional analyses reveal that networks characterized by lower vaccination assortativity tend to belong to unvaccinated egos, particularly those relying solely on online media for information and unvaccinated alters. This pattern suggests a fragmentation in personal networks concerning health beliefs and behaviors, possibly exacerbated by the diverse opinions and information sources about COVID-19 vaccines, echoing the WHO’s concept of an “infodemic” [9]. The fact that the lower assortativity scores are found inside personal networks of unvaccinated egos who use only online media can be further hypothesized as an indicator of reinforcement of online misinformation through network contagion.

Given the limited evidence of the effects of real-world networks on vaccination hesitancy in relation to online media use, it remains an open question whether vaccine hesitancy is the result of assortative mixing (homogeneity) or social mixing (heterogeneity), when we look at networks’ compositions and structures based on the nodes’ media use. Building on the available literature about network influences on health behaviors and opinions [31,43,50,51], the working hypothesis would be that assortative mixing on multidimensional aspects of online behavior (eg, used platforms, followed accounts, and eHealth literacy) would positively correlate with assortative mixing on vaccination status. In our results, the similarity between egos’ and their alters’ vaccination status, coupled with the fact that the elicited alters were not randomly selected by respondents, can be an indicator of this association. The study of personal networks presents the possibility of the *false consensus effect* or *egocentric attribution bias*, wherein individuals attribute to others’ opinions and behaviors similar to their own [83]. This also can add to our conclusions, as individuals can be influenced in their behavior not by what others do but by what they perceive as being the others’ behaviors and opinions.

Other studies have found that a person’s social relations influence their degree of COVID-19 vaccine hesitancy [52-64] on various aspects, including willingness to receive vaccine and trust or opinions about its efficacy and safeness. Such studies use various measures of individual social capital or assortative mixing (often dubbed as “homophily”) through standard social surveys or qualitative interviews. Our study goes beyond the compositional aspect of a person’s social circle and, by using a PNA design, also captures its structural features. In this way, characteristics such as network density and the number of strong components are controlled for in our models. Furthermore, it allowed us to go beyond the ego-alter relations captured by standard social surveys and look at the effect of alters’ assortative mixing.

The generalization of our findings warrants careful consideration due to the distinct methodological and contextual facets of our study. The data, collected from a rural community in Romania (Eastern Europe) using a link-tracing sampling methodology, provide insights into the dynamics of online media influence on vaccination behaviors within personal networks. However, the rural setting and the specific sociocultural context of Eastern Europe might limit the direct applicability of our results to urban settings or other regions with different digital literacy levels and media consumption patterns. Nevertheless, it is important to underline that our findings likely reflect the situation in rural areas throughout Eastern Europe, thereby enhancing the inclusivity and relevance of our study within this broader regional context. Future research could benefit from applying similar methodological approaches in diverse settings to explore the universality and variability of these patterns across different social, cultural, and geographical contexts.

The implications raised by our study are not confined to COVID-19 vaccine hesitancy but vaccine hesitancy in general, in offline and online spaces, as real-world personal networks combine both. Further insights on how the information obtained by individuals from online spaces spreads and is reinforced through personal networks are needed, especially for countries with low general, digital, and eHealth literacy. Such countries are more vulnerable in the face of future pandemic disinformation campaigns regarding other vaccines and need communication campaigns and vaccination programs adapted to community-specific patterns.

### Limitations

Our study has several limitations. Some of them are inherent to the PNA design itself. The information about alters’ attributes and alter-alter relations is reported by the ego, which can lead to inaccuracies [84]. Therefore, the data generation process requires a careful interpretation of the results. To mitigate the impact of inaccuracies in ego-reported data, we limited the number of alters to 25, used communication or meeting frequency as a criterion for the name generator, and used the ego-alter relation intensity (emotional closeness and meeting frequency) as a control variable. If possible, future research should account for overlapping alters and use this information to correct contradictory accounts about alters’ attributes; for example, see what the majority consensus about an alter’s vaccination status is if that person is present in other personal networks.

Furthermore, we must consider challenges related to our primary variable of interest: the types of media used for health and prevention information. First, this variable was assessed solely at the ego level, because it can lead to potential inaccuracies when egos report on their alters’ media use. Such inaccuracies could be more pronounced compared to reporting on demographic information (eg, age, education level, and marital status) or vaccination status against COVID-19. Second, our analysis does not differentiate between social media platforms and other online media sources, such as news aggregators (eg, Google News and Yahoo News). Prior research indicates divergent impacts on vaccine hesitancy and acceptance between these sources, with social media use often correlated with increased vaccine hesitancy, whereas broader internet use or reliance on online news platforms tends to be associated with higher vaccine acceptance [20,27,82]. The conflation of social media with other online media in our respondents’ reports precludes a nuanced analysis of these distinct influences within our dataset.

Another limitation is the fact that we did not collect and analyze data on ego-alter interactions in various online spaces. Such data can bring further insights into the social selection or influence mechanisms that may occur in the online dimension of personal networks. Furthermore, we did not collect and analyze data of egos’ or alters’ motives and opinions regarding COVID-19 vaccination. Thus, our study did not differentiate between persons who were vaccinated because of noncoercive or coercive reasons (eg, receiving a vaccine against COVID-19 due to work-related obligations and falling in the “hesitant adopter” category on the vaccine hesitance spectrum [63]). Including such attribute data in future analyses can control for distinctions between personal networks, that is, differentiating between networks where alters’ vaccination rate is low, yet some egos received the vaccine while other egos did not.

Furthermore, the link-tracing sampling method can introduce selection biases regarding respondents and individuals recommended for further participation. Potential selection biases can appear in various forms. Specific to our study, we can speculate that the nature of the 4P-CAN project, as a project about primary prevention, can attract more health-conscious individuals, skewing the results. This kind of selection effect was observed regarding COVID-19 vaccination, the general tendency being that vaccinated participants referred those who were also vaccinated. Future studies that use a network-oriented sampling method may implement a strategy of selecting seeds by considering both the vaccination status and their sociodemographic characteristics, which are associated with vaccine acceptance or hesitancy, to increase diversity. However, this does not necessarily guarantee the elimination of selection biases.

Further biases could have also been introduced by the incentives given for participation, such as free access to a local educational program focused on health topics and a hotline number that the participants can use if they want expert opinions regarding medical problems or second opinions regarding a medical diagnostic, without any other monetary incentives. However, every decision in terms of incentives, including nonmonetary, monetary, or none, has the potential to skew the sample with reference to the sociodemographic composition and retention inside the panel. Furthermore, the research project is applied inside a living lab, an open ecosystem entailing the community’s coparticipation. Therefore, the purpose is to raise awareness of medical prevention and, at the same time, offer persons the health information and access that they lack in rural areas. Simultaneously, one of the objectives was to create a panel of respondents with a high retention rate during subsequent waves by combining the link-tracing referee-referral chains with an incentive structure that had the potential to attract persons who were either more health conscious or who sought medical advice.

The size of the sample, of 61 personal networks with valid data on all variables of interest, can also skew the results. Despite these limitations, the presence of assortative mixing regarding COVID-19 vaccine opinions was also identified in another study with a larger sample size of 443 Romanian respondents, using a different sampling strategy [31]. Furthermore, the general interpretation of our results aligns with studies that used nonnetwork designs and sampling strategies and found that varying degrees of vaccine hesitancy are influenced by a person’s level of individual social capital [52-56] or perceived vaccination status of family and friends [57,58]. This consistency across studies suggests the robustness of our findings despite the methodological challenges.

### Conclusions

Our study suggests that health and prevention information disseminated through online media, including social media, significantly impacts individuals’ vaccination status within real-world personal networks. In addition to assortative mixing effects, our findings highlight the positive influence of education on vaccination status. We advocate for a deeper exploration of the composition and structure of personal networks, emphasizing the need to understand individuals’ online behaviors and digital literacy levels for designing effective vaccination communication campaigns and interventions. Romania presents a case study of particular interest where low digital literacy and vaccination rates coincide with high social media use, underscoring the challenges of combating online misinformation and disinformation in similar contexts.

## Appendix

Multimedia Appendix 1 Detailed descriptive statistics, statistical models, and R code.

## Abbreviations

EU: European Union
OR: odds ratio
PNA: personal network analysis
SAGE: Strategic Advisory Group of Experts on Immunization
SNA: social network analysis
WHO: World Health Organization

## Data Availability

The datasets generated during and analyzed during this study are available in the Zenodo repository [85].
