# Supplementary material for: Online Media Use and COVID-19 Vaccination in Real-World Personal Networks: Quantitative Study
Source: J Med Internet Res. 2024 Oct 25;26:e58257. doi: 10.2196/58257 (PMC11549583; doi:10.2196/58257)
Supplement: Multimedia Appendix 1 [file jmir_v26i1e58257_app1.pdf]

Contents

Libraries needed to run the code . . . 4

1. Load data **4**

1.1 About data frames: . . . 4

1.2 About variables: . . . 4

1.3 Variables' names and labels: . . . 5

table S1 . . . 5

table S2 . . . 6

2. Descriptive statistics **7**

2.1 Egos: numeric variables of interest and network level features . . . 7

table S3 . . . 7

2.2 Alters: numeric variables of interest and ego-alter relation intensity . . . 7

table S4 . . . 7

2.3 Egos: categorical variables of interest . . . 8

table S5 . . . 8

table S6 . . . 9

table S7 . . . 9

table S8 . . . 10

table S9 . . . 10

table S10 . . . 10

2.4 Alters: categorical variables of interest . . . 11

table S11 . . . 11

table S12 . . . 11

table S13 . . . 12

table S14 . . . 12

3. Bivariate analyses **13**

3.1 Contingency table for egos' and alters' vaccination status . . . 13

table S15 . . . 13

3.2 Contingency table for egos' media use and alters' vaccination status . . . 13

table S16 . . . 13

3.3 Contingency table for egos' media use and their vaccination status . . . 14

table S17 . . . 14

3.3 T-test of independent samples: vaccination assortativity by alters' vaccination status 15

table S18 . . . 15

table S19 . . . 15

3.4 T-test of independent samples: vaccination assortativity by egos' vaccination status 16

table S20 . . . 16

table S21 . . . 16

3.5 Contingency table for alters' assortativity quartile by egos' media use x egos' vaccination status . . . 17

table S22 . . . 17

4. Multilevel logistic regression models estimation: alters-alter ties between friends **18**

4.1 Null model (Model 1) . . . 18

4.2 Attributes model (Model 2) . . . 18

4.3 Network model (Model 3) . . . 18

4.4 Full model (Model 4) . . . 19

4.5 Print results . . . 19

table S23 . . . 19

figure S1 . . . 21

5. Model diagnostics **23**
5.1 Predicting alters' vaccination status using GLM with robust clustered errors . . . 23
5.1.1 GLM model 1 23
5.1.2 GLM model 2 23
5.1.3 GLM model 3 23
5.1.4 GLM model 4 24
5.1.5 Print results 24
table S24 24
5.2 Multicolliniarity 25
5.2.1 VIF scores for multilevel models 25
table S25 25
table S26 25
table S27 26
5.2.2 VIF scores for GLM models with clustered errors 27
table S28 27
table S29 27
table S30 27
5.3 Predictive performance 28
5.3.1 ROC curve for multilevel models 28
figure S2 28
figure S3 29
figure S4 29
figure S5 30
5.3.1 ROC curve for GLM models with clustered errors 30
figure S6 30
figure S7 31
figure S8 31
figure S9 32
5.4 Residual plots 33
5.4.1 Residual plots for multilevel models 33
figure S10 33
figure S11 33
figure S12 34
figure S13 34
5.4.2 Residual plots for GLM models with clustered errors 35
figure S14 35
figure S15 35
figure S16 36
figure S17 36
5.5 Overdispersion estimation 37
5.5.1 Residual plots for multilevel models 37
table S31 37
figure S18 37
table S32 38
figure S19 38
table S33 39
figure S20 39
table S34 40
figure S21 40
5.5.2 Residual plots for GLM models with clustered errors 41
table S35 41
figure S22 41
table S36 42
figure S23 42

table S37 . . . 43
figure S24 . . . 43
table S38 . . . 44
figure S25 . . . 44
5.6 Plots of the residuals for random effects in multilevel models . . . 45
figure S26 . . . 45
figure S27 . . . 46
figure S28 . . . 47
figure S29 . . . 48

6. Miscellaneous (Eurostat data) **49**
6.1 Eurostat data from dataset "Individuals' level of digital skills (from 2021 onwards)" 49
figure S30 . . . 49
figure S31 . . . 51
6.2 Eurostat data from dataset "Individuals - internet activities" . . . 52
figure S32 . . . 52
figure S33 . . . 54
6.3 Eurostat data from dataset "Evaluating data, information and digital content (2021 onwards)" . . . 55
figure S34 . . . 55
figure S35 . . . 57
figure S36 . . . 58
figure S37 . . . 59
figure S38 . . . 60
figure S39 . . . 61
figure S40 . . . 62
figure S41 . . . 63
figure S42 . . . 64

This is the Multimedia Appendix 1 (supplementary material) file corresponding to the manuscript titled: **Online Media Use and COVID-19 Vaccination in Real-World Personal Networks: Quantitative Study**.

This document contains the `R code` for replicating the results mentioned in the study. We divide this text into several sections corresponding to descriptive statistics, model estimates, model diagnostics, and miscellaneous.

Libraries needed to run the code

```
library(tidyverse)
library(knitr)
# for kableExtra a version lower than 1.4 is needed! Otherwise, the <scale_down> function
↪  might not work
library(kableExtra)
library(broom)
library(broom.mixed)
library(lme4)
library(summarytools)
library(report)
library(rempsyc)
library(Hmisc)
library(scales)
library(MASS)
library(jtools)
library(car)
library(sjPlot)
library(janitor)
library(miceadds)
library(pROC)
library(spsUtil)
library(eurostat)
library(stringi)
```

1. Load data

```
# read ego data
ego_data <- readRDS("ego_data.rds")

# read alter data
alter_data <- readRDS("alter_data.rds")
```

1.1 About data frames:

- the data frames used in this script are divided into **ego data** and **alter data**.
- ego data frames contain data about the egos and personal networks' structure and composition.
- alter data frames contain data about the egos, alters, and personal networks' structure.

1.2 About variables:

- the suffix "**.f**" indicates columns where variables are stored as factors.
- the suffix "**.mc**" indicates columns where variables are stored as scaled (mean centered) variables.
- columns not ending in "**.f**" or "**.mc**" are numeric, storing the variables' original score.

- the prefix "**prop_**" refers to data describing proportions of certain features inside personal networks.

1.3 Variables' names and labels:

table S1

```r
#
variables_labels <-  data.frame(Hmisc::label(ego_data))
#
variables_labels <- variables_labels %>% rownames_to_column()
#
colnames(variables_labels) <- c("Variable", "Variable label")
#
variables_labels %>%
  kbl(caption = "Variable names and labels in ego data",
      booktabs = T, linesep = "") %>%
  kable_styling(full_width = T, latex_options = c("hold_position", "scale_down")) %>%
  column_spec(1, width = "5cm", italic = T) %>%
  row_spec(0, bold = T)
```

Table S1: Variable names and labels in ego data

**Variable**	**Variable label**
networkCanvasEgoUUID	Ego's alphanumeric ID generated by Network Canvas
ego_code	Ego's numeric ID
ego_ID	Ego's alphanumeric ID
ego.vaccination.f	Ego's vaccination status (as factor)
ego.sex.f	Ego's sex (as factor)
ego.age	Ego's age
ego.age.mc	Ego's age, mean centered and scaled
ego.education.f	Ego's education (as factor)
ego.education	Ego's education (as numeric)
ego.education.mc	Ego's education, mean centered and scaled
ego.single.f	Ego being single (as factor)
ego.employed.f	Ego being employed (as factor)
ego.media.use.f	Ego's media use for health information and prevention (as factor)
network_size	Personal network size
network_size.mc	Personal network size, mean centered and scaled
network_density	Personal network density
network_density.mc	Personal network density, mean centered and scaled
network_components	Number of strong components in the personal network
network_components.mc	Number of strong components in the personal network, mean centered and scaled
sum_vaccinated	Number of alters inside the personal network who are vaccinated
prop_vaccinated	Proportion of alters inside the personal network who are vaccinated
prop_female	Proportion of alters inside the personal network who are female
prop_single	Proportion of alters inside the personal network who are single
mean_age	Average age of alters inside the personal network
median_age	Median age of alters inside the personal network
mean_education	Average education of alters inside the personal network
median_education	Median education of alters inside the personal network
mean_intensity	Average ego-alter tie intensity inside the personal network

table S2

```r
#
variables_labels <-  data.frame(Hmisc::label(alter_data))
#
variables_labels <- variables_labels %>% rownames_to_column()
#
colnames(variables_labels) <- c("Variable", "Variable label")
#
variables_labels %>%
  kbl(caption = "Variable names and labels in alter data",
      booktabs = T, linesep = "") %>%
  kable_styling(full_width = F, latex_options = c("hold_position", "scale_down")) %>%
  column_spec(1, width = "5cm", italic = T) %>%
  row_spec(0, bold = T)
```

Table S2: Variable names and labels in alter data

**Variable**	**Variable label**
networkCanvasEgoUUID	Ego's alphanumeric ID generated by Network Canvas
networkCanvasUUID	Alter's alphanumeric ID generated by Network Canvas
ego_code	Ego's numeric ID
ego_ID	Ego's alphanumeric ID
alter_code	Alter's numeric ID
alter_ID	Alter's alphanumeric ID
alter.vaccination.f	Alter's vaccination status (as factor)
alter.sex.f	Alter's sex (as factor)
alter.age	Alter's age
alter.age.mc	Alter's age, mean centered and scaled
alter.education.f	Alter's education (as factor)
alter.education	Alter's education (as numeric)
alter.education.mc	Alter's education, mean centered and scaled
alter.single.f	Alter being single (as factor)
ego.vaccination.f	Ego's vaccination status (as factor)
ego.sex.f	Ego's sex (as factor)
ego.age	Ego's age
ego.age.mc	Ego's age, mean centered and scaled
ego.education.f	Ego's education (as factor)
ego.education	Ego's education (as numeric)
ego.education.mc	Ego's education, mean centered and scaled
ego.single.f	Ego being single (as factor)
ego.employed.f	Ego being employed (as factor)
ego.media.use.f	Ego's media use for health information and prevention (as factor)
ego.alter.intensity	Intensity of Ego-Alter tie
alter.betweenness	Alter's betweenness centrality score
alter.betweenness.mc	Alter's betweenness centrality score, mean centered and scaled
alter.degree	Alter's degreee centrality score
alter.degree.mc	Alter's degree centrality score, mean centered and scaled
vaccination.assortativity	Alter's vaccination assortativity score
vaccination.assortativity.mc	Alter's vaccination assortativity score, mean centered and scaled
prop.vacc.alters_ex.alter	Proportion of vaccinated alters inside the personal network excluding row alter
prop.vacc.alters_ex.alter.mc	Proportion of vaccinated alters inside the personal network excluding row alter, mean centered and scaled
vaccinated.neighbours	Number of alter's direct contacts who are vaccinated
total.neighbours	Total number of alter's direct contacts
network_size	Personal network size
network_size.mc	Personal network size, mean centered and scaled
network_density	Personal network density
network_density.mc	Personal network density, mean centered and scaled
network_components	Number of strong components in the personal network
network_components.mc	Number of strong components in the personal network, mean centered and scaled

2. Descriptive statistics

2.1 Egos: numeric variables of interest and network level features

table S3

```
#
ego_desc_num <- ego_data %>%
  # select variables of interest
  dplyr::select(ego.age, ego.education,
                network_size, network_density,
                network_components) %>%
  # compute summary statistics
  summarytools::descr() %>%
  round(2) %>%
  as.data.frame() %>%
  # reorder columns
  dplyr::select(1, 2, 5, 4, 3)

colnames(ego_desc_num) <- c("Egos' age", "Egos' education", "Network size",
                            "Network density", "Network components")

ego_desc_num %>%
  kbl(caption = "Descriptive statistics for numeric variables:
      Ego characteristics and network level measures (level 2)",
      booktabs = T, linesep = "") %>%
  kable_classic_2(full_width = T) %>%
  column_spec(1, bold = T) %>%
  kable_styling(latex_options = "hold_position")
```

Table S3: Descriptive statistics for numeric variables: Ego characteristics and network level measures (level 2)

	Egos' age	Egos' education	Network size	Network density	Network components
**Mean**	53.33	9.70	24.39	0.65	1.16
**Std.Dev**	15.86	1.78	1.70	0.21	0.51
**Min**	18.00	5.00	15.00	0.25	1.00
**Q1**	44.00	9.00	25.00	0.49	1.00
**Median**	53.00	10.00	25.00	0.69	1.00
**Q3**	68.00	11.00	25.00	0.79	1.00
**Max**	80.00	12.00	25.00	1.00	4.00
**MAD**	20.76	1.48	0.00	0.20	0.00
**IQR**	24.00	2.00	0.00	0.30	0.00
**CV**	0.30	0.18	0.07	0.31	0.44
**Skewness**	-0.45	-0.92	-3.53	-0.26	3.76
**SE.Skewness**	0.30	0.30	0.30	0.30	0.30
**Kurtosis**	-0.76	0.28	13.94	-0.87	15.25
**N.Valid**	64.00	64.00	64.00	64.00	64.00
**Pct.Valid**	100.00	100.00	100.00	100.00	100.00

2.2 Alters: numeric variables of interest and ego-alter relation intensity

table S4

```
#
alter_desc_num <- alter_data %>%
  dplyr::select(alter.age, alter.education,
                ego.alter.intensity, alter.betweenness, alter.degree,
                vaccination.assortativity) %>%
  summarytools::descr() %>%
  round(2) %>%
  as.data.frame() %>%
  # reorder columns
  dplyr::select(1, 4, 5, 3, 2, 6)
```

```r
colnames(alter_desc_num) <- c("Alters' age", "Alters' education", "Ego-Alter intensity",
                              "Alters' degree", "Alters' betweenness", "Vaccination assortativity")

alter_desc_num %>%
  kbl(caption = "Descriptive statistics for numeric variables:
      Alter characteristics and ego-alter intensity (level 1)",
      booktabs = T, linesep = "") %>%
  kable_classic_2(full_width = T) %>%
  column_spec(1, bold = T) %>%
  kable_styling(latex_options = "hold_position")
```

Table S4: Descriptive statistics for numeric variables: Alter characteristics and ego-alter intensity (level 1)

	Alters' age	Alters' education	Ego-Alter intensity	Alters' degree	Alters' betweenness	Vaccination assortativity
**Mean**	52.64	9.35	0.22	0.04	0.04	0.02
**Std.Dev**	16.06	1.93	0.42	0.02	0.07	0.11
**Min**	18.00	2.00	0.00	0.00	0.00	-0.75
**Q1**	42.00	9.00	0.00	0.03	0.00	-0.01
**Median**	54.00	9.00	0.00	0.04	0.01	0.00
**Q3**	65.00	11.00	0.00	0.05	0.05	0.06
**Max**	92.00	13.00	1.00	0.16	0.75	0.71
**MAD**	16.31	2.97	0.00	0.01	0.02	0.06
**IQR**	23.00	2.00	0.00	0.01	0.05	0.08
**CV**	0.31	0.21	1.87	0.37	1.75	5.53
**Skewness**	-0.22	-1.03	1.33	0.47	4.28	-0.05
**SE.Skewness**	0.06	0.06	0.06	0.06	0.06	0.07
**Kurtosis**	-0.59	1.01	-0.22	3.80	28.15	8.75
**N.Valid**	1561.00	1509.00	1561.00	1561.00	1561.00	1380.00
**Pct.Valid**	100.00	96.67	100.00	100.00	100.00	88.40

2.3 Egos: categorical variables of interest

table S5

```r
#
results <- freq(ego_data$ego.vaccination.f) %>% as.data.frame()
rownames(results)[rownames(results) == "<NA>"] <- "Missing"
results <- results %>% replace(is.na(.), 0)
#
results %>% dplyr::select(-c(2, 3)) %>%
  kbl(digits = 2,
      caption = "Egos' vaccination status",
      booktabs = T, linesep = "") %>%
  kable_classic_2() %>%
  column_spec(1, bold = T) %>%
  kable_styling(latex_options = "hold_position")
```

Table S5: Egos' vaccination status

	Freq	% Total	% Total Cum.
**No**	14	21.88	21.88
**Yes**	50	78.12	100.00
**Missing**	0	0.00	100.00
**Total**	64	100.00	100.00

table S6

```
#
results <- freq(ego_data$ego.sex.f) %>% as.data.frame()
rownames(results)[rownames(results) == "<NA>"] <- "Missing"
results <- results %>% replace(is.na(.), 0)
#
results %>% dplyr::select(-c(2, 3)) %>%
  kbl(digits = 2,
      caption = "Egos' sex",
      booktabs = T, linesep = "") %>%
  kable_classic_2() %>%
  column_spec(1, bold = T) %>%
  kable_styling(latex_options = "hold_position")
```

Table S6: Egos' sex

	Freq	% Total	% Total Cum.
**Male**	31	48.44	48.44
**Female**	33	51.56	100.00
**Missing**	0	0.00	100.00
**Total**	64	100.00	100.00

table S7

```
#
results <- freq(ego_data$ego.education.f) %>% as.data.frame()
rownames(results)[rownames(results) == "<NA>"] <- "Missing"
results <- results %>% replace(is.na(.), 0)
#
results %>% dplyr::select(-c(2, 3)) %>%
  kbl(digits = 2,
      caption = "Egos' education - last finished level",
      booktabs = T, linesep = "") %>%
  kable_classic_2() %>%
  column_spec(1, bold = T) %>%
  kable_styling(latex_options = "hold_position")
```

Table S7: Egos' education - last finished level

	Freq	% Total	% Total Cum.
**No school**	0	0.00	0.00
**Less than primary school**	0	0.00	0.00
**Primary school**	0	0.00	0.00
**Less than secondary school**	0	0.00	0.00
**Secondary school**	3	4.69	4.69
**Arts & Crafts school**	0	0.00	4.69
**10 obligatory years**	7	10.94	15.62
**Highschool - unfinished**	1	1.56	17.19
**Highschool - finished (with diploma)**	15	23.44	40.62
**Post-highschool (non-tertiary)**	11	17.19	57.81
**Bachelor's degree or equivalent level**	20	31.25	89.06
**Master's degree or equivalent level**	7	10.94	100.00
**PhD or equivalent level**	0	0.00	100.00
**Missing**	0	0.00	100.00
**Total**	64	100.00	100.00

table S8

```
#
results <- freq(ego_data$ego.single.f) %>% as.data.frame()
rownames(results)[rownames(results) == "<NA>"] <- "Missing"
results <- results %>% replace(is.na(.), 0)
#
results %>% dplyr::select(-c(2, 3)) %>%
  kbl(digits = 2,
      caption = "Egos' being single",
      booktabs = T, linesep = "") %>%
  kable_classic_2() %>%
  column_spec(1, bold = T) %>%
  kable_styling(latex_options = "hold_position")
```

Table S8: Egos' being single

	Freq	% Total	% Total Cum.
**No**	56	87.5	87.5
**Yes**	8	12.5	100.0
**Missing**	0	0.0	100.0
**Total**	64	100.0	100.0

table S9

```
#
results <- freq(ego_data$ego.employed.f) %>% as.data.frame()
rownames(results)[rownames(results) == "<NA>"] <- "Missing"
results <- results %>% replace(is.na(.), 0)
#
results %>% dplyr::select(-c(2, 3)) %>%
  kbl(digits = 2,
      caption = "Egos' being employed",
      booktabs = T, linesep = "") %>%
  kable_classic_2() %>%
  column_spec(1, bold = T) %>%
  kable_styling(latex_options = "hold_position")
```

Table S9: Egos' being employed

	Freq	% Total	% Total Cum.
**Unemployed**	28	43.75	43.75
**Employed**	36	56.25	100.00
**Missing**	0	0.00	100.00
**Total**	64	100.00	100.00

table S10

```
#
results <- freq(ego_data$ego.media.use.f) %>% as.data.frame()
rownames(results)[rownames(results) == "<NA>"] <- "Missing"
results <- results %>% replace(is.na(.), 0)
#
results %>% dplyr::select(-c(2, 3)) %>%
  kbl(digits = 2,
      caption = "Egos' media use for information about health and prevention",
      booktabs = T, linesep = "") %>%
  kable_classic_2() %>%
  column_spec(1, bold = T) %>%
  kable_styling(latex_options = "hold_position")
```

Table S10: Egos' media use for information about health and prevention

	Freq	% Total	% Total Cum.
**Traditional media**	11	17.19	17.19
**Online media**	21	32.81	50.00
**Both media**	29	45.31	95.31
**Missing**	3	4.69	100.00
**Total**	64	100.00	100.00

2.4 Alters: categorical variables of interest

table S11

```
#
results <- freq(alter_data$alter.vaccination.f) %>% as.data.frame()
rownames(results)[rownames(results) == "<NA>"] <- "Missing"
results <- results %>% replace(is.na(.), 0)
#
results %>% dplyr::select(-c(2, 3)) %>%
  kbl(digits = 2,
      caption = "Alters' vaccination status",
      booktabs = T, linesep = "") %>%
  kable_classic_2() %>%
  column_spec(1, bold = T) %>%
  kable_styling(latex_options = "hold_position")
```

Table S11: Alters' vaccination status

	Freq	% Total	% Total Cum.
**No**	394	25.24	25.24
**Yes**	991	63.48	88.73
**Missing**	176	11.27	100.00
**Total**	1561	100.00	100.00

table S12

```
#
results <- freq(alter_data$alter.sex.f) %>% as.data.frame()
rownames(results)[rownames(results) == "<NA>"] <- "Missing"
results <- results %>% replace(is.na(.), 0)
#
results %>% dplyr::select(-c(2, 3)) %>%
  kbl(digits = 2,
      caption = "Alters' sex",
      booktabs = T, linesep = "") %>%
  kable_classic_2() %>%
  column_spec(1, bold = T) %>%
  kable_styling(latex_options = "hold_position")
```

Table S12: Alters' sex

	Freq	% Total	% Total Cum.
**Male**	741	47.47	47.47
**Female**	820	52.53	100.00
**Missing**	0	0.00	100.00
**Total**	1561	100.00	100.00

table S13

```r
#
results <- freq(alter_data$alter.education.f) %>% as.data.frame()
rownames(results)[rownames(results) == "<NA>"] <- "Missing"
results <- results %>% replace(is.na(.), 0)
#
results %>% dplyr::select(-c(2, 3)) %>%
  kbl(digits = 2,
      caption = "Alters' education - last finished level",
      booktabs = T, linesep = "") %>%
  kable_classic_2() %>%
  column_spec(1, bold = T) %>%
  kable_styling(latex_options = "hold_position")
```

Table S13: Alters' education - last finished level

	Freq	% Total	% Total Cum.
**No school**	0	0.00	0.00
**Less than primary school**	4	0.26	0.26
**Primary school**	15	0.96	1.22
**Less than secondary school**	10	0.64	1.86
**Secondary school**	61	3.91	5.77
**Arts & Crafts school**	41	2.63	8.39
**10 obligatory years**	128	8.20	16.59
**Highschool - unfinished**	54	3.46	20.05
**Highschool - finished (with diploma)**	486	31.13	51.19
**Post-highschool (non-tertiary)**	144	9.22	60.41
**Bachelor's degree or equivalent level**	474	30.37	90.78
**Master's degree or equivalent level**	84	5.38	96.16
**PhD or equivalent level**	8	0.51	96.67
**Missing**	52	3.33	100.00
**Total**	1561	100.00	100.00

table S14

```r
#
results <- freq(alter_data$alter.single.f) %>% as.data.frame()
rownames(results)[rownames(results) == "<NA>"] <- "Missing"
results <- results %>% replace(is.na(.), 0)
#
results %>% dplyr::select(-c(2, 3)) %>%
  kbl(digits = 2,
      caption = "Alters' being single",
      booktabs = T, linesep = "") %>%
  kable_classic_2() %>%
  column_spec(1, bold = T) %>%
  kable_styling(latex_options = "hold_position")
```

Table S14: Alters' being single

	Freq	% Total	% Total Cum.
**No**	1242	79.56	79.56
**Yes**	318	20.37	99.94
**Missing**	1	0.06	100.00
**Total**	1561	100.00	100.00

3. Bivariate analyses

3.1 Contingency table for egos' and alters' vaccination status

table S15

```
#
results <- sjt.xtab(alter_data$ego.vaccination.f,
         alter_data$alter.vaccination.f,
         show.row.prc = T,
         show.col.prc = F,
         show.na = F,
         show.summary = F)
# convert results to data frame
results = sjtable2df::xtab2df(results)
# rename columns
colnames(results) <- c("Egos' vaccination status", "No", "Yes", "Total")
# print results
results %>% kbl(caption = "Contingency table for egos' and alters' vaccination status",
                booktabs = T, linesep = "") %>%
  add_header_above(c(" " = 1, "Alters' vaccination status" = 2, " " = 1)) %>%
  kable_styling(latex_options = "hold_position")
```

Table S15: Contingency table for egos' and alters' vaccination status

	Alters' vaccination status		
Egos' vaccination status	No	Yes	Total
No	148 (48.5 %)	157 (51.5 %)	305 (100 %)
Yes	246 (22.8 %)	834 (77.2 %)	1080 (100 %)
Total	394 (28.4 %)	991 (71.6 %)	1385 (100 %)

```
# print chi-squared results
#
chisqtable = (table(alter_data$ego.vaccination.f,
                    alter_data$alter.vaccination.f))
#
chisq.test(chisqtable)
```

chi-squared test for table S15

```
Pearson's Chi-squared test with Yates' continuity correction
```

data: chisqtable X-squared = 76.195, df = 1, p-value < 0.00000000000000022

3.2 Contingency table for egos' media use and alters' vaccination status

table S16

```
#
results <- sjt.xtab(alter_data$ego.media.use.f,
         alter_data$alter.vaccination.f,
         show.row.prc = T,
         show.col.prc = F,
         show.na = F,
         show.summary = F)
# convert results to data frame
results = sjtable2df::xtab2df(results)
# rename columns
colnames(results) <- c("Egos' media use", "No", "Yes", "Total")
# print results
results %>% kbl(caption = "Contingency table for egos' media use and alters' vaccination status",
                booktabs = T, linesep = "") %>%
  add_header_above(c(" " = 1, "Alters' vaccination status" = 2, " " = 1)) %>%
  kable_styling(latex_options = "hold_position")
```

Table S16: Contingency table for egos' media use and alters' vaccination status

	Alters' vaccination status		
Egos' media use	No	Yes	Total
Traditional media	74 (29.8 %)	174 (70.2 %)	248 (100 %)
Online media	172 (37.6 %)	285 (62.4 %)	457 (100 %)
Both media	140 (22.7 %)	476 (77.3 %)	616 (100 %)
Total	386 (29.2 %)	935 (70.8 %)	1321 (100 %)

```
# print chi-squared results
#
chisqtable = (table(alter_data$ego.media.use.f,
                    alter_data$alter.vaccination.f))
#
chisq.test(chisqtable)
```

chi-squared test for table S16

Pearson's Chi-squared test

data: chisqtable X-squared = 28.255, df = 2, p-value = 0.0000007319

3.3 Contingency table for egos' media use and their vaccination status

table S17

```
#
results <- sjt.xtab(ego_data$ego.media.use.f,
         ego_data$ego.vaccination.f,
         show.row.prc = T,
         show.col.prc = F,
         show.na = F,
         show.summary = F)
# convert results to data frame
results = sjtable2df::xtab2df(results)
# rename columns
colnames(results) <- c("Egos' media use", "No", "Yes", "Total")
# print results
results %>% kbl(caption = "Contingency table for egos' media use by their vaccination status",
                booktabs = T, linesep = "") %>%
  add_header_above(c(" " = 1, "Egos' vaccination status" = 2, " " = 1)) %>%
  kable_styling(latex_options = "hold_position")
```

Table S17: Contingency table for egos' media use by their vaccination status

	Egos' vaccination status		
Egos' media use	No	Yes	Total
Traditional media	4 (36.4 %)	7 (63.6 %)	11 (100 %)
Online media	5 (23.8 %)	16 (76.2 %)	21 (100 %)
Both media	4 (13.8 %)	25 (86.2 %)	29 (100 %)
Total	13 (21.3 %)	48 (78.7 %)	61 (100 %)

```
# print chi-squared results
#
chisqtable = (table(ego_data$ego.media.use.f,
                    ego_data$ego.vaccination.f))
#
chisq.test(chisqtable)
```

chi-squared test for table S17

```
Pearson's Chi-squared test
```

data: chisqtable X-squared = 2.5418, df = 2, p-value = 0.2806

```r
fisher.test(chisqtable)
```

```
Fisher's Exact Test for Count Data
```

data: chisqtable p-value = 0.3154 alternative hypothesis: two.sided

3.3 T-test of independent samples: vaccination assortativity by alters' vaccination status

table S18

```r
# test for variance by group
lt <- car::leveneTest(vaccination.assortativity ~ alter.vaccination.f, data = alter_data)
lt <- data.frame(lt) %>% mutate(df = lt[1,1]) %>% mutate(df2 = lt[2,1]) %>% filter(!is.na(F.value))
lt <- lt %>% dplyr::select(-c(1)) %>% rename("F value" = F.value, "P value" = Pr..F.)
row.names(lt) <- NULL

#
lt %>%
  kbl(caption = "Levene test of variance equality", booktabs = T, digits = 5) %>%
  kable_classic() %>%
  kable_styling(latex_options = "hold_position", full_width = F)
```

Table S18: Levene test of variance equality

F value	P value	df	df2
0.0021	0.96343	1	1378

```r
# variance equal: P>.05; variances unequal: P<0.05
```

table S19

```r
results <- t.test(vaccination.assortativity ~ alter.vaccination.f,
       data = alter_data,
       var.equal = T)
# select necessary columns
t.table <- as.data.frame(report(results)) %>%
  dplyr::select(3:11)
# rename columns
colnames(t.table) <- c("Mean - unvaccinated group", "Mean - vaccinated group",
                       "Mean difference", "CI", "CI low", "CI high", "t", "df", "p")
# print results
t.table %>%
  kbl(digits = 4,
      caption = "Vaccination assortativity by alters' vaccination status - independent samples t-test (two-sided)",
      booktabs = T, linesep = "") %>%
  kable_styling(latex_options = "hold_position", full_width = F) %>%
  # row_spec(0, align = "l") %>%
  column_spec(1, width = "3cm") %>%
  column_spec(2, width = "2.7cm") %>%
  row_spec(0, align = "l")
```

Table S19: Vaccination assortativity by alters' vaccination status - independent samples t-test (two-sided)

Mean - unvaccinated group	Mean - vaccinated group	Mean difference	CI	CI low	CI high	t	df	p
0.009	0.025	-0.016	0.95	-0.0293	-0.0028	-2.3835	1378	0.0173

3.4 T-test of independent samples: vaccination assortativity by egos' vaccination status

table S20

```
# test is variances are equal by group
lt <- car::leveneTest(vaccination.assortativity ~ ego.vaccination.f, data = alter_data)
lt <- data.frame(lt) %>% mutate(df = lt[1,1]) %>% mutate(df2 = lt[2,1]) %>% filter(!is.na(F.value))
lt <- lt %>% dplyr::select(-c(1)) %>% rename("F value" = F.value, "P value" = Pr..F.)
row.names(lt) <- NULL

#
lt %>%
  kbl(caption = "Levene test of variance equality", booktabs = T, digits = 5) %>%
  kable_classic() %>%
  kable_styling(latex_options = "hold_position", full_width = F)
```

Table S20: Levene test of variance equality

F value	P value	df	df2
5.72347	0.01687	1	1378

```
# variance equal: P>.05; variances unequal: P<0.05
```

table S21

```
results <- t.test(vaccination.assortativity ~ ego.vaccination.f,
       data = alter_data,
       var.equal = T)
# select necessary columns
t.table <- as.data.frame(report(results)) %>%
  dplyr::select(3:11)
# rename columns
colnames(t.table) <- c("Mean - unvaccinated group", "Mean - vaccinated group",
                      "Mean difference", "CI", "CI low", "CI high", "t", "df", "p")
# print results
t.table %>%
  kbl(digits = 4,
      caption = "Vaccination assortativity by egos' vaccination status - independent samples t-test (two-sided)",
      booktabs = T, linesep = "") %>%
  kable_styling(latex_options = "hold_position", full_width = F) %>%
  # row_spec(0, align = "l") %>%
  column_spec(1, width = "3cm") %>%
  column_spec(2, width = "2.7cm") %>%
  row_spec(0, align = "l")
```

Table S21: Vaccination assortativity by egos' vaccination status - independent samples t-test (two-sided)

Mean - unvaccinated group	Mean - vaccinated group	Mean difference	CI	CI low	CI high	t	df	p
0.0003	0.0262	-0.0259	0.95	-0.0402	-0.0115	-3.5391	1378	0.0004

3.5 Contingency table for alters' assortativity quartile by egos' media use x egos' vaccination status

table S22

```
# create data frame with needed variables
my_tab <- alter_data %>%
  dplyr::select(networkCanvasEgoUUID, networkCanvasUUID,
                ego.media.use.f, ego.vaccination.f,
                alter.vaccination.f, vaccination.assortativity) %>%
  # remove egos with missing data on the media use variable
  filter(!is.na(ego.media.use.f)) %>%
  # remove alters with missing data on the vaccination variable
  filter(!is.na(alter.vaccination.f)) %>%
  # remove alters with missing data on the vaccination assortativity variable
  filter(!is.na(vaccination.assortativity)) %>%
  # create variable combining egos' vaccination status and media use
  mutate(ego.media.use.f = as.character(ego.media.use.f)) %>%
  mutate(ego.vaccination.f = as.character(ego.vaccination.f)) %>%
  mutate(ego.media.vaccinated = paste(ego.media.use.f, ego.vaccination.f, sep = "-")) %>%
  # rename categories
  mutate(ego.media.vaccinated = str_replace_all(ego.media.vaccinated, "Both media-No",
                                     "Unvaccinated & uses both media")) %>%
    mutate(ego.media.vaccinated = str_replace_all(ego.media.vaccinated, "Both media-Yes",
                                     "Vaccinated & uses both media")) %>%
    mutate(ego.media.vaccinated = str_replace_all(ego.media.vaccinated, "Online media-No",
                                     "Unvaccinated & uses online media")) %>%
    mutate(ego.media.vaccinated = str_replace_all(ego.media.vaccinated, "Online media-Yes",
                                     "Vaccinated & uses online media")) %>%
    mutate(ego.media.vaccinated = str_replace_all(ego.media.vaccinated, "Traditional media-No",
                                     "Unvaccinated & uses traditional media")) %>%
    mutate(ego.media.vaccinated = str_replace_all(ego.media.vaccinated, "Traditional media-Yes",
                                     "Vaccinated & uses traditional media")) %>%
  mutate(ego.media.vaccinated = factor(ego.media.vaccinated)) %>%
  # create quartiles for alters' vaccination assortativity
  mutate(assortativity.quartile = ntile(vaccination.assortativity, 4))
```

```
# print results
results <- sjt.xtab(my_tab$ego.media.vaccinated,
         my_tab$assortativity.quartile,
         show.row.prc = T,
         show.col.prc = F,
         show.na = F,
         show.summary = F)
# convert results to data frame
results = sjtable2df::xtab2df(results)
# rename columns
colnames(results) <- c("Egos' vaccination status & media use",
                       "Quartile 1", "Quartile 2", "Quartile 3", "Quartile 4", "Total")
# add needed spaces
results <- results %>% mutate(`Egos' vaccination status & media use` = str_replace_all(
  `Egos' vaccination status & media use`, "uses", "uses "
))
# print results
results %>% kbl(caption = "Alters' assortativity quartile by egos' vaccination status x media use",
                booktabs = T, linesep = " ") %>%
  add_header_above(c(" " = 1, "Alters' assortativity quartile" = 4, " " = 1)) %>%
  kable_styling(latex_options = c("hold_position", "scale down"), full_width = F, font_size = 7)
```

Table S22: Alters' assortativity quartile by egos' vaccination status x media use

	Alters' assortativity quartile				
Egos' vaccination status & media use	Quartile 1	Quartile 2	Quartile 3	Quartile 4	Total
Unvaccinated & uses both media	28 (32.2 %)	9 (10.3 %)	24 (27.6 %)	26 (29.9 %)	87 (100 %)
Unvaccinated & uses online media	33 (31.1 %)	43 (40.6 %)	19 (17.9 %)	11 (10.4 %)	106 (100 %)
Unvaccinated & uses traditional media	19 (21.8 %)	34 (39.1 %)	12 (13.8 %)	22 (25.3 %)	87 (100 %)
Vaccinated & uses both media	123 (23.3 %)	133 (25.2 %)	116 (22 %)	155 (29.4 %)	527 (100 %)
Vaccinated & uses online media	95 (27.2 %)	55 (15.8 %)	116 (33.2 %)	83 (23.8 %)	349 (100 %)
Vaccinated & uses traditional media	31 (19.4 %)	55 (34.4 %)	42 (26.2 %)	32 (20 %)	160 (100 %)
Total	329 (25 %)	329 (25 %)	329 (25 %)	329 (25 %)	1316 (100 %)

```
# print chi-squared results
```

```
#
chisqtable = (table(my_tab$ego.media.vaccinated,
                    my_tab$assortativity.quartile))
#
chisq.test(chisqtable)
```

chi-squared test for table S22

Pearson's Chi-squared test

data: chisqtable X-squared = 83.229, df = 15, p-value = 0.00000000001785

4. Multilevel logistic regression models estimation: alters-alter ties between friends

4.1 Null model (Model 1)

```
m1 <- lme4::glmer(
  alter.vaccination.f # Dependent variable (alter's vaccination status)
   ~ 1 +
    (1 | networkCanvasEgoUUID), # Intercept (1) varies in level-2 units (egos)
  family = binomial("logit"), # Model class (logistic)
  data = na.omit(alter_data),
  set.seed(1234),
  nAGQ = 100,
  control = glmerControl(optimizer = "bobyqa",
                         optCtrl = list(maxfun = 2e5))
)
```

4.2 Attributes model (Model 2)

```
#
m2 <- lme4::glmer(
  alter.vaccination.f # Dependent variable (alter's vaccination status)

    ### alter attributes
    ~ alter.sex.f
    + alter.education.mc
    + alter.single.f
    + alter.age.mc

    ### ego attributes
    + ego.sex.f
    + ego.education.mc
    + ego.age.mc
    + ego.employed.f
    + ego.vaccination.f

    ### ego media use
    + ego.media.use.f
    +
    (1 | networkCanvasEgoUUID), # Intercept (1) varies in level-2 units (egos)
  family = binomial("logit"), # Model class (logistic)
  data = na.omit(alter_data),
  set.seed(1234),
  nAGQ = 100,
  control = glmerControl(optimizer = "bobyqa",
                         optCtrl = list(maxfun = 2e5)))
```

4.3 Network model (Model 3)

```
#
m3 <- lme4::glmer(
  alter.vaccination.f # Dependent variable (alter's vaccination status)

    ### network data: ego-alter relation
    ~ ego.alter.intensity

    ### network data: node level features
    + alter.betweenness.mc
    + vaccination.assortativity.mc
```

```
    ### network data: network level features
    + network_size.mc
    + network_density.mc
    + network_components.mc
    +
    (1 | networkCanvasEgoUUID), # Intercept (1) varies in level-2 units (egos)
  family = binomial("logit"), # Model class (logistic)
  data = na.omit(alter_data),
  set.seed(1234),
  nAGQ = 100,
  control = glmerControl(optimizer = "bobyqa",
                         optCtrl = list(maxfun = 2e5)))
```

4.4 Full model (Model 4)

```
m4 <- lme4::glmer(
  alter.vaccination.f # Dependent variable (alter's vaccination status)

    ### alter attributes
    ~ alter.sex.f
    + alter.education.mc
    + alter.single.f
    + alter.age.mc

    ### ego attributes
    + ego.sex.f
    + ego.education.mc
    + ego.age.mc
    + ego.employed.f
    + ego.vaccination.f

    ### ego media use
    + ego.media.use.f

    ### network data: ego-alter relation
    + ego.alter.intensity

    ### network data: node level features
    + alter.betweenness.mc
    + vaccination.assortativity.mc

    ### network data: network level features
    + network_size.mc
    + network_density.mc
    + network_components.mc
    +

    (1 | networkCanvasEgoUUID), # Intercept (1) varies in level-2 units (egos)
  family = binomial("logit"), # Model class (logistic)
  data = na.omit(alter_data),
  set.seed(1234),
  nAGQ = 100,
  control = glmerControl(optimizer = "bobyqa",
                         optCtrl = list(maxfun = 2e5))
)
```

4.5 Print results

```
# Create data frame with all models
# -- create an  object with all the models
table.models = sjPlot::tab_model(m1, m2, m3, m4,
                                 show.r2 = T, show.aic = T, show.loglik = T)
# -- transform the object into a data frame
table.models_df = sjtable2df::mtab2df(table.models, n_models = 4) %>% as.data.frame()
# Run code from "multilevel_labels.R" script to apply transformations to the data frame in order
# to recode labels and attach missing model fit estimates
source("multilevel_labels.R")
```

table S23

```
table.models_df %>%
  kable(col.names = c("Predictors", "Odds Ratios", "95% CIs", "P value", "Odds Ratios", "95% CIs", "P value",
                      "Odds Ratios", "95% CIs", "P value", "Odds Ratios", "95% CIs", "P value"),
```

```
      caption = "Mixed multilevel regression models predicting alters' vaccination status",
      booktabs = T, linesep = "") %>%
  kable_classic() %>%
  add_header_above(c(" " = 1, "Model 1\n(null model)" = 3,
                     "Model 2\n(attributes only)" = 3,
                     "Model 3\n(network only)" = 3,
                     "Model 4\n(full model)" = 3)) %>%
  kable_styling(latex_options = c("hold_position", "scale_down", "striped"), full_width = F) %>%
  row_spec(18,  hline_after = T)
```

Table S23: Mixed multilevel regression models predicting alters' vaccination status

	Model 1 (null model)			Model 2 (attributes only)			Model 3 (network only)			Model 4 (full model)		
Predictors	Odds Ratios	95% CIs	P value	Odds Ratios	95% CIs	P value	Odds Ratios	95% CIs	P value	Odds Ratios	95% CIs	P value
Intercept	3.02	2.13-4.28	<0.001	2.13	0.80-5.67	0.129	3.27	2.27-4.69	<0.001	2.29	0.85-6.15	0.102
Alter sex (female)				1.14	0.85-1.54	0.376				1.13	0.84-1.53	0.410
Alter education				1.86	1.57-2.20	<0.001				1.87	1.58-2.22	<0.001
Alter being single (yes)				0.66	0.46-0.94	0.022				0.67	0.46-0.96	0.029
Alter age				0.97	0.82-1.15	0.747				0.98	0.83-1.16	0.831
Ego sex (female)				0.98	0.55-1.77	0.958				1.04	0.57-1.91	0.890
Ego education				1.17	0.83-1.64	0.377				1.24	0.87-1.75	0.236
Ego age				1.05	0.75-1.46	0.782				1.02	0.73-1.42	0.918
Ego being employed (yes)				0.78	0.41-1.51	0.467				0.63	0.30-1.34	0.232
Ego being vaccinated (yes)				3.60	1.74-7.46	0.001				3.75	1.79-7.85	<0.001
Ego media use (online)				0.38	0.16-0.92	0.032				0.37	0.15-0.92	0.032
Ego media use (both)				0.73	0.31-1.74	0.483				0.75	0.32-1.78	0.515
Ego-Alter intensity							0.74	0.51-1.07	0.114	0.92	0.62-1.35	0.665
Alter betweenness							1.10	0.95-1.28	0.212	1.03	0.88-1.20	0.713
Vaccination assortativity							1.16	1.00-1.33	0.047	1.17	1.01-1.35	0.036
Network size							1.08	0.80-1.46	0.603	0.96	0.74-1.26	0.786
Network density							0.99	0.68-1.43	0.949	1.23	0.87-1.75	0.239
Network components (strong)							1.15	0.79-1.66	0.462	1.04	0.77-1.41	0.798
Random effects												
Variance (SD)	3.29 (1.247)			3.29 (0.93)			3.29 (1.235)			3.29 (0.915)		
ICC	0.32			0.21			0.32			0.20		
AIC	1362.835			1284.721			1366.635			1290.974		
BIC	1373.144			1351.731			1407.872			1388.911		
Log Likelihood (DF)	-679.418 (2)			-629.361 (13)			-675.318 (8)			-626.487 (19)		
Observations	1280			1280			1280			1280		
Groups	61			61			61			61		
Marginal R2 / Conditional R2	0.000 / 0.321			0.208 / 0.373			0.015 / 0.327			0.215 / 0.374		

figure S1

```
#
jtools::plot_summs(m2, m3, m4,
                   coefs = c(
                    "Alter sex (female)" = "alter.sex.fFemale", "Alter education" = "alter.education.mc",
                    "Alter being single (yes)" = "alter.single.fYes", "Alter age" = "alter.age.mc",
                    "Ego sex (female)" = "ego.sex.fFemale", "Ego education" = "ego.education.mc",
                    "Ego age" = "ego.age.mc", "Ego being employed (yes)" = "ego.employed.fEmployed",
                    "Ego being vaccinated (yes)" = "ego.vaccination.fYes",
                    "Ego media use (online)" = "ego.media.use.fOnline media",
                    "Ego media use (both)" = "ego.media.use.fBoth media",
                    "Ego-Alter intensity" = "ego.alter.intensity", "Alter betweenness" = "alter.betweenness.mc",
                    "Vaccination assortativity" = "vaccination.assortativity.mc",
                    "Network size" = "network_size.mc", "Network density" = "network_density.mc",
                    "Network components (strong)" = "network_components.mc"),
                   exp = TRUE,
                   model.names = c("Model 2\n(attributes only)",
                                   "Model 3\n(network only)",
                                   "Model 4\n(full model)")) +
  theme(plot.margin = margin(t = 0.75,  # Top margin
                             r = 0,  # Right margin
                             b = 0,  # Bottom margin
                             l = 0,  # Left margin
                             unit = "cm"),
        axis.title.x = element_text(margin = margin(t = 10),
                                    hjust = 0.43, family = "Times"),
        # text=element_text(family="Times"),
        axis.text.x = element_text(family="Times", face = "bold"),
        axis.text.y = element_text(family="Times", hjust = 0),
        legend.text = element_text(family="Times", face = "bold", hjust = 0.5),
        legend.title = element_text(family="Times")) +
  labs(x = "Odds ratios & 95% CIs")
```

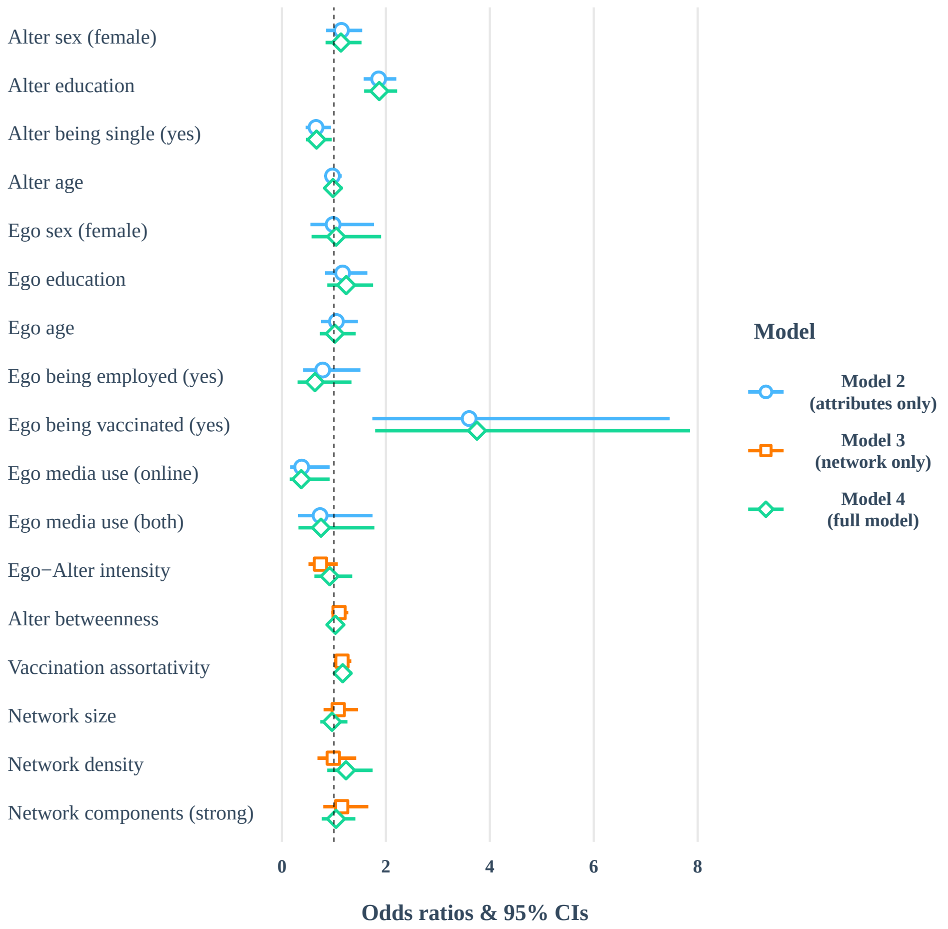

Figure S1: Summary plot of multilevel models

5. Model diagnostics

5.1 Predicting alters' vaccination status using GLM with robust clustered errors

5.1.1 GLM model 1

```
#
m1.glm <- miceadds::glm.cluster(alter.vaccination.f # Dependent variable (alter's vaccination status)
              ~ 1
              ### control for proportion of unvaccinated alters (minus the alter of interest)
              + prop.vacc.alters_ex.alter.mc,

              ### cluster errors by ego's ID & set random number seed for reproducibility of results
              cluster="networkCanvasEgoUUID",
              data = na.omit(alter_data),
              set.seed(1234),
              family = "binomial"
)
```

5.1.2 GLM model 2

```
#
m2.glm <- miceadds::glm.cluster(alter.vaccination.f # Dependent variable (alter's vaccination status)
    ### alter attributes
    ~ alter.sex.f
    + alter.education.mc
    + alter.single.f
    + alter.age.mc

    ### ego attributes
    + ego.sex.f
    + ego.education.mc
    + ego.age.mc
    + ego.employed.f
    + ego.vaccination.f

    ### ego media use
    + ego.media.use.f

    ### control for proportion of unvaccinated alters (minus the alter of interest)
    + prop.vacc.alters_ex.alter.mc,

    ### cluster errors by ego's ID & set random number seed for reproducibility of results
    cluster="networkCanvasEgoUUID",
    data = na.omit(alter_data),
    set.seed(1234),
    family = "binomial"
)
```

5.1.3 GLM model 3

```
#
m3.glm <- miceadds::glm.cluster(alter.vaccination.f # Dependent variable (alter's vaccination status)
    ### network data: ego-alter relation
    ~ ego.alter.intensity

    ### network data: node level features
    + alter.betweenness.mc
    + vaccination.assortativity.mc

    ### network data: network level features
    + network_size.mc
    + network_density.mc
    + network_components.mc

    ### control for proportion of unvaccinated alters (minus the alter of interest)
    + prop.vacc.alters_ex.alter.mc,

    # cluster errors by ego's ID & set random number seed for reproducibility of results
    cluster="networkCanvasEgoUUID",
    data = na.omit(alter_data),
    set.seed(1234),
    family = "binomial"
)
```

5.1.4 GLM model 4

```
#
m4.glm <- miceadds::glm.cluster(alter.vaccination.f # Dependent variable (alter's vaccination status)
    ### alter attributes
    ~ alter.sex.f
    + alter.education.mc
    + alter.single.f
    + alter.age.mc

    ### ego attributes
    + ego.sex.f
    + ego.education.mc
    + ego.age.mc
    + ego.employed.f
    + ego.vaccination.f

    ### ego media use
    + ego.media.use.f

    ### network data: ego-alter relation
    + ego.alter.intensity

    ### network data: node level features
    + alter.betweenness.mc
    + vaccination.assortativity.mc

    ### network data: network level features
    + network_size.mc
    + network_density.mc
    + network_components.mc

    ### control for proportion of unvaccinated alters (minus the alter of interest)
    + prop.vacc.alters_ex.alter.mc,

    ### cluster errors by ego's ID & set random number seed for reproducibility of results
    cluster="networkCanvasEgoUUID",
    data = na.omit(alter_data),
    set.seed(1234),
    family = "binomial"
)
```

5.1.5 Print results

```
# Run code from "glm_labels.R" script to apply transformations to the data frames in order to create a single table, recode
↪  labels, and attach missing model fit estimates
source("glm_labels.R")
```

table S24

```
all.glm %>%
  kable(col.names = c("Predictors",
                      "Odds Ratios",
                      "95% CIs",
                      "P value",
                      "Odds Ratios",
                      "95% CIs",
                      "P value",
                      "Odds Ratios",
                      "95% CIs",
                      "P value",
                      "Odds Ratios",
                      "95% CIs",
                      "P value"),
        caption = "GLM regression models predicting alters' vaccination status",
        booktabs = T, linesep = "") %>%
  kable_classic() %>%
  add_header_above(c(" " = 1, "GLM Model 1\n(null model)" = 3,
                     "GLM Model 2\n(attributes only)" = 3,
                     "GLM Model 3\n(network only)" = 3,
                     "GLM Model 4\n(full model)" = 3)) %>%
  row_spec(19,  hline_after = T) %>%
  kable_styling(latex_options = c("hold_position", "scale_down", "striped"), full_width = F)
```

Table S24: GLM regression models predicting alters' vaccination status

	GLM Model 1 (null model)			GLM Model 2 (attributes only)			GLM Model 3 (network only)			GLM Model 4 (full model)		
Predictors	Odds Ratios	95% CIs	P value	Odds Ratios	95% CIs	P value	Odds Ratios	95% CIs	P value	Odds Ratios	95% CIs	P value
Intercept	2.89	2.56-3.26	<.001	3.02	2.08-4.4	<.001	3.05	2.61-3.57	<.001	3.46	2.37-5.04	<.001
Alter sex (female)				1.16	0.89-1.51	0.275				1.12	0.86-1.47	0.391
Alter education				1.81	1.46-2.24	<.001				1.82	1.46-2.26	<.001
Alter being single (yes)				0.69	0.45-1.05	0.084				0.67	0.43-1.04	0.076
Alter age				1	0.81-1.23	0.983				1	0.81-1.22	0.972
Ego sex (female)				1	0.79-1.26	0.972				0.96	0.77-1.2	0.712
Ego education				0.95	0.81-1.11	0.517				0.94	0.81-1.1	0.443
Ego age				0.95	0.79-1.14	0.583				0.9	0.75-1.07	0.226
Ego being employed (yes)				0.85	0.63-1.15	0.288				0.82	0.58-1.14	0.237
Ego being vaccinated (yes)				1.42	1.09-1.85	0.01				1.34	1.06-1.68	0.013
Ego media use (online)				0.68	0.44-1.04	0.078				0.66	0.44-0.97	0.037
Ego media use (both)				0.9	0.59-1.38	0.628				0.93	0.62-1.4	0.732
Ego-Alter intensity							0.81	0.56-1.18	0.28	0.91	0.61-1.37	0.653
Alter betweenness							1.09	0.95-1.27	0.223	1.03	0.88-1.21	0.674
Vaccination assortativity							1.19	0.99-1.43	0.07	1.21	1.01-1.46	0.04
Network size							0.99	0.92-1.07	0.889	0.92	0.84-1.02	0.105
Network density							0.98	0.87-1.1	0.717	1.02	0.87-1.2	0.796
Network components (strong)							1.12	1.04-1.21	0.004	1.09	0.95-1.25	0.21
Proportion of vaccinated alters	2.71	2.3-3.2	<.001	2.39	1.95-2.91	<.001	2.73	2.33-3.2	<.001	2.48	2.05-3.01	<.001
Model fit												
Nagelkerke Pseudo R-squared	0.23			0.31			0.243			0.32		
Cox and Snell Pseudo R-squared	0.162			0.217			0.17			0.224		
Tjur Pseudo R-squared	0.177			0.243			0.188			0.251		
Deviance	1320.945			1232.597			1307.436			1221.777		
AIC	1324.945			1258.597			1323.436			1259.777		

5.2 Multicolliniarity

5.2.1 VIF scores for multilevel models

table S25

```
# vif scores for multilevel model 2
vif_values_m2 <- vif(m2)
vif_values_m2 %>% kbl(caption = "VIF scores for multilevel regression model 2 (attributes only)",
                        booktabs = T, linesep = "") %>%
  kable_classic() %>% kable_styling(latex_options = "hold_position", full_width = F)
```

Table S25: VIF scores for multilevel regression model 2 (attributes only)

	GVIF	Df	GVIF^(1/(2*Df))
alter.sex.f	1.029336	1	1.014562
alter.education.mc	1.105691	1	1.051519
alter.single.f	1.041392	1	1.020486
alter.age.mc	1.148775	1	1.071809
ego.sex.f	1.109641	1	1.053395
ego.education.mc	1.299905	1	1.140133
ego.age.mc	1.392157	1	1.179897
ego.employed.f	1.353874	1	1.163561
ego.vaccination.f	1.187148	1	1.089563
ego.media.use.f	1.458873	2	1.099017

table S26

```
# vif scores for multilevel model 3
vif_values_m3 <- vif(m3)
vif_values_m3 <- data.frame(vif_values_m3) %>%
  # rename first column and create second column with degrees of freedom (all 1)
  rename(GVIF = vif_values_m3) %>% mutate(Df = 1)
vif_values_m3 %>% kbl(caption = "VIF scores for multilevel regression model 3 (network only)",
                       booktabs = T, linesep = "") %>%
  kable_classic() %>% kable_styling(latex_options = "hold_position", full_width = F)
```

Table S26: VIF scores for multilevel regression model 3 (network only)

	GVIF	Df
ego.alter.intensity	1.116769	1
alter.betweenness.mc	1.111111	1
vaccination.assortativity.mc	1.002271	1
network_size.mc	1.013521	1
network_density.mc	1.150727	1
network_components.mc	1.128523	1

table S27

```
# vif scores for multilevel model 4
vif_values_m4 <- vif(m4)
vif_values_m4 %>% kbl(caption = "VIF scores for multilevel regression model 4 (full model)",
                      booktabs = T, linesep = "") %>%
  kable_classic() %>% kable_styling(latex_options = "hold_position", full_width = F)
```

Table S27: VIF scores for multilevel regression model 4 (full model)

	GVIF	Df	GVIF^(1/(2*Df))
alter.sex.f	1.034679	1	1.017192
alter.education.mc	1.118067	1	1.057387
alter.single.f	1.055654	1	1.027450
alter.age.mc	1.164632	1	1.079181
ego.sex.f	1.200309	1	1.095586
ego.education.mc	1.394055	1	1.180701
ego.age.mc	1.433352	1	1.197227
ego.employed.f	1.790551	1	1.338115
ego.vaccination.f	1.250737	1	1.118364
ego.media.use.f	1.676267	2	1.137852
ego.alter.intensity	1.140110	1	1.067759
alter.betweenness.mc	1.119799	1	1.058206
vaccination.assortativity.mc	1.014836	1	1.007391
network_size.mc	1.208125	1	1.099147
network_density.mc	1.609461	1	1.268645
network_components.mc	1.196982	1	1.094067

5.2.2 VIF scores for GLM models with clustered errors

table S28

```
# vif scores for GLM model 2
vif_values_m2.glm <- vif(m2.glm$glm_res)
vif_values_m2.glm %>% kbl(caption = "VIF scores for GLM regression model 2 (attributes only)",
                         booktabs = T, linesep = "") %>%
  kable_classic() %>% kable_styling(latex_options = "hold_position", full_width = F)
```

Table S28: VIF scores for GLM regression model 2 (attributes only)

	GVIF	Df	GVIF^(1/(2*Df))
alter.sex.f	1.075138	1	1.036889
alter.education.mc	1.158599	1	1.076382
alter.single.f	1.063027	1	1.031032
alter.age.mc	1.370822	1	1.170821
ego.sex.f	1.139309	1	1.067384
ego.education.mc	1.489295	1	1.220367
ego.age.mc	1.584846	1	1.258907
ego.employed.f	1.359012	1	1.165767
ego.vaccination.f	1.283581	1	1.132952
ego.media.use.f	1.554306	2	1.116565
prop.vacc.alters__ex.alter.mc	1.394908	1	1.181062

table S29

```
# vif scores for GLM model 3
vif_values_m3.glm <- vif(m3.glm$glm_res)
vif_values_m3.glm <- data.frame(vif_values_m3.glm) %>%
  # rename first column and create second column with degrees of freedom (all 1)
  rename(GVIF = vif_values_m3.glm) %>% mutate(Df = 1)
vif_values_m3.glm %>% kbl(caption = "VIF scores for GLM regression model 3 (network only)",
                         booktabs = T, linesep = "") %>%
  kable_classic() %>% kable_styling(latex_options = "hold_position", full_width = F)
```

Table S29: VIF scores for GLM regression model 3 (network only)

	GVIF	Df
ego.alter.intensity	1.118972	1
alter.betweenness.mc	1.084884	1
vaccination.assortativity.mc	1.012941	1
network__size.mc	1.045412	1
network__density.mc	1.177601	1
network__components.mc	1.103353	1
prop.vacc.alters__ex.alter.mc	1.028133	1

table S30

```
# vif scores for GLM model 4
vif_values_m4.glm <- vif(m4.glm$glm_res)
vif_values_m4.glm %>% kbl(caption = "VIF scores for GLM regression model 4 (full model)",
                         booktabs = T, linesep = "") %>%
  kable_classic() %>% kable_styling(latex_options = "hold_position", full_width = F)
```

Table S30: VIF scores for GLM regression model 4 (full model)

	GVIF	Df	GVIF^(1/(2*Df))
alter.sex.f	1.084204	1	1.041251
alter.education.mc	1.181155	1	1.086810
alter.single.f	1.102803	1	1.050145
alter.age.mc	1.396110	1	1.181571
ego.sex.f	1.194083	1	1.092741
ego.education.mc	1.606034	1	1.267294
ego.age.mc	1.679130	1	1.295813
ego.employed.f	1.800862	1	1.341962
ego.vaccination.f	1.400991	1	1.183634
ego.media.use.f	1.882543	2	1.171349
ego.alter.intensity	1.153050	1	1.073802
alter.betweenness.mc	1.107452	1	1.052355
vaccination.assortativity.mc	1.048444	1	1.023936
network__size.mc	1.315809	1	1.147087
network__density.mc	1.637790	1	1.279762
network__components.mc	1.199886	1	1.095393
prop.vacc.alters__ex.alter.mc	1.526635	1	1.235571

5.3 Predictive performance

5.3.1 ROC curve for multilevel models

figure S2

```r
# roc_curve multilevel model 1 (m1)
data_m1 <- getData(m1)
predicted_probs_m1 <- predict(m1, type = "response")
roc_curve <- roc(data_m1$alter.vaccination.f, predicted_probs_m1)
auc_value <- auc(roc_curve)
plot(roc_curve, main = sprintf("ROC Curve (AUC = %.2f)", auc_value))
```

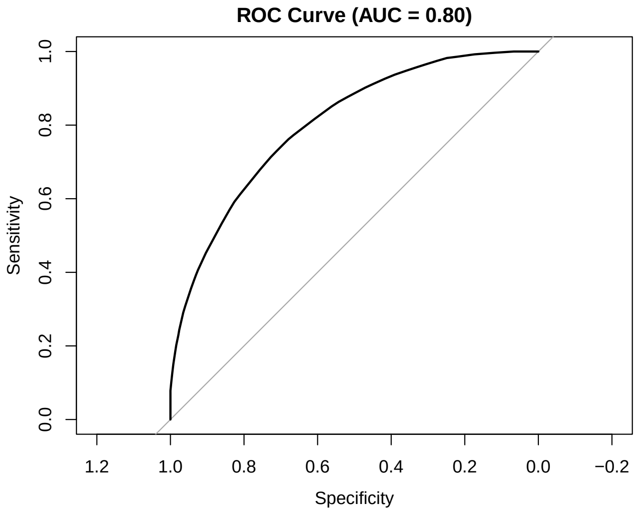

Figure S2: ROC curve for multilevel model 1 (null model)

figure S3

```r
# roc_curve multilevel model 2 (m2)
data_m2 <- getData(m2)
predicted_probs_m2 <- predict(m2, type = "response")
roc_curve <- roc(data_m2$alter.vaccination.f, predicted_probs_m2)
auc_value <- auc(roc_curve)
plot(roc_curve, main = sprintf("ROC Curve (AUC = %.2f)", auc_value))
```

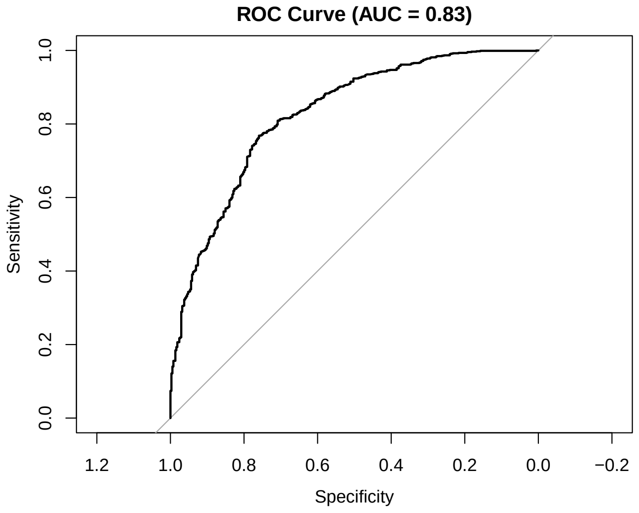

Figure S3: ROC curve for multilevel model 2 (attributes only)

figure S4

```r
# roc_curve multilevel model 3 (m3)
data_m3 <- getData(m3)
predicted_probs_m3 <- predict(m3, type = "response")
roc_curve <- roc(data_m3$alter.vaccination.f, predicted_probs_m3)
auc_value <- auc(roc_curve)
plot(roc_curve, main = sprintf("ROC Curve (AUC = %.2f)", auc_value))
```

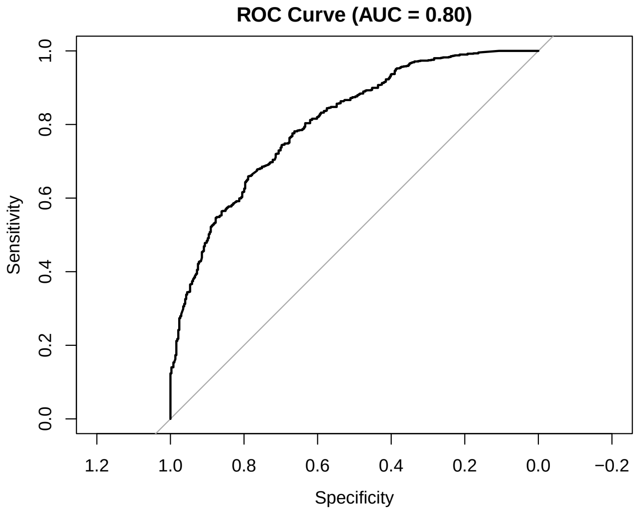

Figure S4: ROC curve for multilevel model 3 (network only)

figure S5

```
# roc_curve multilevel model 4 (m4)
data_m4 <- getData(m4)
predicted_probs_m4 <- predict(m4, type = "response")
roc_curve <- roc(data_m4$alter.vaccination.f, predicted_probs_m4)
auc_value <- auc(roc_curve)
plot(roc_curve, main = sprintf("ROC Curve (AUC = %.2f)", auc_value))
```

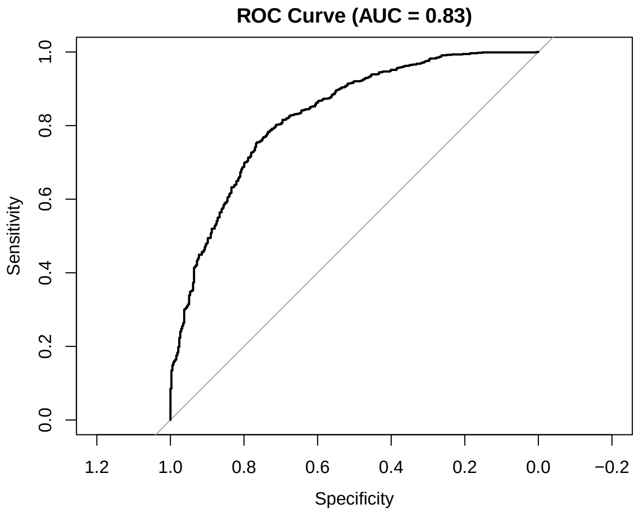

Figure S5: ROC curve for multilevel model 4 (full model)

5.3.1 ROC curve for GLM models with clustered errors

figure S6

```
# roc_curve GLM model 1 (m1.glm)
data_m1.glm <- m1.glm$glm_res$data
predicted_probs_m1.glm <- predict(m1.glm$glm_res, type = "response")
roc_curve <- roc(data_m1.glm$alter.vaccination.f, predicted_probs_m1.glm)
auc_value <- auc(roc_curve)
plot(roc_curve, main = sprintf("ROC Curve (AUC = %.2f)", auc_value))
```

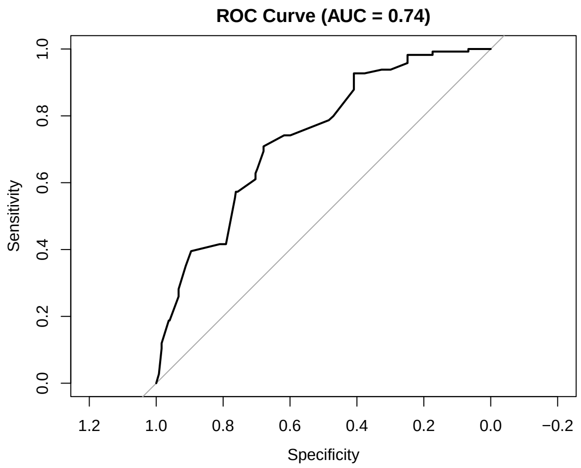

Figure S6: ROC curve for GLM model 1 (null model)

figure S7

```r
# roc_curve GLM model 2 (m2.glm)
data_m2.glm <- m2.glm$glm_res$data
predicted_probs_m2.glm <- predict(m2.glm$glm_res, type = "response")
roc_curve <- roc(data_m2.glm$alter.vaccination.f, predicted_probs_m2.glm)
auc_value <- auc(roc_curve)
plot(roc_curve, main = sprintf("ROC Curve (AUC = %.2f)", auc_value))
```

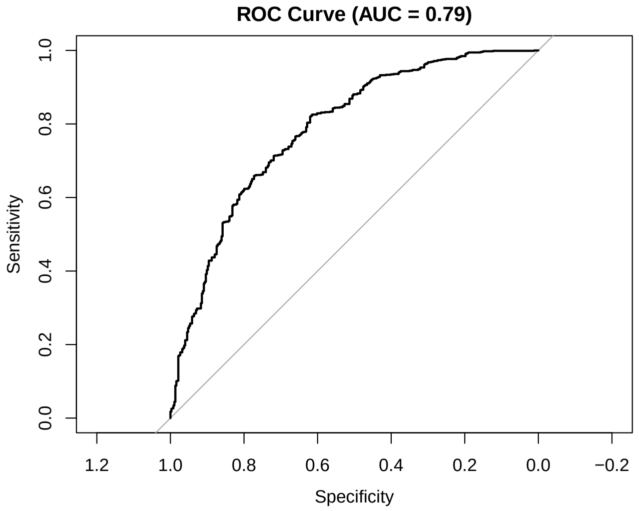

Figure S7: ROC curve for GLM model 2 (attributes only)

figure S8

```r
# roc_curve GLM model 3 (m3.glm)
data_m3.glm <- m3.glm$glm_res$data
predicted_probs_m3.glm <- predict(m3.glm$glm_res, type = "response")
roc_curve <- roc(data_m3.glm$alter.vaccination.f, predicted_probs_m3.glm)
auc_value <- auc(roc_curve)
plot(roc_curve, main = sprintf("ROC Curve (AUC = %.2f)", auc_value))
```

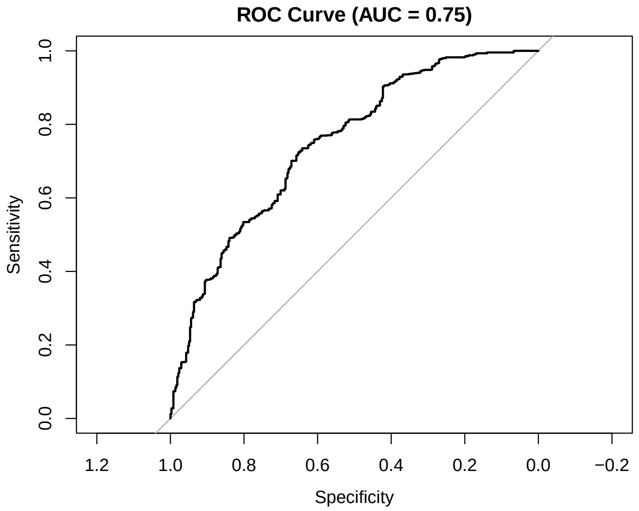

Figure S8: ROC curve for GLM model 3 (network only)

figure S9

```r
# roc_curve GLM model 4 (m4.glm)
data_m4.glm <- m4.glm$glm_res$data
predicted_probs_m4.glm <- predict(m4.glm$glm_res, type = "response")
roc_curve <- roc(data_m4.glm$alter.vaccination.f, predicted_probs_m4.glm)
auc_value <- auc(roc_curve)
plot(roc_curve, main = sprintf("ROC Curve (AUC = %.2f)", auc_value))
```

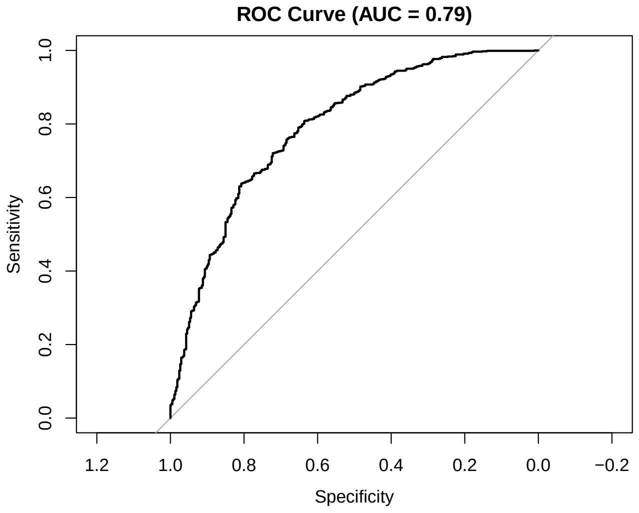

Figure S9: ROC curve for GLM model 4 (full model)

5.4 Residual plots

5.4.1 Residual plots for multilevel models

figure S10

```
# multilevel model 1 (m1)
fitted_values_m1 <- fitted(m1)
residuals_m1 <- resid(m1)
data_frame <- data.frame(Fitted = fitted_values_m1, Residuals = residuals_m1)
ggplot(data_frame, aes(x = Fitted, y = Residuals)) + geom_point() +geom_hline(yintercept = 0, linetype = "dashed") +
  theme_minimal() + xlab("Fitted Values") + ylab("Residuals")
```

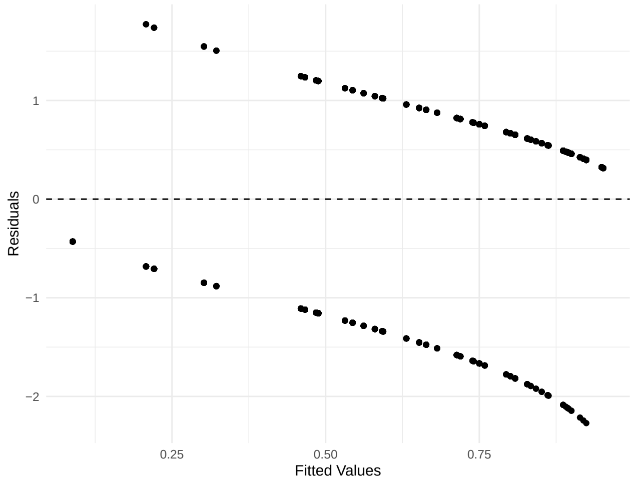

Figure S10: Residuals plot for multilevel model 1 (null model)

figure S11

```
# multilevel model 2 (m2)
fitted_values_m2 <- fitted(m2)
residuals_m2 <- resid(m2)
data_frame <- data.frame(Fitted = fitted_values_m2, Residuals = residuals_m2)
ggplot(data_frame, aes(x = Fitted, y = Residuals)) + geom_point() + geom_hline(yintercept = 0, linetype = "dashed") +
  theme_minimal() + xlab("Fitted Values") + ylab("Residuals")
```

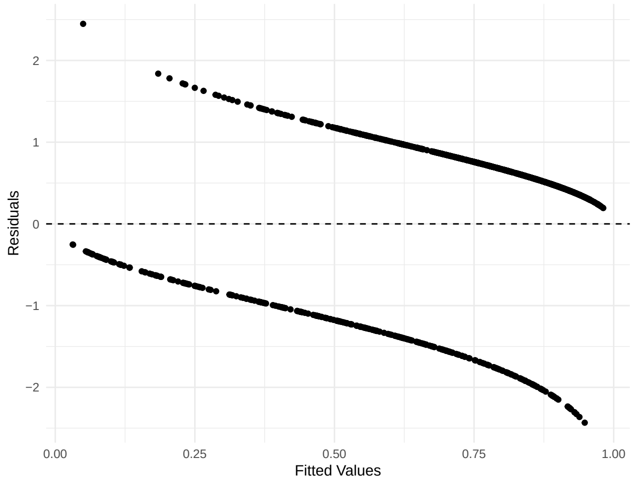

Figure S11: Residuals plot for multilevel model 2 (attributes only)

figure S12

```
# multilevel model 3 (m3)
fitted_values_m3 <- fitted(m3)
residuals_m3 <- resid(m3)
data_frame <- data.frame(Fitted = fitted_values_m3, Residuals = residuals_m3)
ggplot(data_frame, aes(x = Fitted, y = Residuals)) + geom_point() + geom_hline(yintercept = 0, linetype = "dashed") +
  theme_minimal() + xlab("Fitted Values") + ylab("Residuals")
```

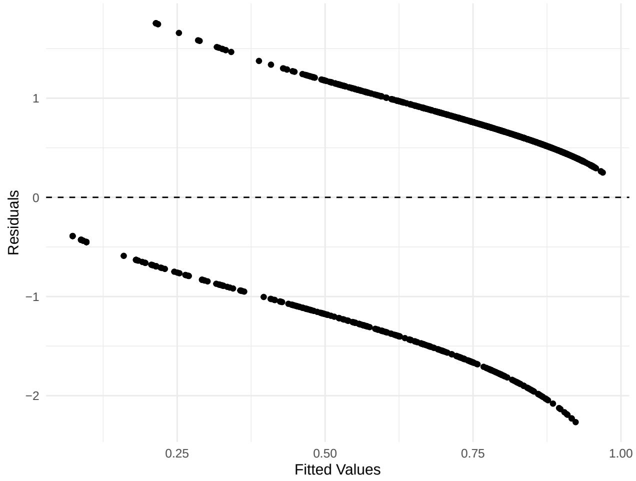

Figure S12: Residuals plot for multilevel model 3 (network only)

figure S13

```
# multilevel model 4 (m4)
fitted_values_m4 <- fitted(m4)
residuals_m4 <- resid(m4)
data_frame <- data.frame(Fitted = fitted_values_m4, Residuals = residuals_m4)
ggplot(data_frame, aes(x = Fitted, y = Residuals)) + geom_point() + geom_hline(yintercept = 0, linetype = "dashed") +
  theme_minimal() + xlab("Fitted Values") + ylab("Residuals")
```

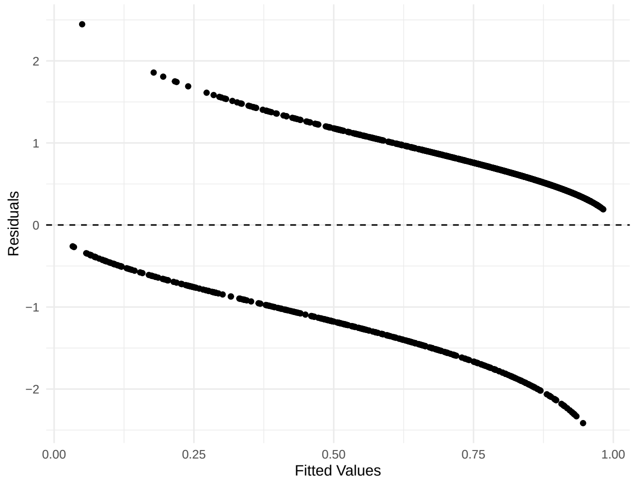

Figure S13: Residuals plot for multilevel model 4 (full model)

5.4.2 Residual plots for GLM models with clustered errors

figure S14

```
# GLM model 1 (m1.glm)
fitted_values_m1.glm <- fitted(m1.glm$glm_res)
residuals_m1.glm <- resid(m1.glm$glm_res)
data_frame <- data.frame(Fitted = fitted_values_m1.glm, Residuals = residuals_m1.glm)
ggplot(data_frame, aes(x = Fitted, y = Residuals)) + geom_point() + geom_hline(yintercept = 0, linetype = "dashed") +
  theme_minimal() + xlab("Fitted Values") + ylab("Residuals")
```

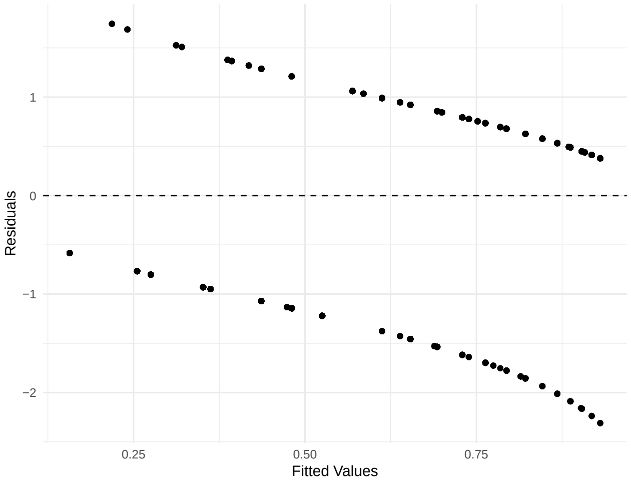

Figure S14: Residuals plot for GLM model 1 (null model)

figure S15

```
# GLM model 2 (m2.glm)
fitted_values_m2.glm <- fitted(m2.glm$glm_res)
residuals_m2.glm <- resid(m2.glm$glm_res)
data_frame <- data.frame(Fitted = fitted_values_m2.glm, Residuals = residuals_m2.glm)
ggplot(data_frame, aes(x = Fitted, y = Residuals)) + geom_point() + geom_hline(yintercept = 0, linetype = "dashed") +
  theme_minimal() + xlab("Fitted Values") + ylab("Residuals")
```

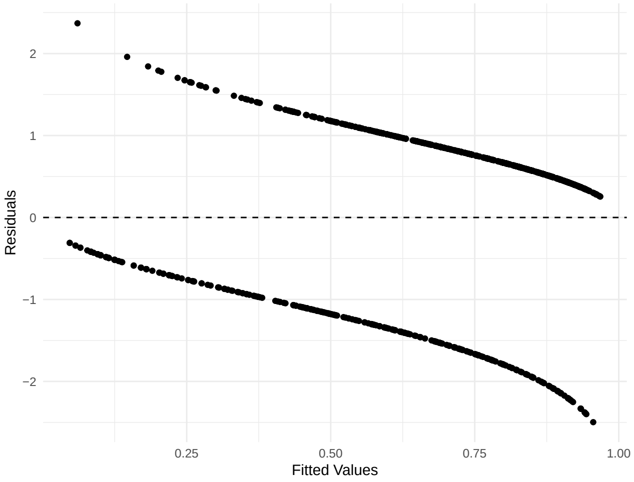

Figure S15: Residuals plot for GLM model 2 (attributes only)

figure S16

```r
# GLM model 3 (m3.glm)
fitted_values_m3.glm <- fitted(m3.glm$glm_res)
residuals_m3.glm <- resid(m3.glm$glm_res)
data_frame <- data.frame(Fitted = fitted_values_m3.glm, Residuals = residuals_m3.glm)
ggplot(data_frame, aes(x = Fitted, y = Residuals)) + geom_point() + geom_hline(yintercept = 0, linetype = "dashed") +
  theme_minimal() + xlab("Fitted Values") + ylab("Residuals")
```

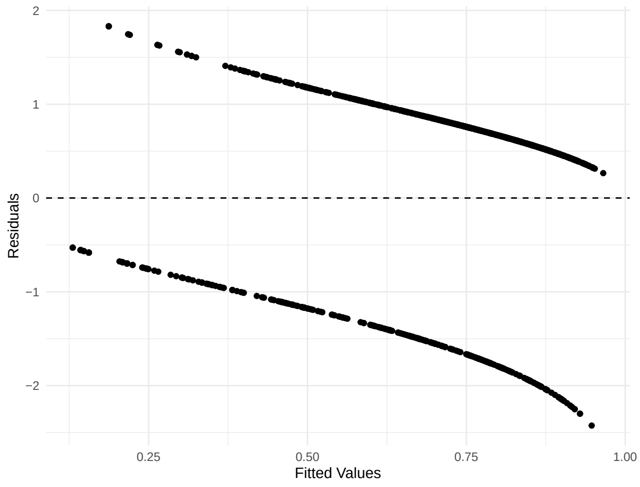

Figure S16: Residuals plot for GLM model 3 (network only)

figure S17

```r
# GLM model 4 (m4.glm)
fitted_values_m4.glm <- fitted(m4.glm$glm_res)
residuals_m4.glm <- resid(m4.glm$glm_res)
data_frame <- data.frame(Fitted = fitted_values_m4.glm, Residuals = residuals_m4.glm)
ggplot(data_frame, aes(x = Fitted, y = Residuals)) + geom_point() + geom_hline(yintercept = 0, linetype = "dashed") +
  theme_minimal() + xlab("Fitted Values") + ylab("Residuals")
```

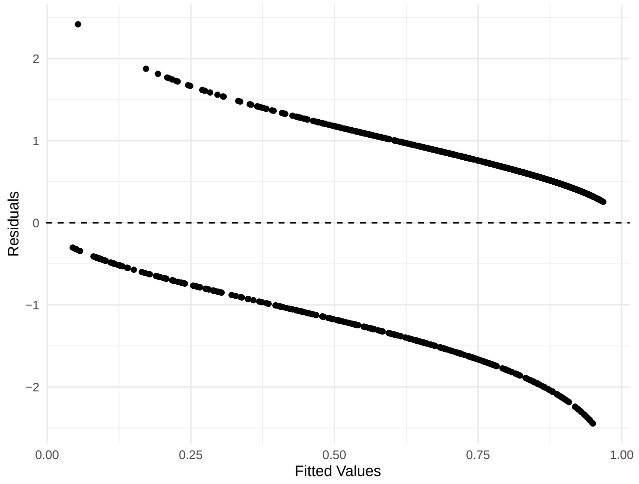

Figure S17: Residuals plot for GLM model 4 (full model)

5.5 Overdispersion estimation

5.5.1 Residual plots for multilevel models

table S31

```
# multilevel model 1 (m1)
print(DHARMa::testDispersion(m1, plot = F))
```

```
DHARMa nonparametric dispersion test via sd of residuals fitted vs.
simulated
```

data: simulationOutput dispersion = 0.99245, p-value = 0.864 alternative hypothesis: two.sided

figure S18

```
# multilevel model 1 (m1)
plot(DHARMa::simulateResiduals(m1))
```

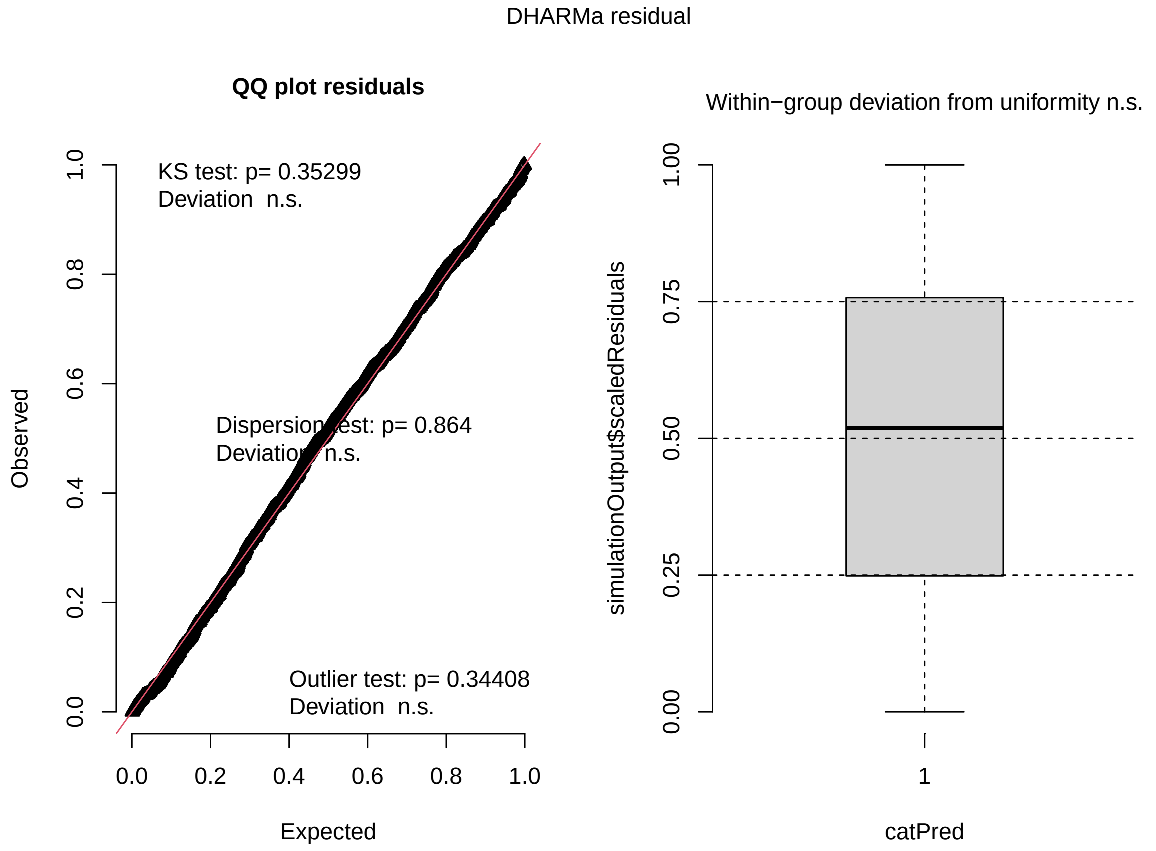

Figure S18: Overdispersion plots for multilevel model 1 (null model)

table S32

```r
# multilevel model 2 (m2)
print(DHARMa::testDispersion(m2, plot = F))
```

```
DHARMa nonparametric dispersion test via sd of residuals fitted vs.
simulated
```

data: simulationOutput dispersion = 0.9763, p-value = 0.696 alternative hypothesis: two.sided

figure S19

```r
# multilevel model 2 (m2)
plot(DHARMa::simulateResiduals(m2))
```

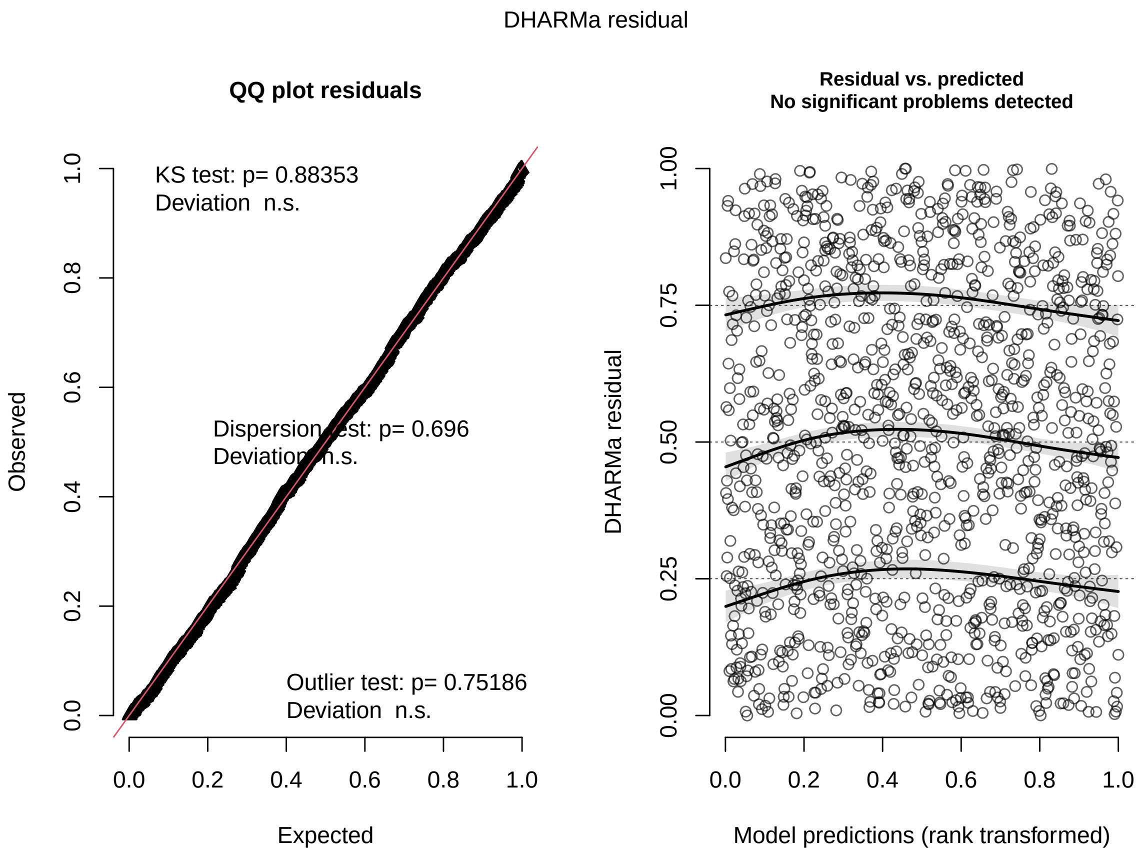

Figure S19: Overdispersion plots for multilevel model 2 (attributes only)

table S33

```r
# multilevel model 3 (m3)
print(DHARMa::testDispersion(m3, plot = F))
```

```
DHARMa nonparametric dispersion test via sd of residuals fitted vs.
simulated
```

data: simulationOutput dispersion = 0.98585, p-value = 0.816 alternative hypothesis: two.sided

figure S20

```r
# multilevel model 3 (m3)
plot(DHARMa::simulateResiduals(m3))
```

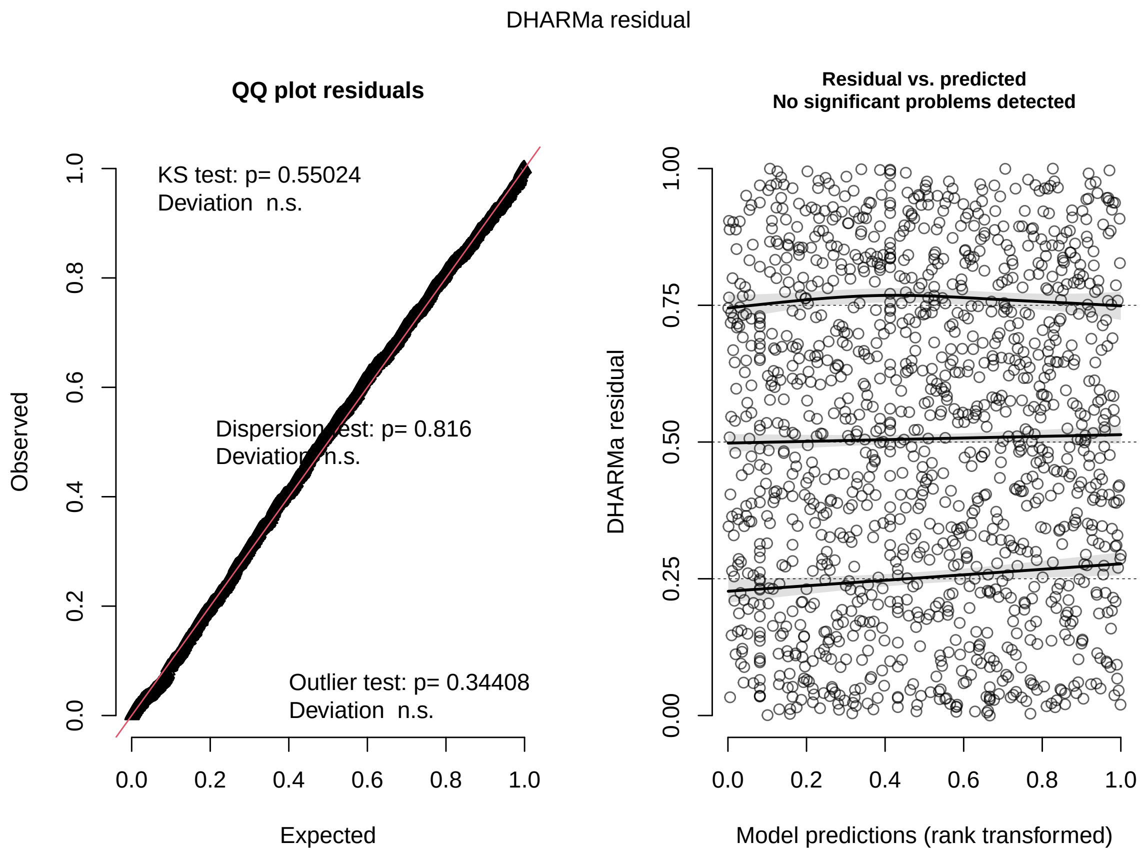

Figure S20: Overdispersion plots for multilevel model 3 (network only)

table S34

```r
# multilevel model 4 (m4)
print(DHARMa::testDispersion(m4, plot = F))
```

```
DHARMa nonparametric dispersion test via sd of residuals fitted vs.
simulated
```

data: simulationOutput dispersion = 0.96819, p-value = 0.6 alternative hypothesis: two.sided

figure S21

```r
# multilevel model 4 (m4)
plot(DHARMa::simulateResiduals(m4))
```

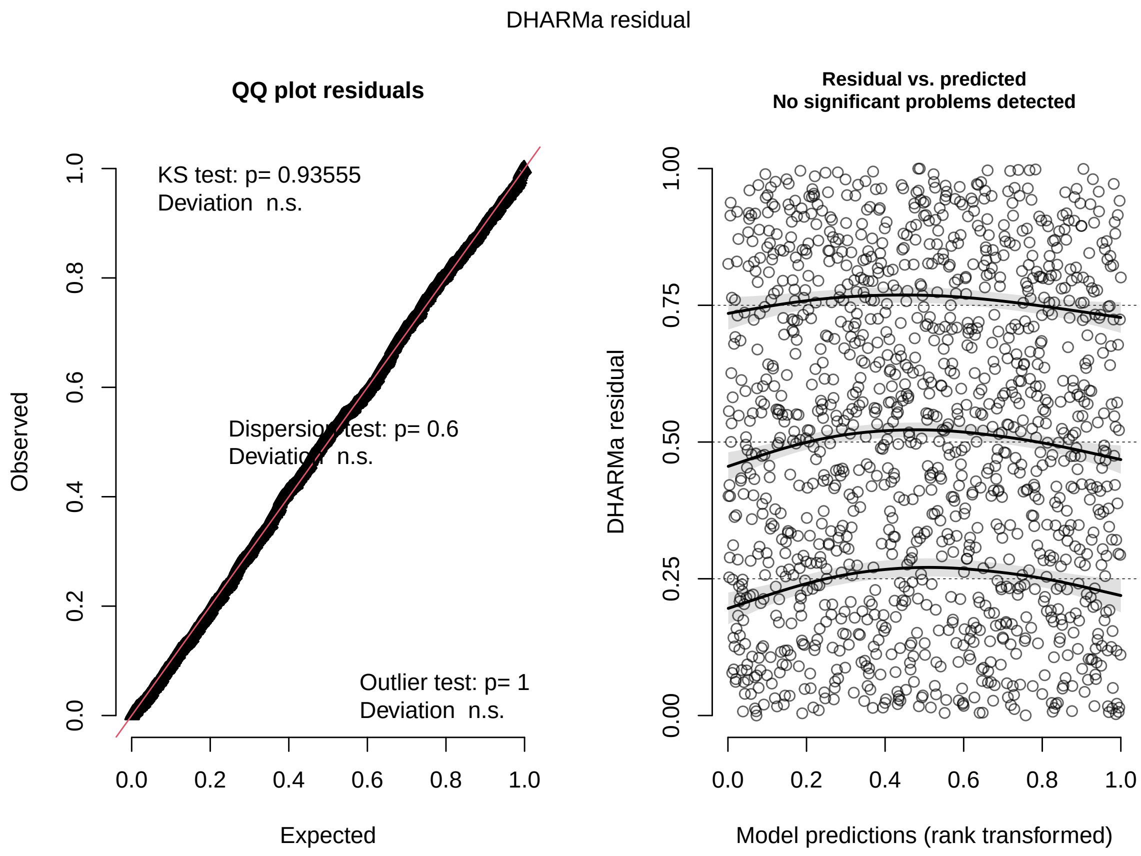

Figure S21: Overdispersion plots for multilevel model 4 (full model)

5.5.2 Residual plots for GLM models with clustered errors

table S35

```
# GLM model 1 (m1.glm)
print(DHARMa::testDispersion(m1.glm$glm_res, plot = F))
```

```
DHARMa nonparametric dispersion test via sd of residuals fitted vs.
simulated
```

data: simulationOutput dispersion = 0.99743, p-value = 0.968 alternative hypothesis: two.sided

figure S22

```
# GLM model 1 (m1.glm)
plot(DHARMa::simulateResiduals(m1.glm$glm_res))
```

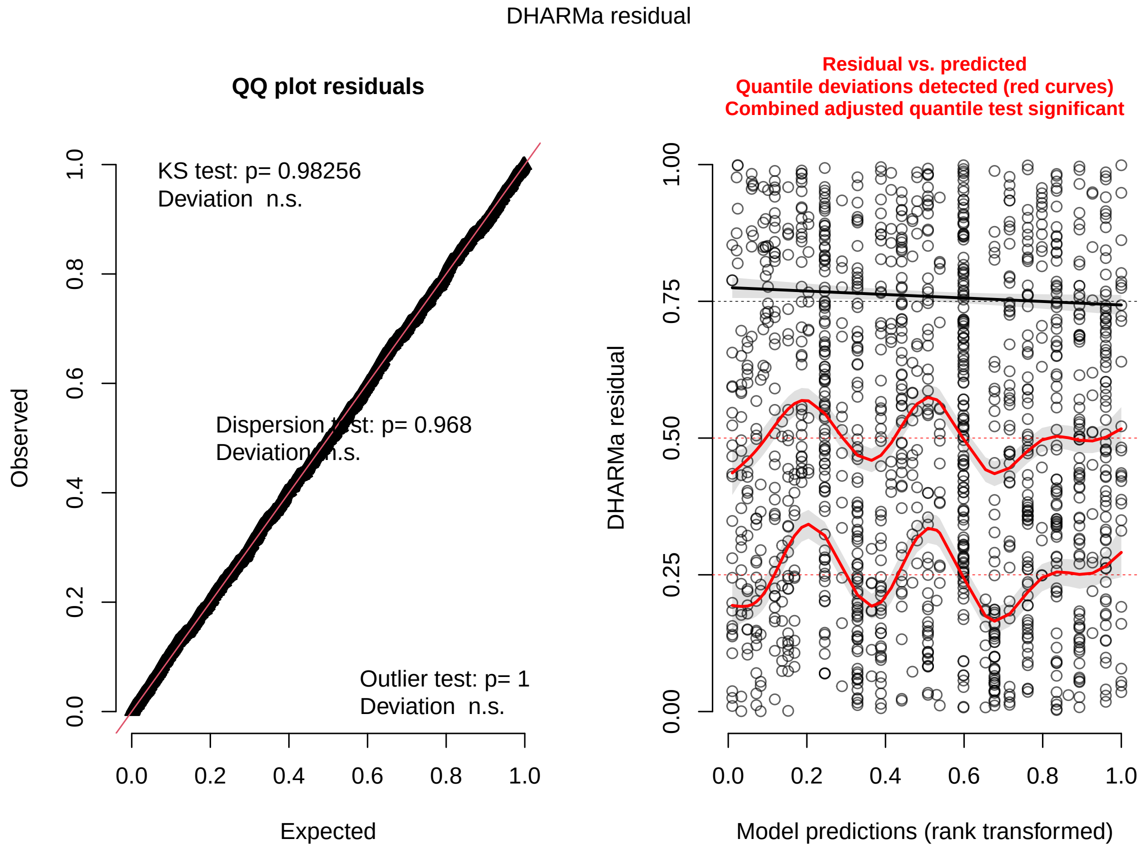

Figure S22: Overdispersion plots for GLM model 1 (null model)

table S36

```r
# GLM model 2 (m2.glm)
print(DHARMa::testDispersion(m2.glm$glm_res, plot = F))
```

```
DHARMa nonparametric dispersion test via sd of residuals fitted vs.
simulated
```

data: simulationOutput dispersion = 1.0007, p-value = 1 alternative hypothesis: two.sided

figure S23

```r
# GLM model 2 (m2.glm)
plot(DHARMa::simulateResiduals(m2.glm$glm_res))
```

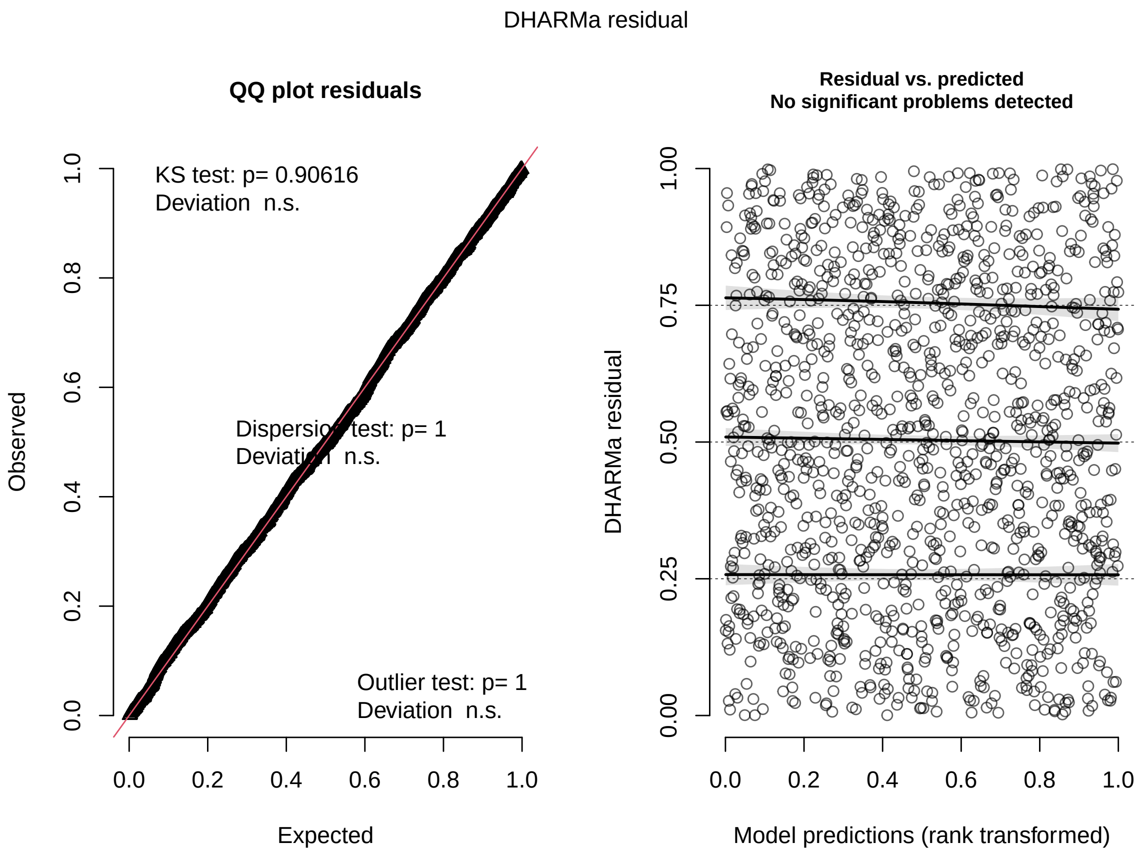

Figure S23: Overdispersion plots for GLM model 2 (attributes only)

table S37

```r
# GLM model 3 (m3.glm)
print(DHARMa::testDispersion(m3.glm$glm_res, plot = F))
```

```
DHARMa nonparametric dispersion test via sd of residuals fitted vs.
simulated
```

data: simulationOutput dispersion = 0.99767, p-value = 1 alternative hypothesis: two.sided

figure S24

```r
# GLM model 3 (m3.glm)
plot(DHARMa::simulateResiduals(m3.glm$glm_res))
```

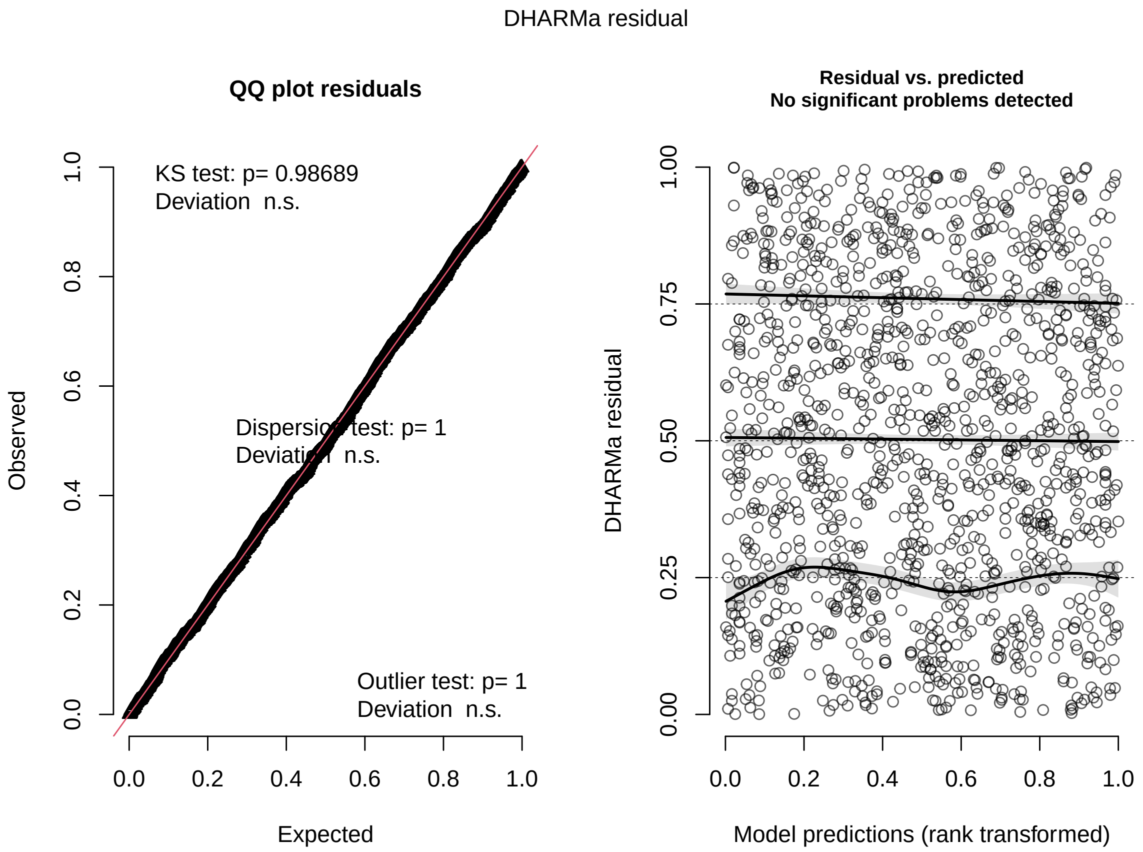

Figure S24: Overdispersion plots for GLM model 3 (network only)

table S38

```r
# GLM model 4 (m4.glm)
print(DHARMa::testDispersion(m4.glm$glm_res, plot = F))
```

```
DHARMa nonparametric dispersion test via sd of residuals fitted vs.
simulated
```

data: simulationOutput dispersion = 0.99897, p-value = 0.936 alternative hypothesis: two.sided

figure S25

```r
# GLM model 4 (m4.glm)
plot(DHARMa::simulateResiduals(m4.glm$glm_res))
```

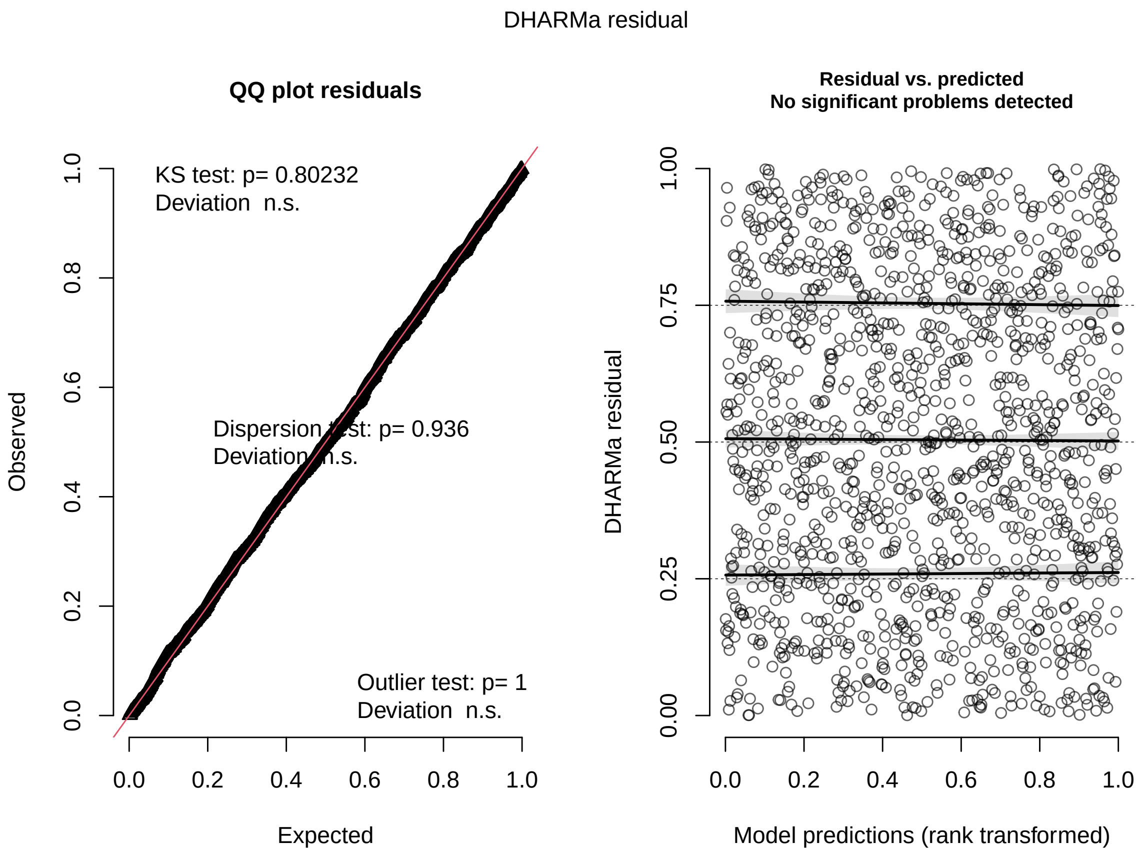

Figure S25: Overdispersion plots for GLM model 4 (full model)

5.6 Plots of the residuals for random effects in multilevel models

figure S26

```
# model 1 (m1)
ranef_resid <- ranef(m1, condVar = TRUE)
plot(ranef_resid, which = "random_effect")
```

$networkCanvasEgoUUID

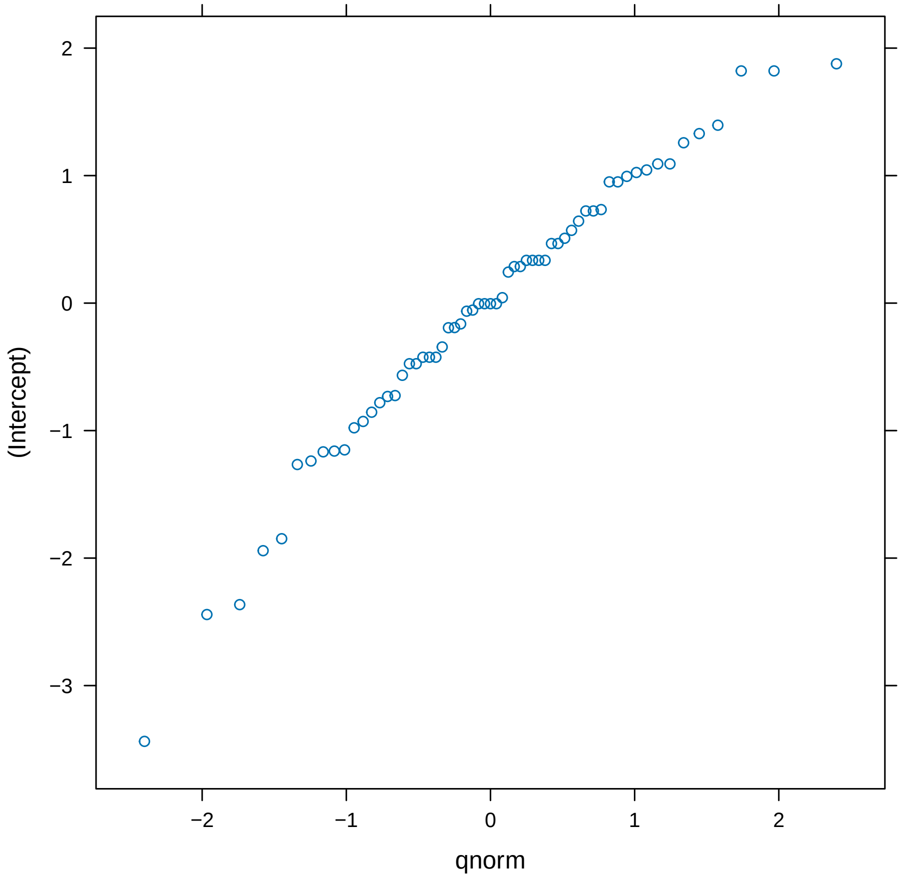

Figure S26: Residuals for random effects plots for multilevel model 1 (null model)

figure S27

```r
# model 2 (m2)
ranef_resid <- ranef(m2, condVar = TRUE)
plot(ranef_resid, which = "random_effect")
```

$networkCanvasEgoUUID

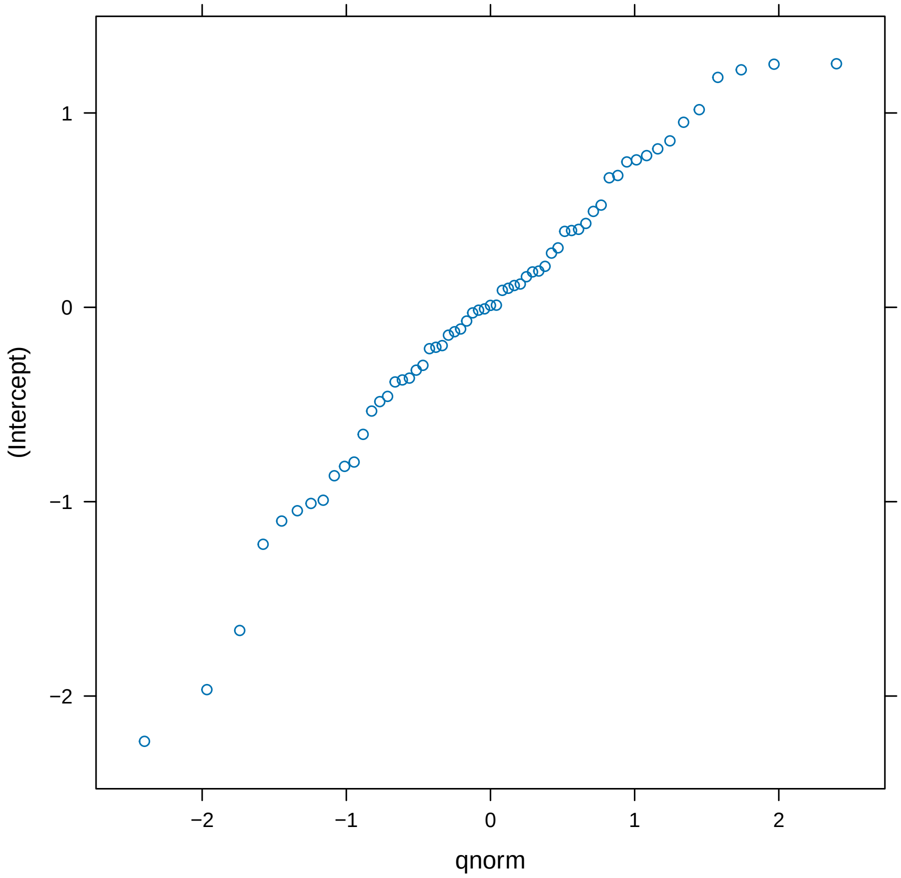

Figure S27: Residuals for random effects plots for multilevel model 2 (attributes only)

figure S28

```
# model 3 (m3)
ranef_resid <- ranef(m3, condVar = TRUE)
plot(ranef_resid, which = "random_effect")
```

$networkCanvasEgoUUID

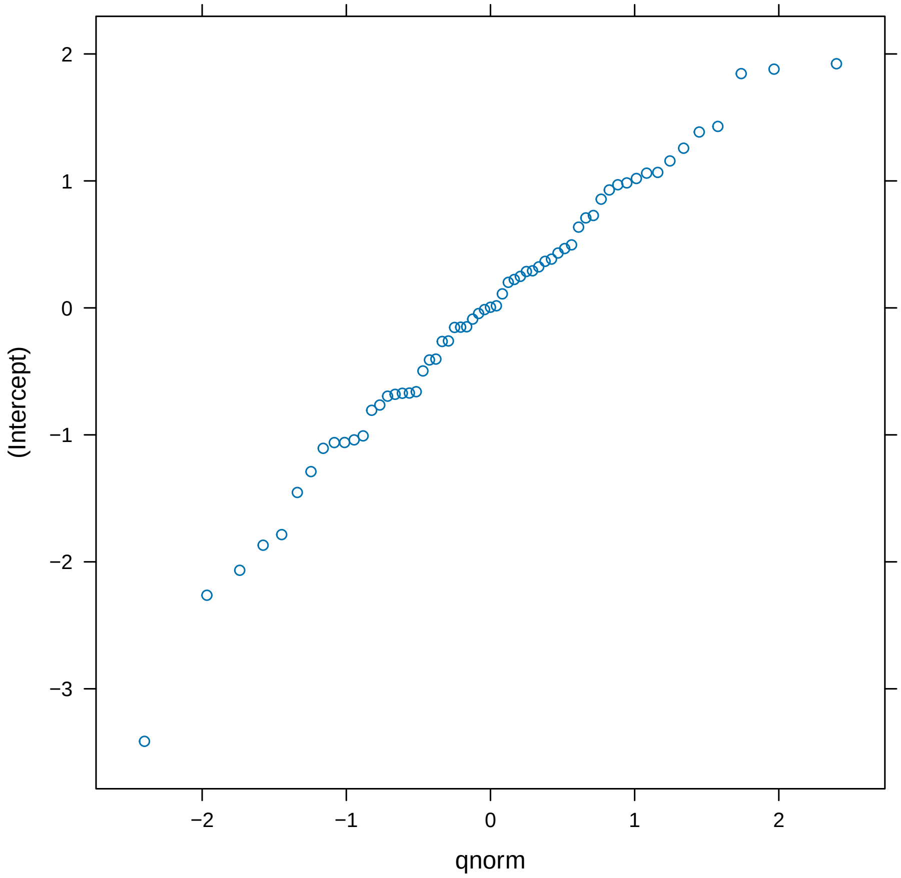

Figure S28: Residuals for random effects plots for multilevel model 3 (network only)

figure S29

```
# model 4 (m4)
ranef_resid <- ranef(m4, condVar = TRUE)
plot(ranef_resid, which = "random_effect")
```

$networkCanvasEgoUUID

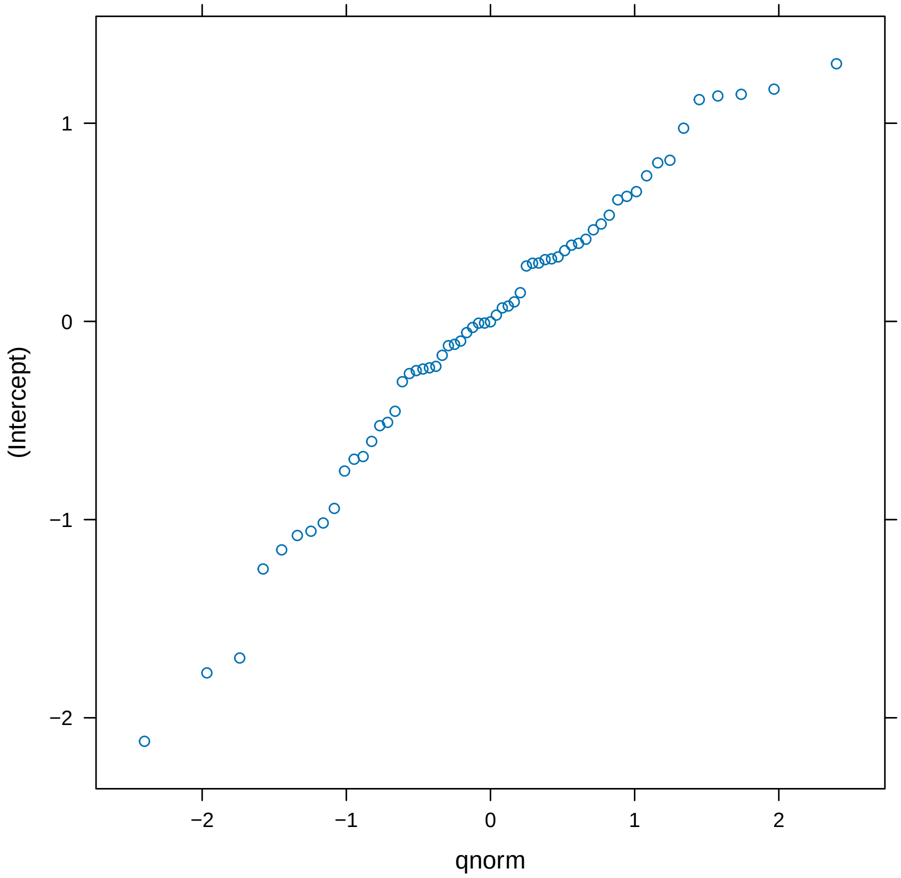

Figure S29: Residuals for random effects plots for multilevel model 4 (full model)

6. Miscellaneous (Eurostat data)

We mention that, as these data are dynamically downloaded using the `get_eurostat()` function from the `eurostat` package, slight changes in percentages might appear, when comparing the values from the article with those from the subsequent figures.

```
# Get labels for EU countries

eu_c.labels <- eurostat::eu_countries %>% rename(geo = code, geo.name = name) %>% dplyr::select(geo, geo.name)
```

6.1 Eurostat data from dataset "Individuals' level of digital skills (from 2021 onwards)"

Online data code: `isoc_sk_dskl_i21`

link: `https://ec.europa.eu/eurostat/databrowser/view/isoc_sk_dskl_i21/default/table?lang=en`

```
# Download data
eurostat_data1 <- get_eurostat("isoc_sk_dskl_i21")

# Create labels for table indicators
eu_i.labels <- data.frame(
  indic_is = c(
    "I_DSK2_BAB",
    "I_DSK2_X"
  ),
  indic_is.label = c(
    "Individuals with basic or above basic overall digital skills (all five component indicators are at basic or above basic
    ↪  level)",
    "Individuals with no overall digital skills"
  )
)

# Subset data
eurostat_data1 <- eurostat_data1 %>%
  # filter data for 2023
  filter(str_sub(TIME_PERIOD, 1, 4) == "2023") %>%
  # select all types of respondents
  filter(ind_type == "IND_TOTAL") %>%
  # select respondents who used the internet in the last 3 months
  filter(unit == "PC_IND_IU3") %>%
  # filter data for countries in the EU (also keep the EU mean)
  filter(geo %in% eu_c.labels$geo | geo == "EU27_2020") %>%
  # rename EU27 label
  mutate(geo = ifelse(geo == "EU27_2020", "EU27 2020", geo)) %>%
  # select only needed columns
  dplyr::select(geo, indic_is, values) %>%
  # select only needed indicators
  filter(indic_is == "I_DSK2_BAB" | indic_is == "I_DSK2_X") %>%
  # attach indicator labels
  left_join(eu_i.labels) %>% relocate(indic_is.label, .after = indic_is) %>%
  # attach country names
  left_join(eu_c.labels) %>% relocate(geo.name, .after = geo)
```

figure S30

```
#
I_DSK2_BAB <- eurostat_data1 %>%
  filter(indic_is == "I_DSK2_BAB")
#
I_DSK2_BAB %>%
  ggplot() +
  aes(x = reorder(geo, values), y = values) +
  geom_col(fill = ifelse(I_DSK2_BAB$geo == "RO", "red",
                ifelse(I_DSK2_BAB$geo ==  "EU27 2020", "orange", "#0C4C8A"))) +
  labs(
    # title = "Individuals with basic or above basic overall digital skills \n(all five component indicators are at basic or
    ↪  above basic level)",
    subtitle = "Eurostat: Individuals' level of digital skills (from 2021 onwards) (code: isoc_sk_dskl_i21)",
    x = "Country",
    y = "% of individuals who used the internet \nin the last 3 months (2023 data)"
  ) +
```

```
  scale_y_continuous(limits=c(0, 100)) +
  ggthemes::theme_pander() +
  theme(
    plot.title = element_text(size = 20),
    plot.subtitle = element_text(size = 9, colour = "gray40", face = "bold"),
    axis.title.y = element_text(face = "bold",
                                margin = margin(t = 0, r = 20, b = 0, l = 10),
                                size = 15),
    axis.title.x = element_text(face = "bold",
                                margin = margin(t = 20, r = 20, b = 0, l = 0),
                                size = 15),
    axis.text.x = element_text(face = "bold", size = 10, angle = 90, colour = "gray40"),
    axis.text.y = element_text(face = "bold", size = 10)
  )  +
  geom_text(aes(label = values),
              size = 3.8,
              vjust = 0.5, nudge_x = 0, hjust = -0.5,
              color = "black",
              angle = 90,
              fontface = "bold")
```

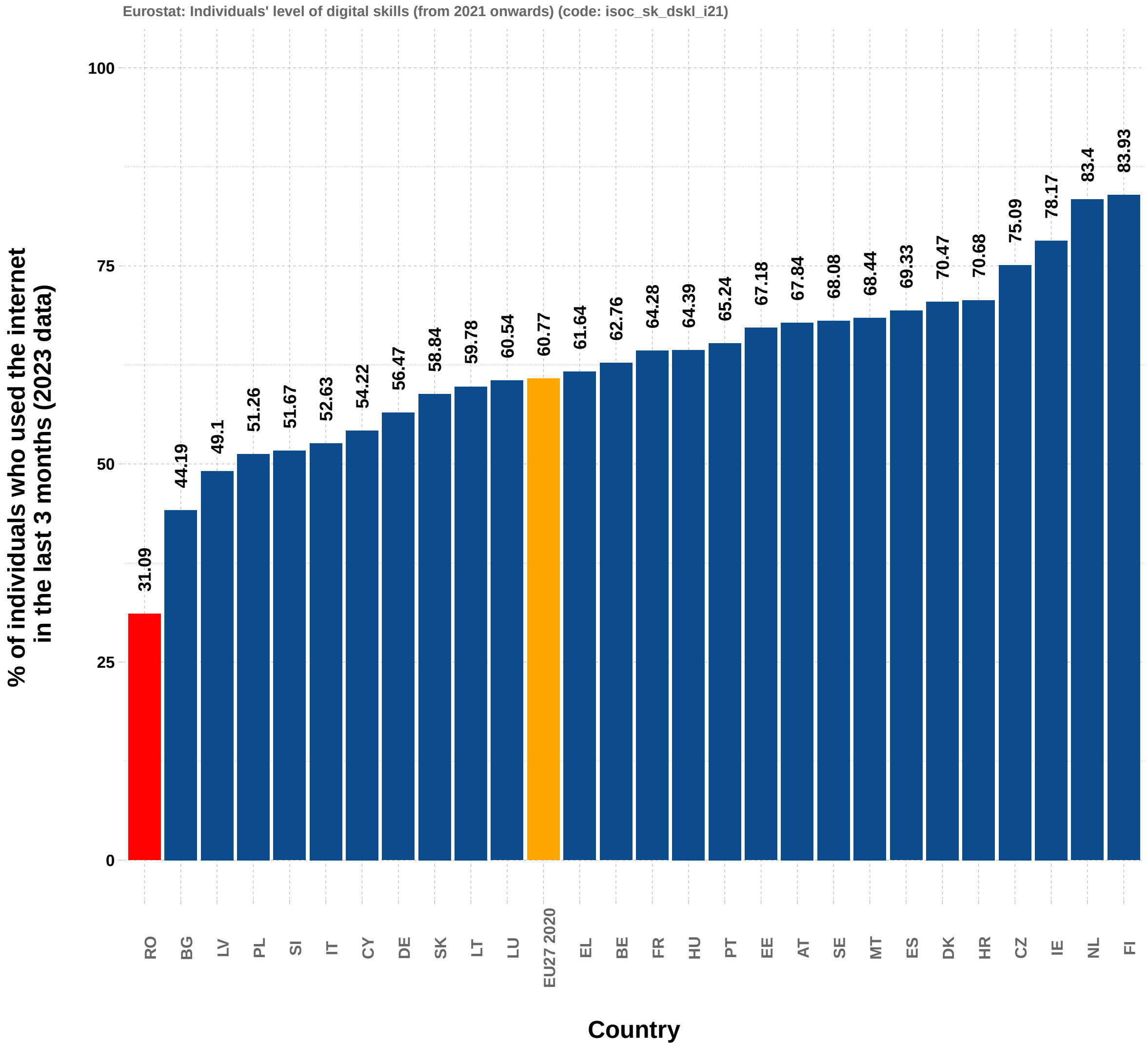

Figure S30: Individuals with basic or above basic overall digital skills (all five component indicators are at basic or above basic level)

figure S31

```r
#
I_DSK2_X <- eurostat_data1 %>%
  filter(indic_is == "I_DSK2_X")
#
I_DSK2_X %>%
  ggplot() +
  aes(x = reorder(geo, values), y = values) +
  geom_col(fill = ifelse(I_DSK2_X$geo == "RO", "red", ifelse(I_DSK2_X$geo ==  "EU27 2020", "orange", "#0C4C8A"))) +
  labs(
    # title = "Individuals with no overall digital skills",
    subtitle = "Eurostat: Individuals' level of digital skills (from 2021 onwards) (code: isoc_sk_dskl_i21)",
    x = "Country",
    y = "% of individuals who used the internet \nin the last 3 months (2023 data)"
  ) + scale_y_continuous(limits=c(0, 20)) +
  ggthemes::theme_pander() +
  theme(plot.title = element_text(size = 20),
    plot.subtitle = element_text(size = 9, colour = "gray40", face = "bold"),
    axis.title.y = element_text(face = "bold", margin = margin(t = 0, r = 20, b = 0, l = 10),size = 15),
    axis.title.x = element_text(face = "bold", margin = margin(t = 20, r = 20, b = 0, l = 0),size = 15),
    axis.text.x = element_text(face = "bold", size = 10, angle = 90, colour = "gray40"),
    axis.text.y = element_text(face = "bold", size = 10)
  )  +
  geom_text(aes(label = values), size = 3.8, vjust = 0.5, nudge_x = 0, hjust = -0.5, color = "black",
              angle = 90, fontface = "bold")
```

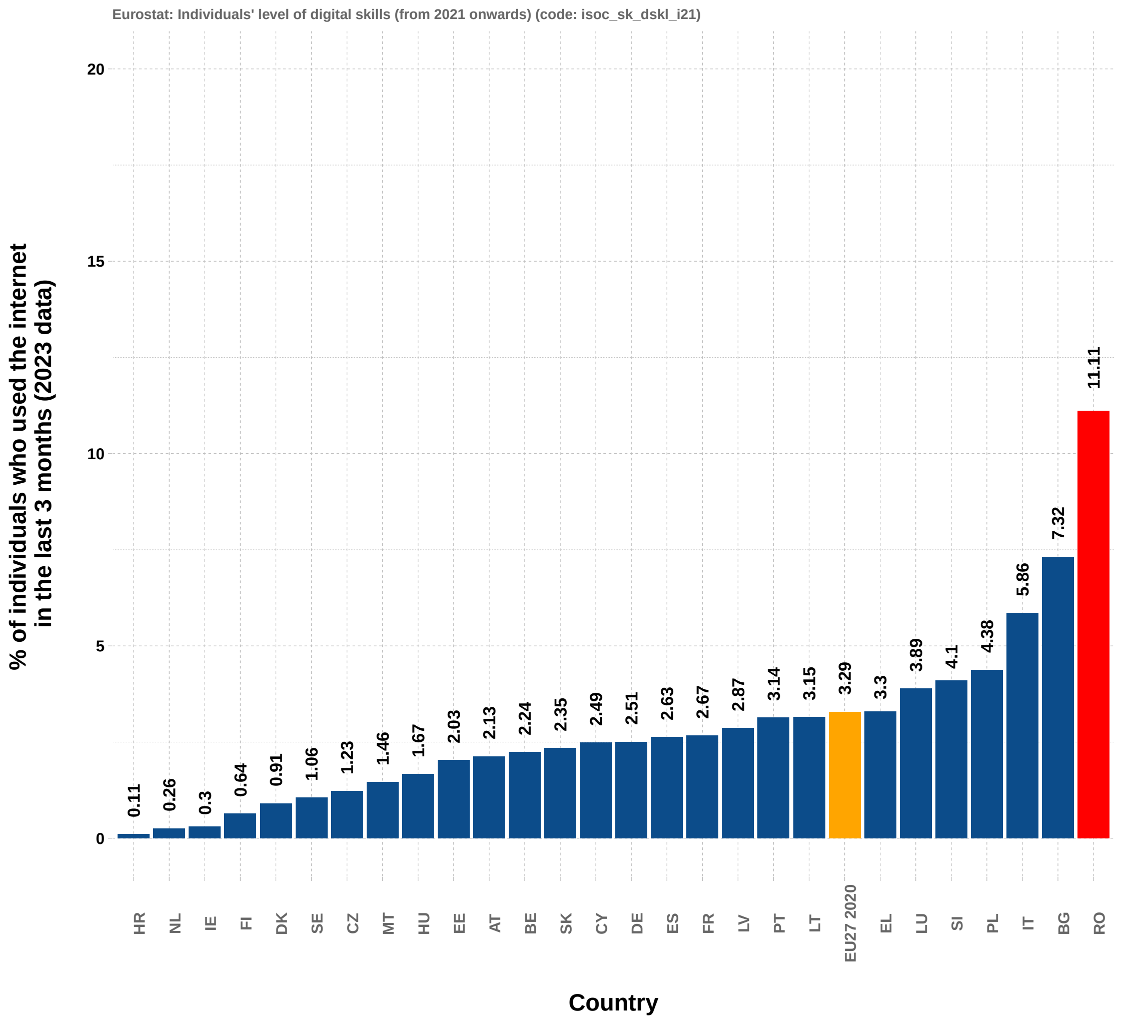

Figure S31: Individuals with no overall digital skills

6.2 Eurostat data from dataset "Individuals - internet activities"

Online data code: `isoc_ci_ac_i`

link: `https://ec.europa.eu/eurostat/databrowser/view/isoc_ci_ac_i/default/table?lang=en`

```
# Download data
eurostat_data2 <- get_eurostat("isoc_ci_ac_i",
                               filters = list(ind_type = "IND_TOTAL", unit = "PC_IND_IU3"))

# Create labels for table indicators
eu_i.labels <- data.frame(
  indic_is = c(
    "I_IUSNET",
    "I_IHIF"
  ),
  indic_is.label = c(
    "Internet use: participating in social networks (creating user profile, posting messages or other contributions to
    ↪  facebook, twitter, etc.)",
    "Internet use: seeking health information"
  )
)

# Subset data
eurostat_data2 <- eurostat_data2 %>%
  # filter data for 2023
  filter(str_sub(time, 1, 4) == "2023") %>%
  # select all types of respondents
  filter(ind_type == "IND_TOTAL") %>%
  # select respondents who used the internet in the last 3 months
  filter(unit == "PC_IND_IU3") %>%
  # filter data for countries in the EU (also keep the EU mean)
  filter(geo %in% eu_c.labels$geo | geo == "EU27_2020") %>%
  # rename EU27 label
  mutate(geo = ifelse(geo == "EU27_2020", "EU27 2020", geo)) %>%
  # select only needed columns
  dplyr::select(geo, indic_is, values) %>%
  # select only needed indicators
  filter(indic_is == "I_IUSNET" | indic_is == "I_IHIF") %>%
  # attach indicator labels
  left_join(eu_i.labels) %>% relocate(indic_is.label, .after = indic_is) %>%
  # attach country names
  left_join(eu_c.labels) %>% relocate(geo.name, .after = geo)
```

figure S32

```
#
I_IUSNET <- eurostat_data2 %>%
  filter(indic_is == "I_IUSNET")
#
I_IUSNET %>%
  ggplot() +
  aes(x = reorder(geo, values), y = values) +
  geom_col(fill = ifelse(I_IUSNET$geo == "RO", "red", ifelse(I_IUSNET$geo ==  "EU27 2020", "orange", "#0C4C8A"))) +
  labs(
    # title = "Internet use: participating in social networks (creating user profile, posting messages \nor other contributions
    ↪  to facebook, twitter, etc.)",
    subtitle = "Eurostat: Individuals - internet activities (code: isoc_ci_ac_i)",
    x = "Country",
    y = "% of individuals who used the internet \nin the last 3 months (2023 data)"
  ) + scale_y_continuous(limits=c(0, 110)) +
  ggthemes::theme_pander() +
  theme(plot.title = element_text(size = 20),
    plot.subtitle = element_text(size = 9, colour = "gray40", face = "bold"),
    axis.title.y = element_text(face = "bold", margin = margin(t = 0, r = 20, b = 0, l = 10), size = 15),
    axis.title.x = element_text(face = "bold", margin = margin(t = 20, r = 20, b = 0, l = 0), size = 15),
    axis.text.x = element_text(face = "bold", size = 10, angle = 90, colour = "gray40"),
    axis.text.y = element_text(face = "bold", size = 10)
  )  +
  geom_text(aes(label = values), size = 3.8, vjust = 0.5, nudge_x = 0, hjust = -0.5, color = "black",
              angle = 90, fontface = "bold")
```

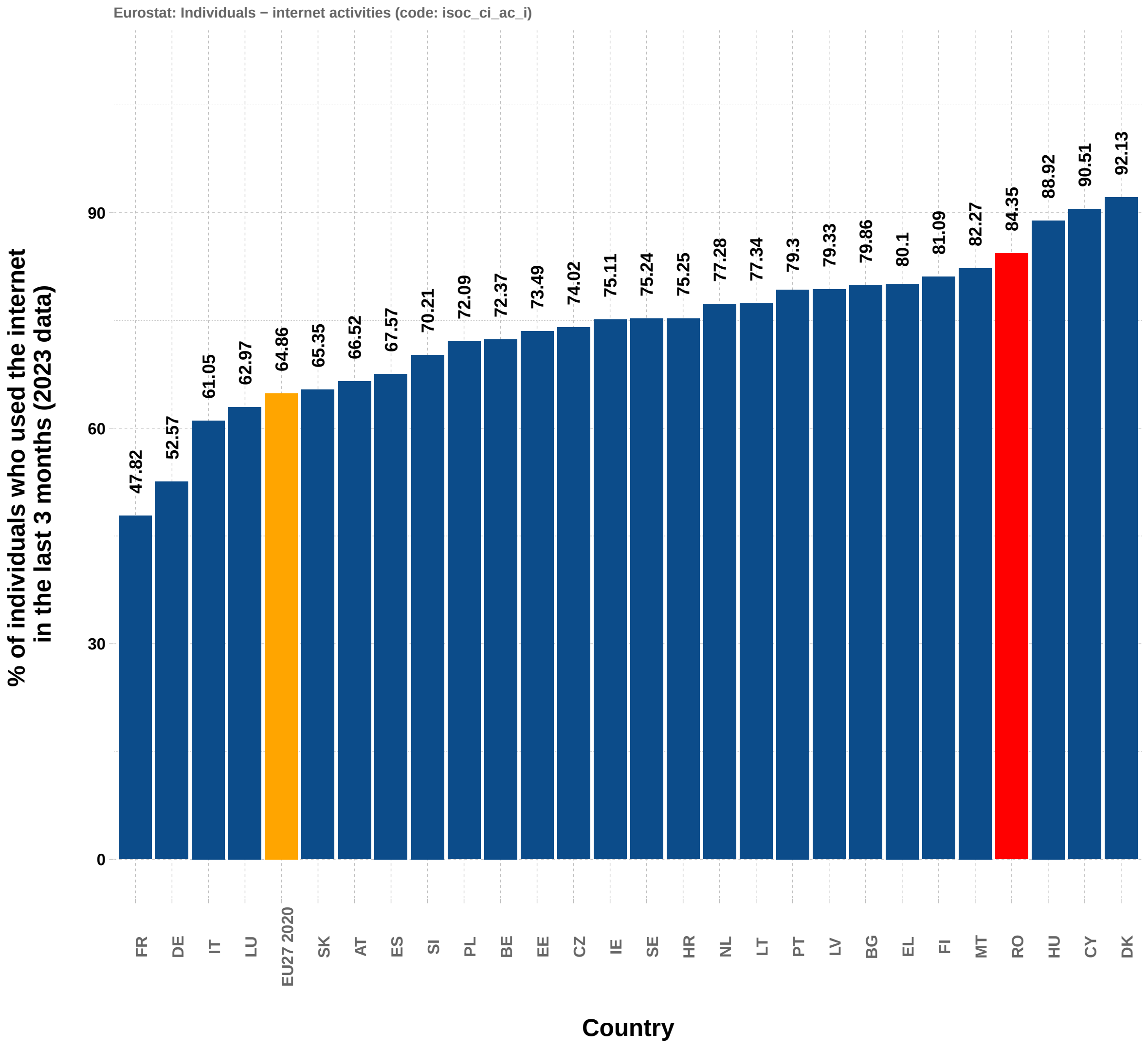

Figure S32: Internet use: participating in social networks (creating user profile, posting messages or other contributions to facebook, twitter, etc.)

figure S33

```
#
I_IHIF <- eurostat_data2 %>%
  filter(indic_is == "I_IHIF")
#
I_IHIF %>%
  ggplot() +
  aes(x = reorder(geo, values), y = values) +
  geom_col(fill = ifelse(I_IHIF$geo == "RO", "red", ifelse(I_IHIF$geo ==  "EU27 2020", "orange", "#0C4C8A"))) +
  labs(
    # title = "Internet use: seeking health information",
    subtitle = "Eurostat: Individuals - internet activities (code: isoc_ci_ac_i)",
    x = "Country",
    y = "% of individuals who used the internet \nin the last 3 months (2023 data)"
  ) + scale_y_continuous(limits=c(0, 100)) +
  ggthemes::theme_pander() +
  theme(plot.title = element_text(size = 20),
    plot.subtitle = element_text(size = 9, colour = "gray40", face = "bold"),
    axis.title.y = element_text(face = "bold", margin = margin(t = 0, r = 20, b = 0, l = 10), size = 15),
    axis.title.x = element_text(face = "bold", margin = margin(t = 20, r = 20, b = 0, l = 0), size = 15),
    axis.text.x = element_text(face = "bold", size = 10, angle = 90, colour = "gray40"),
    axis.text.y = element_text(face = "bold", size = 10)
  )  +
  geom_text(aes(label = values), size = 3.8, vjust = 0.5, nudge_x = 0, hjust = -0.5, color = "black",
              angle = 90, fontface = "bold")
```

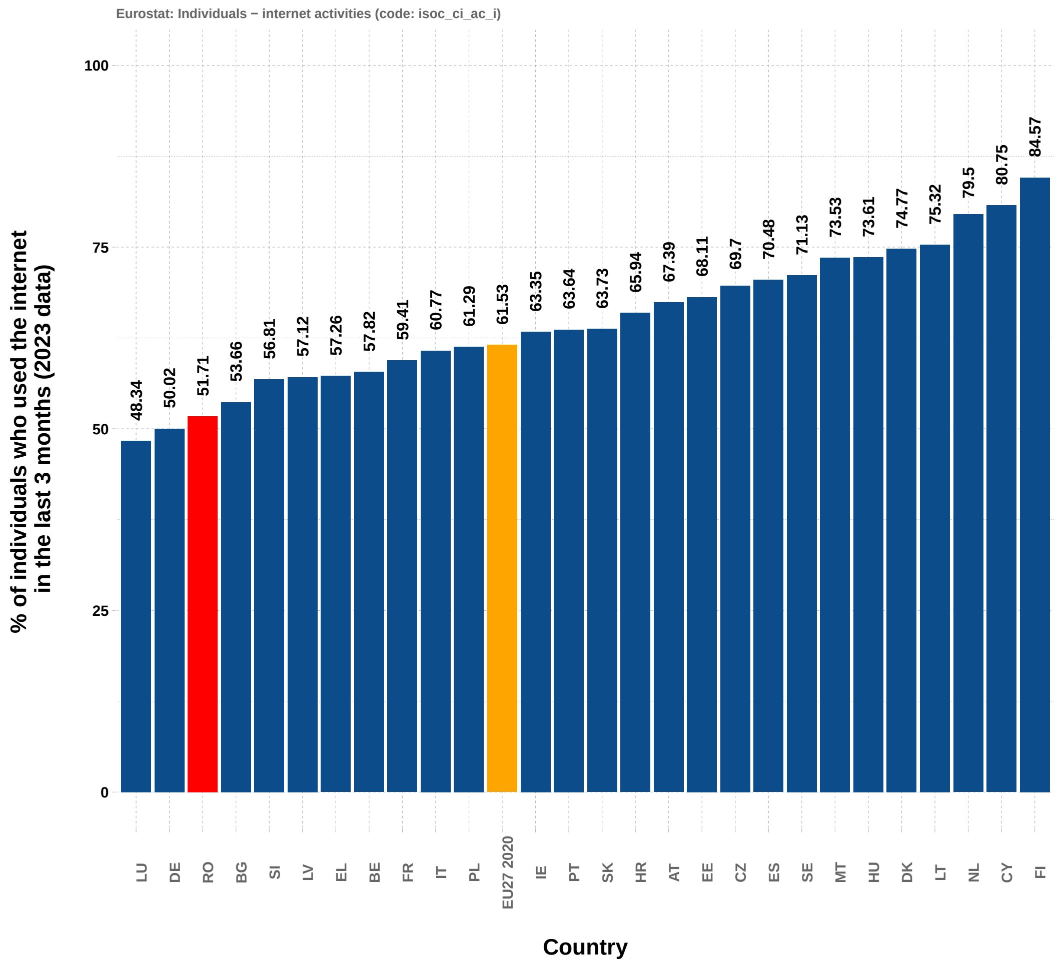

Figure S33: Internet use: seeking health information

6.3 Eurostat data from dataset "Evaluating data, information and digital content (2021 onwards)"

Online data code: isoc_sk_edic_i21

link: https://ec.europa.eu/eurostat/databrowser/view/isoc_sk_edic_i21/default/table?lang=en

```r
# Download data
eurostat_data3 <- get_eurostat("isoc_sk_edic_i21")

# Create labels for table indicators
eu_i.labels <- data.frame(
  indic_is = c(
    "I_UDI",
    "I_TIC",
    "I_TICCSFOI",
    "I_TICIDIS",
    "I_TICNIDIS",
    "I_TIC2",
    "I_TICXND",
    "I_TICXSKL",
    "I_TICXOTH"
  ),
  indic_is.label = c(
  "Individuals have seen untrue or doubtful information or content on the internet news sites or social media (3 months)",
  "Individuals have checked the truthfulness of the information or content they found on the internet news sites or social
  ↪  media (3 months)",
  "Individuals have checked the truthfulness of the information or content found on the internet by checking the sources or
  ↪  finding other information on the internet (3 months) (Method 1)",
  "Individuals have checked the truthfulness of the information or content found on the internet by following or taking part
  ↪  in discussion on internet  regarding the information (3 months) (Method 2)",
  "Individuals have checked the truthfulness of the information or content found on the internet by discussing the information
  ↪  offline with other persons or using sources not on internet (3 months) (Method 3)",
  "Individuals have checked the truthfulness of the information or content found on the internet (3 months) by using methods
  ↪  1, 2, or 3",
  "Individuals have not checked the truthfulness of the information or content found on the internet because the individuals
  ↪  already knew that information, content or source was not reliable (3 months)",
  "Individuals have not checked the truthfulness of the information or content found on the internet because the individuals
  ↪  lacked skills or knowledge (3 months)",
  "Individuals have not checked the truthfulness of the information or content found on the internet because of other reasons
  ↪  (3 months)"
  )
)

# Subset data
eurostat_data3 <- eurostat_data3 %>%
  # filter data for 2023
  filter(str_sub(TIME_PERIOD, 1, 4) == "2023") %>%
  # select all types of respondents
  filter(ind_type == "IND_TOTAL") %>%
  # select respondents who used the internet in the last 3 months
  filter(unit == "PC_IND_IU3") %>%
  # filter data for countries in the EU (also keep the EU mean)
  filter(geo %in% eu_c.labels$geo | geo == "EU27_2020") %>%
  # rename EU27 label
  mutate(geo = ifelse(geo == "EU27_2020", "EU27 2020", geo)) %>%
  # select only needed columns
  dplyr::select(geo, indic_is, values) %>%
  # attach indicator labels
  left_join(eu_i.labels) %>% relocate(indic_is.label, .after = indic_is) %>%
  # attach country names
  left_join(eu_c.labels) %>% relocate(geo.name, .after = geo)
```

figure S34

```r
#
I_UDI <- eurostat_data3 %>%
  filter(indic_is == "I_UDI")
#
I_UDI %>%
  ggplot() +
  aes(x = reorder(geo, values), y = values) +
  geom_col(fill = ifelse(I_UDI$geo == "RO", "red", ifelse(I_UDI$geo ==  "EU27 2020", "orange", "#0C4C8A"))) +
  labs(
    # title = "Individuals have seen untrue or doubtful information \nor content on the internet news sites or social media",
    subtitle = "Eurostat: Evaluating data, information and digital content (2021 onwards) (code: isoc_sk_edic_i21)",
```

```
  x = "Country",
  y = "% of individuals who used the internet \nin the last 3 months (2023 data)"
) + scale_y_continuous(limits=c(0, 100)) +
ggthemes::theme_pander() +
theme(plot.title = element_text(size = 20),
  plot.subtitle = element_text(size = 9, colour = "gray40", face = "bold"),
  axis.title.y = element_text(face = "bold", margin = margin(t = 0, r = 20, b = 0, l = 10), size = 15),
  axis.title.x = element_text(face = "bold", margin = margin(t = 20, r = 20, b = 0, l = 0), size = 15),
  axis.text.x = element_text(face = "bold", size = 10, angle = 90, colour = "gray40"),
  axis.text.y = element_text(face = "bold", size = 10)
)  +
geom_text(aes(label = values), size = 3.8,  vjust = 0.5, nudge_x = 0, hjust = -0.5, color = "black",
              angle = 90, fontface = "bold")
```

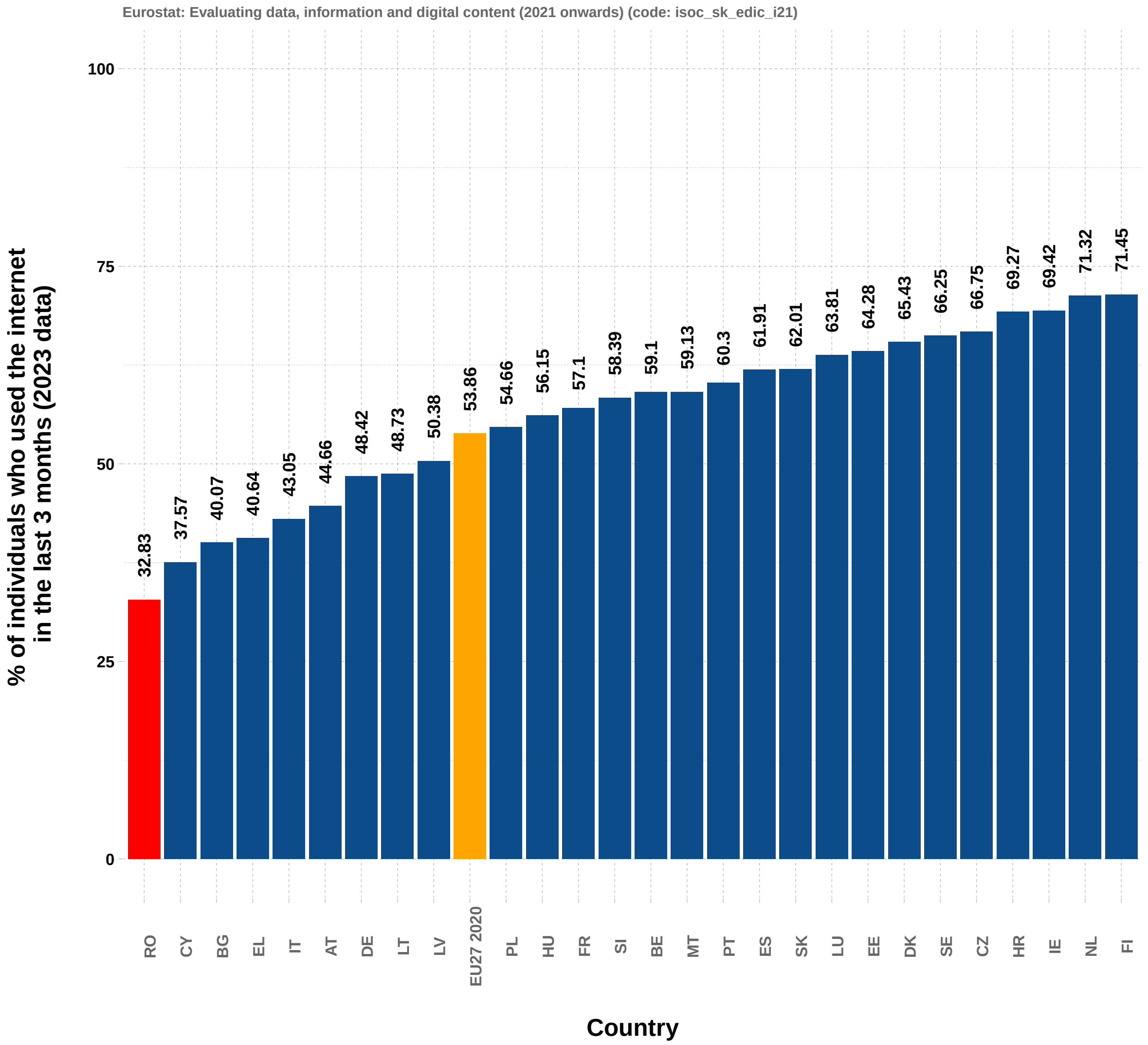

Figure S34: Individuals have seen untrue or doubtful information or content on the internet news sites or social media

figure S35

```
#
I_TIC <- eurostat_data3 %>%
  filter(indic_is == "I_TIC")
#
I_TIC %>%
  ggplot() +
  aes(x = reorder(geo, values), y = values) +
  geom_col(fill = ifelse(I_TIC$geo == "RO", "red", ifelse(I_TIC$geo ==  "EU27 2020", "orange", "#0C4C8A"))) +
  labs(
    # title = "Individuals have checked the truthfulness of the information \nor content they found on the internet news sites
    ↪  or social media",
    subtitle = "Eurostat: Evaluating data, information and digital content (2021 onwards) (code: isoc_sk_edic_i21)",
    x = "Country",
    y = "% of individuals who used the internet \nin the last 3 months (2023 data)"
  ) + scale_y_continuous(limits=c(0, 80)) +
  ggthemes::theme_pander() +
  theme(plot.title = element_text(size = 20),
    plot.subtitle = element_text(size = 9, colour = "gray40", face = "bold"),
    axis.title.y = element_text(face = "bold", margin = margin(t = 0, r = 20, b = 0, l = 10), size = 15),
    axis.title.x = element_text(face = "bold", margin = margin(t = 20, r = 20, b = 0, l = 0), size = 15),
    axis.text.x = element_text(face = "bold", size = 10, angle = 90, colour = "gray40"),
    axis.text.y = element_text(face = "bold", size = 10)
  )  +
  geom_text(aes(label = values), size = 3.8, vjust = 0.5, nudge_x = 0, hjust = -0.5, color = "black",
              angle = 90, fontface = "bold")
```

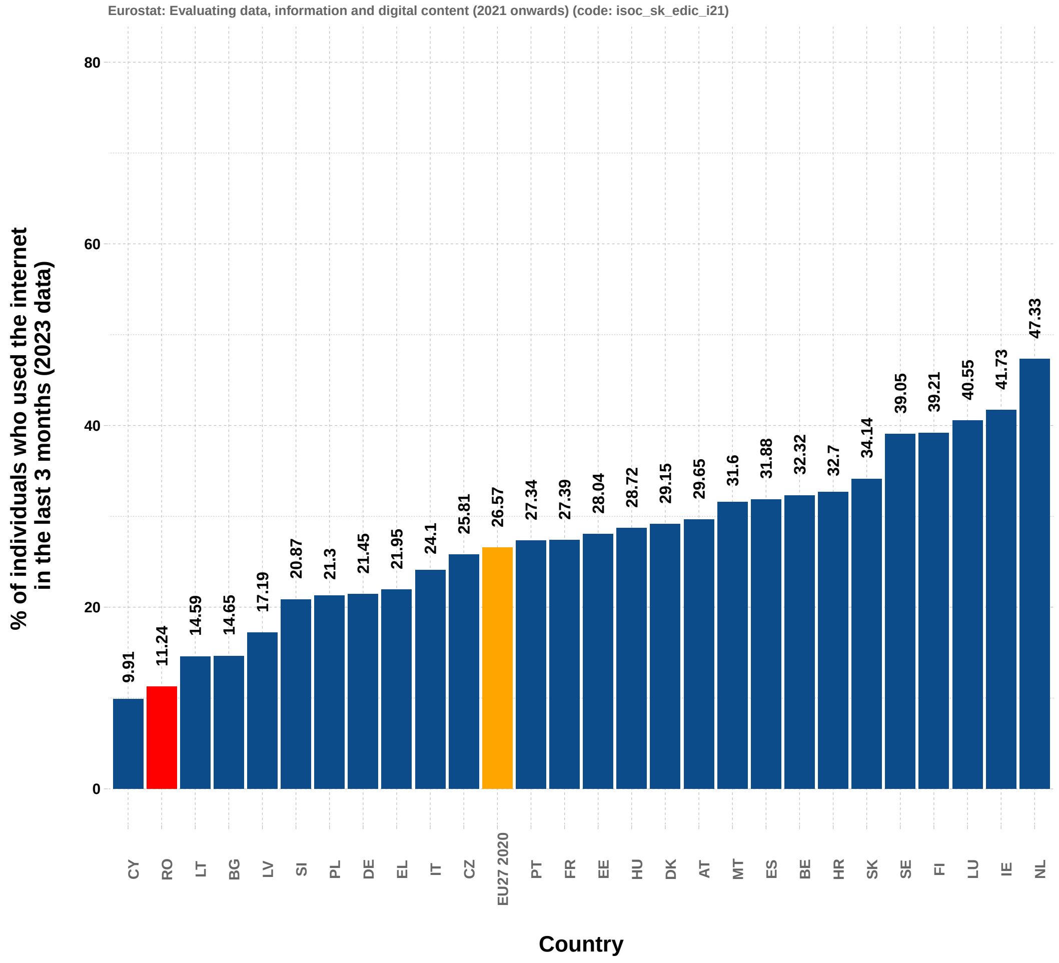

Figure S35: Individuals have checked the truthfulness of the information or content they found on the internet news sites or social media

figure S36

```
#
I_TICCSFOI <- eurostat_data3 %>%
  filter(indic_is == "I_TICCSFOI")
#
I_TICCSFOI %>%
  ggplot() +
  aes(x = reorder(geo, values), y = values) +
  geom_col(fill = ifelse(I_TICCSFOI$geo == "RO", "red", ifelse(I_TICCSFOI$geo ==  "EU27 2020", "orange", "#0C4C8A"))) +
  labs(
    # title = "Individuals have checked the truthfulness of the information \nor content found on the internet by checking the
    ↪  sources or \nfinding other information on the internet (Method 1)",
    subtitle = "Eurostat: Evaluating data, information and digital content (2021 onwards) (code: isoc_sk_edic_i21)",
    x = "Country",
    y = "% of individuals who used the internet \nin the last 3 months (2023 data)"
  ) + scale_y_continuous(limits=c(0, 80)) +
  ggthemes::theme_pander() +
  theme(plot.title = element_text(size = 20),
    plot.subtitle = element_text(size = 9, colour = "gray40", face = "bold"),
    axis.title.y = element_text(face = "bold", margin = margin(t = 0, r = 20, b = 0, l = 10), size = 15),
    axis.title.x = element_text(face = "bold", margin = margin(t = 20, r = 20, b = 0, l = 0), size = 15),
    axis.text.x = element_text(face = "bold", size = 10, angle = 90, colour = "gray40"),
    axis.text.y = element_text(face = "bold", size = 10)
  )  +
  geom_text(aes(label = values), size = 3.8, vjust = 0.5, nudge_x = 0, hjust = -0.5, color = "black",
              angle = 90, fontface = "bold")
```

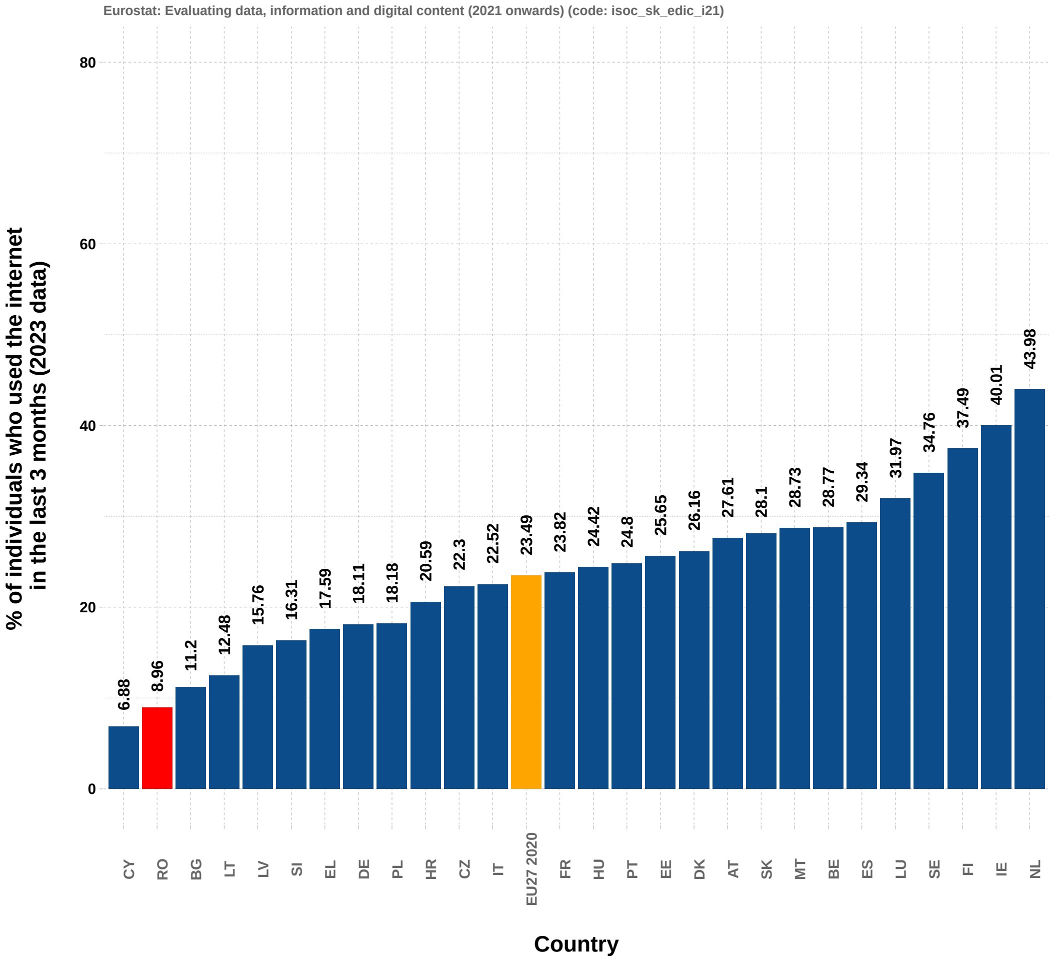

Figure S36: Individuals have checked the truthfulness of the information or content found on the internet by checking the sources or finding other information on the internet (Method 1)

figure S37

```
#
I_TICIDIS <- eurostat_data3 %>%
  filter(indic_is == "I_TICIDIS")
#
I_TICIDIS %>%
  ggplot() +
  aes(x = reorder(geo, values), y = values) +
  geom_col(fill = ifelse(I_TICIDIS$geo == "RO", "red", ifelse(I_TICIDIS$geo ==  "EU27 2020", "orange", "#0C4C8A"))) +
  labs(
    # title = "Individuals have checked the truthfulness of the information \nor content found on the internet by following or
    ↪  taking part in \ndiscussion on internet regarding the information (Method 2)",
    subtitle = "Eurostat: Evaluating data, information and digital content (2021 onwards) (code: isoc_sk_edic_i21)",
    x = "Country",
    y = "% of individuals who used the internet \nin the last 3 months (2023 data)"
  ) + scale_y_continuous(limits=c(0, 40)) +
  ggthemes::theme_pander() +
  theme(plot.title = element_text(size = 20),
    plot.subtitle = element_text(size = 9, colour = "gray40", face = "bold"),
    axis.title.y = element_text(face = "bold", margin = margin(t = 0, r = 20, b = 0, l = 10), size = 15),
    axis.title.x = element_text(face = "bold", margin = margin(t = 20, r = 20, b = 0, l = 0), size = 15),
    axis.text.x = element_text(face = "bold", size = 10, angle = 90, colour = "gray40"),
    axis.text.y = element_text(face = "bold", size = 10)
  )  +
  geom_text(aes(label = values), size = 3.8, vjust = 0.5, nudge_x = 0, hjust = -0.5, color = "black",
              angle = 90, fontface = "bold")
```

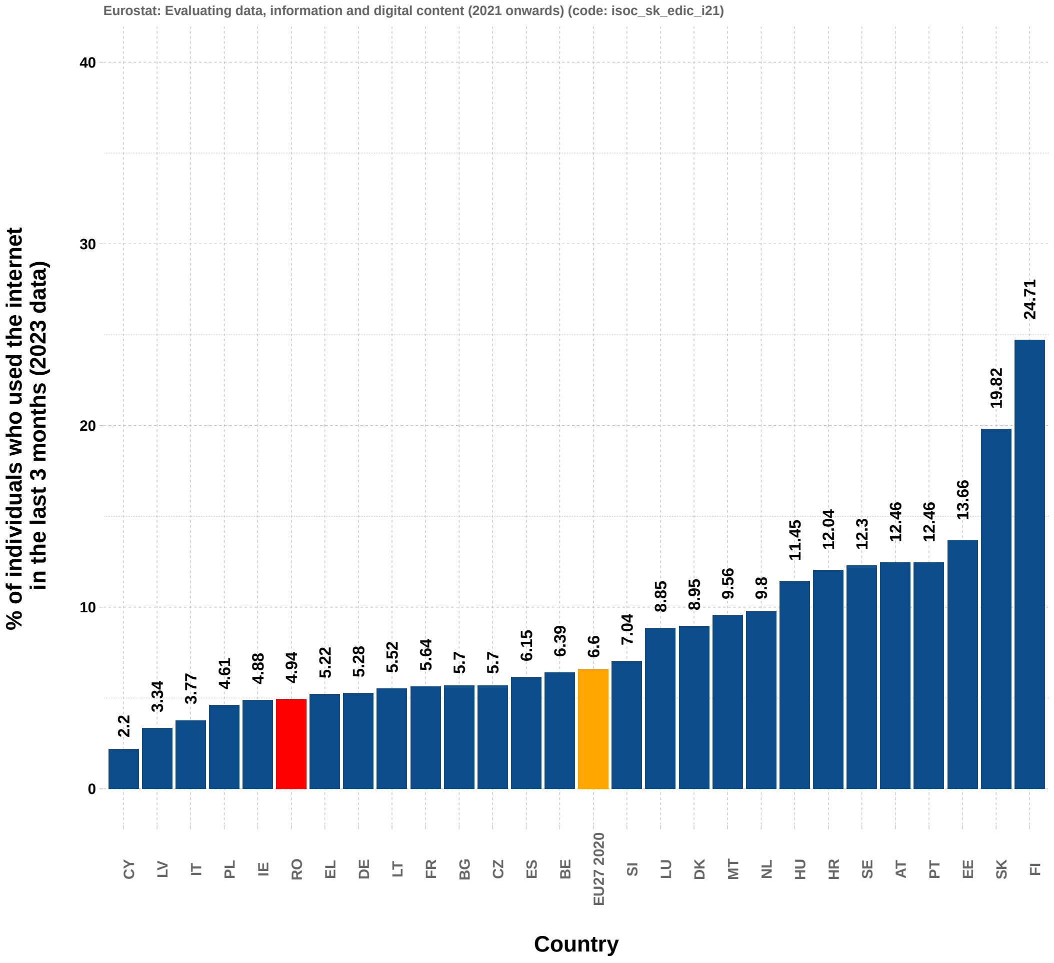

Figure S37: Individuals have checked the truthfulness of the information or content found on the internet by following or taking part in discussion on internet regarding the information (Method 2)

figure S38

```
#
I_TICNIDIS <- eurostat_data3 %>%
  filter(indic_is == "I_TICNIDIS")
#
I_TICNIDIS %>%
  ggplot() +
  aes(x = reorder(geo, values), y = values) +
  geom_col(fill = ifelse(I_TICNIDIS$geo == "RO", "red", ifelse(I_TICNIDIS$geo ==  "EU27 2020", "orange", "#0C4C8A"))) +
  labs(
    # title = "Individuals have checked the truthfulness of the information or \ncontent found on the internet by discussing
    ↪  the information offline \nwith other persons or using sources not on internet (Method 3)",
    subtitle = "Eurostat: Evaluating data, information and digital content (2021 onwards) (code: isoc_sk_edic_i21)",
    x = "Country",
    y = "% of individuals who used the internet \nin the last 3 months (2023 data)"
  ) + scale_y_continuous(limits=c(0, 40)) +
  ggthemes::theme_pander() +
  theme(plot.title = element_text(size = 20),
    plot.subtitle = element_text(size = 9, colour = "gray40", face = "bold"),
    axis.title.y = element_text(face = "bold", margin = margin(t = 0, r = 20, b = 0, l = 10), size = 15),
    axis.title.x = element_text(face = "bold", margin = margin(t = 20, r = 20, b = 0, l = 0), size = 15),
    axis.text.x = element_text(face = "bold", size = 10, angle = 90, colour = "gray40"),
    axis.text.y = element_text(face = "bold", size = 10)
  )  +
  geom_text(aes(label = values), size = 3.8, vjust = 0.5, nudge_x = 0, hjust = -0.5, color = "black",
              angle = 90, fontface = "bold")
```

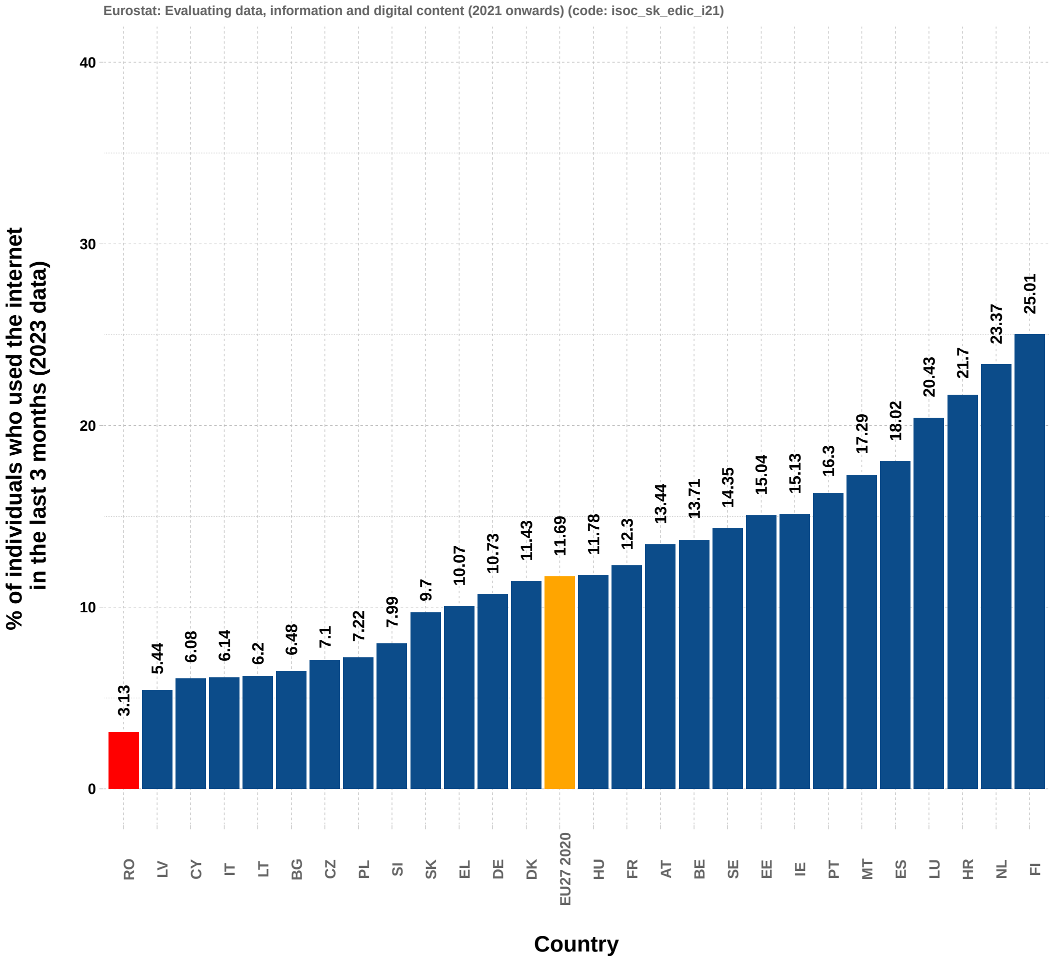

Figure S38: Individuals have checked the truthfulness of the information or content found on the internet by discussing the information offline with other persons or using sources not on internet (Method 3)

figure S39

```r
#
I_TIC2 <- eurostat_data3 %>%
  filter(indic_is == "I_TIC2")
#
I_TIC2 %>%
  ggplot() +
  aes(x = reorder(geo, values), y = values) +
  geom_col(fill = ifelse(I_TIC2$geo == "RO", "red", ifelse(I_TIC2$geo ==  "EU27 2020", "orange", "#0C4C8A"))) +
  labs(
    # title = "Individuals have checked the truthfulness of the information \nor content found on the internet by using methods
    ↪  1, 2, or 3",
    subtitle = "Eurostat: Evaluating data, information and digital content (2021 onwards) (code: isoc_sk_edic_i21)",
    x = "Country",
    y = "% of individuals who used the internet \nin the last 3 months (2023 data)"
  ) + scale_y_continuous(limits=c(0, 80)) +
  ggthemes::theme_pander() +
  theme(plot.title = element_text(size = 20),
    plot.subtitle = element_text(size = 9, colour = "gray40", face = "bold"),
    axis.title.y = element_text(face = "bold", margin = margin(t = 0, r = 20, b = 0, l = 10), size = 15),
    axis.title.x = element_text(face = "bold", margin = margin(t = 20, r = 20, b = 0, l = 0), size = 15),
    axis.text.x = element_text(face = "bold", size = 10, angle = 90, colour = "gray40"),
    axis.text.y = element_text(face = "bold", size = 10)
  )  +
  geom_text(aes(label = values), size = 3.8, vjust = 0.5, nudge_x = 0, hjust = -0.5, color = "black",
              angle = 90, fontface = "bold")
```

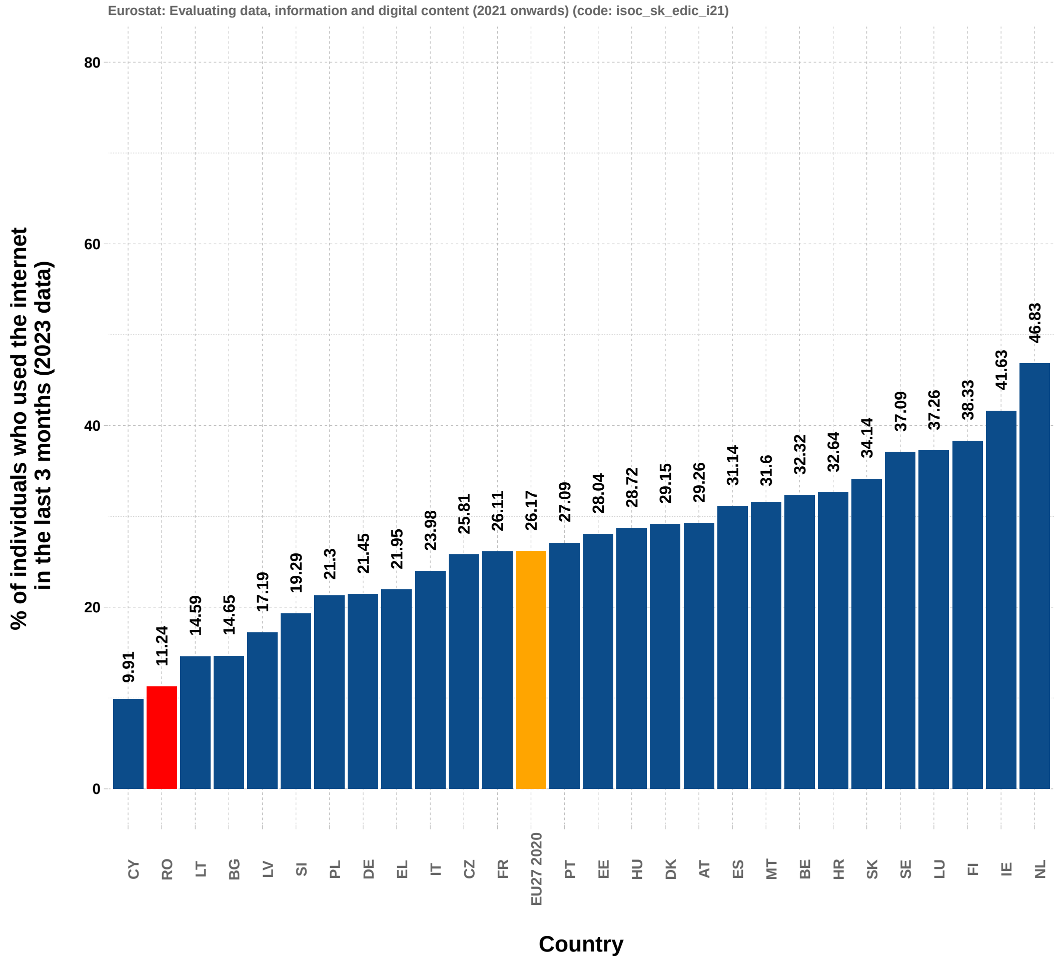

Figure S39: Individuals have checked the truthfulness of the information or content found on the internet by using methods 1, 2, or 3

figure S40

```
#
I_TICXND <- eurostat_data3 %>%
  filter(indic_is == "I_TICXND")
#
I_TICXND %>%
  ggplot() +
  aes(x = reorder(geo, values), y = values) +
  geom_col(fill = ifelse(I_TICXND$geo == "RO", "red", ifelse(I_TICXND$geo ==  "EU27 2020", "orange", "#0C4C8A"))) +
  labs(
    # title = "Individuals have not checked the truthfulness of the information \nor content found on the internet because the
↪  individuals already \nknew that information, content or source was not reliable",
    subtitle = "Eurostat: Evaluating data, information and digital content (2021 onwards) (code: isoc_sk_edic_i21)",
    x = "Country",
    y = "% of individuals who used the internet \nin the last 3 months (2023 data)"
  ) + scale_y_continuous(limits=c(0, 50)) +
  ggthemes::theme_pander() +
  theme(plot.title = element_text(size = 20),
    plot.subtitle = element_text(size = 9, colour = "gray40", face = "bold"),
    axis.title.y = element_text(face = "bold", margin = margin(t = 0, r = 20, b = 0, l = 10), size = 15),
    axis.title.x = element_text(face = "bold", margin = margin(t = 20, r = 20, b = 0, l = 0), size = 15),
    axis.text.x = element_text(face = "bold", size = 10, angle = 90, colour = "gray40"),
    axis.text.y = element_text(face = "bold", size = 10)
  )  +
  geom_text(aes(label = values), size = 3.8, vjust = 0.5, nudge_x = 0, hjust = -0.5, color = "black",
              angle = 90, fontface = "bold")
```

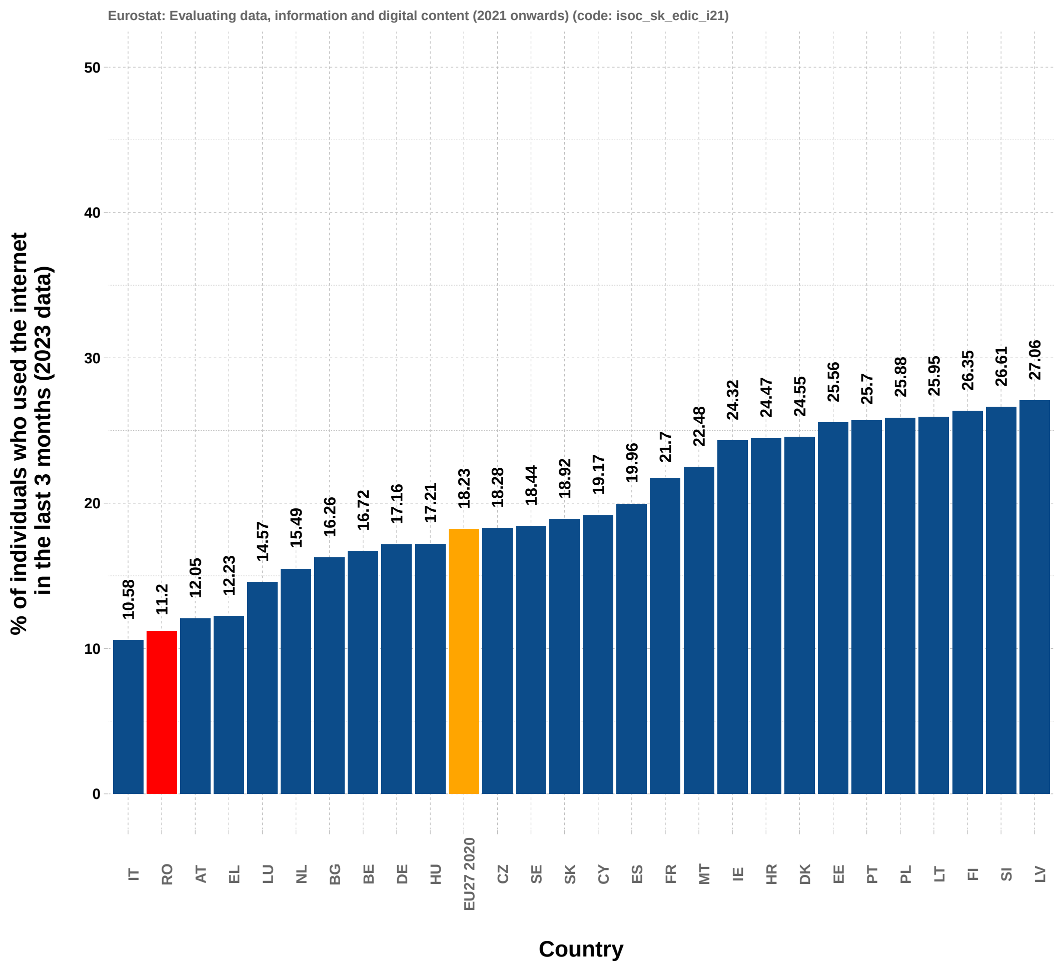

Figure S40: Individuals have not checked the truthfulness of the information or content found on the internet because the individuals already knew that information, content or source was not reliable

figure S41

```r
#
I_TICXSKL <- eurostat_data3 %>%
  filter(indic_is == "I_TICXSKL")
#
I_TICXSKL %>%
  ggplot() +
  aes(x = reorder(geo, values), y = values) +
  geom_col(fill = ifelse(I_TICXSKL$geo == "RO", "red", ifelse(I_TICXSKL$geo ==  "EU27 2020", "orange", "#0C4C8A"))) +
  labs(
    # title = "Individuals have not checked the truthfulness of the information \nor content found on the internet because the
    ↪  individuals lacked \nskills or knowledge",
    subtitle = "Eurostat: Evaluating data, information and digital content (2021 onwards) (code: isoc_sk_edic_i21)",
    x = "Country",
    y = "% of individuals who used the internet \nin the last 3 months (2023 data)"
  ) + scale_y_continuous(limits=c(0, 30)) +
  ggthemes::theme_pander() +
  theme(plot.title = element_text(size = 20),
    plot.subtitle = element_text(size = 9, colour = "gray40", face = "bold"),
    axis.title.y = element_text(face = "bold", margin = margin(t = 0, r = 20, b = 0, l = 10), size = 15),
    axis.title.x = element_text(face = "bold", margin = margin(t = 20, r = 20, b = 0, l = 0), size = 15),
    axis.text.x = element_text(face = "bold", size = 10, angle = 90, colour = "gray40"),
    axis.text.y = element_text(face = "bold", size = 10)
  )  +
  geom_text(aes(label = values), size = 3.8, vjust = 0.5, nudge_x = 0, hjust = -0.5, color = "black",
              angle = 90, fontface = "bold")
```

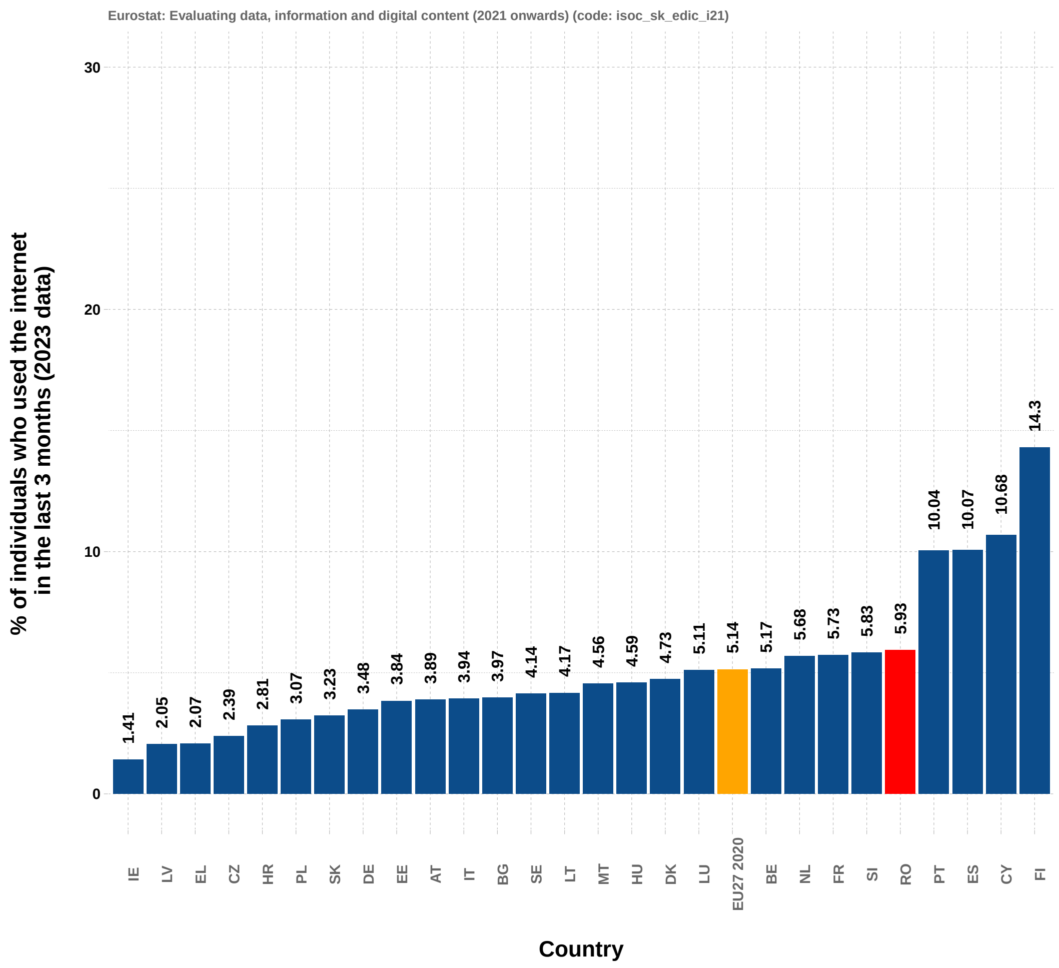

Figure S41: Individuals have not checked the truthfulness of the information or content found on the internet because the individuals lacked skills or knowledge

figure S42

```
#
I_TICXOTH <- eurostat_data3 %>%
  filter(indic_is == "I_TICXOTH")
#
I_TICXOTH %>%
  ggplot() +
  aes(x = reorder(geo, values), y = values) +
  geom_col(fill = ifelse(I_TICXOTH$geo == "RO", "red", ifelse(I_TICXOTH$geo ==  "EU27 2020", "orange", "#0C4C8A"))) +
  labs(
    # title = "Individuals have not checked the truthfulness of the information \nor content found on the internet because of
    ↪  other reasons",
    subtitle = "Eurostat: Evaluating data, information and digital content (2021 onwards) (code: isoc_sk_edic_i21)",
    x = "Country",
    y = "% of individuals who used the internet \nin the last 3 months (2023 data)"
  ) + scale_y_continuous(limits=c(0, 40)) +
  ggthemes::theme_pander() +
  theme(plot.title = element_text(size = 20),
    plot.subtitle = element_text(size = 9, colour = "gray40", face = "bold"),
    axis.title.y = element_text(face = "bold", margin = margin(t = 0, r = 20, b = 0, l = 10), size = 15),
    axis.title.x = element_text(face = "bold", margin = margin(t = 20, r = 20, b = 0, l = 0), size = 15),
    axis.text.x = element_text(face = "bold", size = 10, angle = 90, colour = "gray40"),
    axis.text.y = element_text(face = "bold", size = 10)
  )  +
  geom_text(aes(label = values), size = 3.8, vjust = 0.5, nudge_x = 0, hjust = -0.5, color = "black",
              angle = 90, fontface = "bold")
```

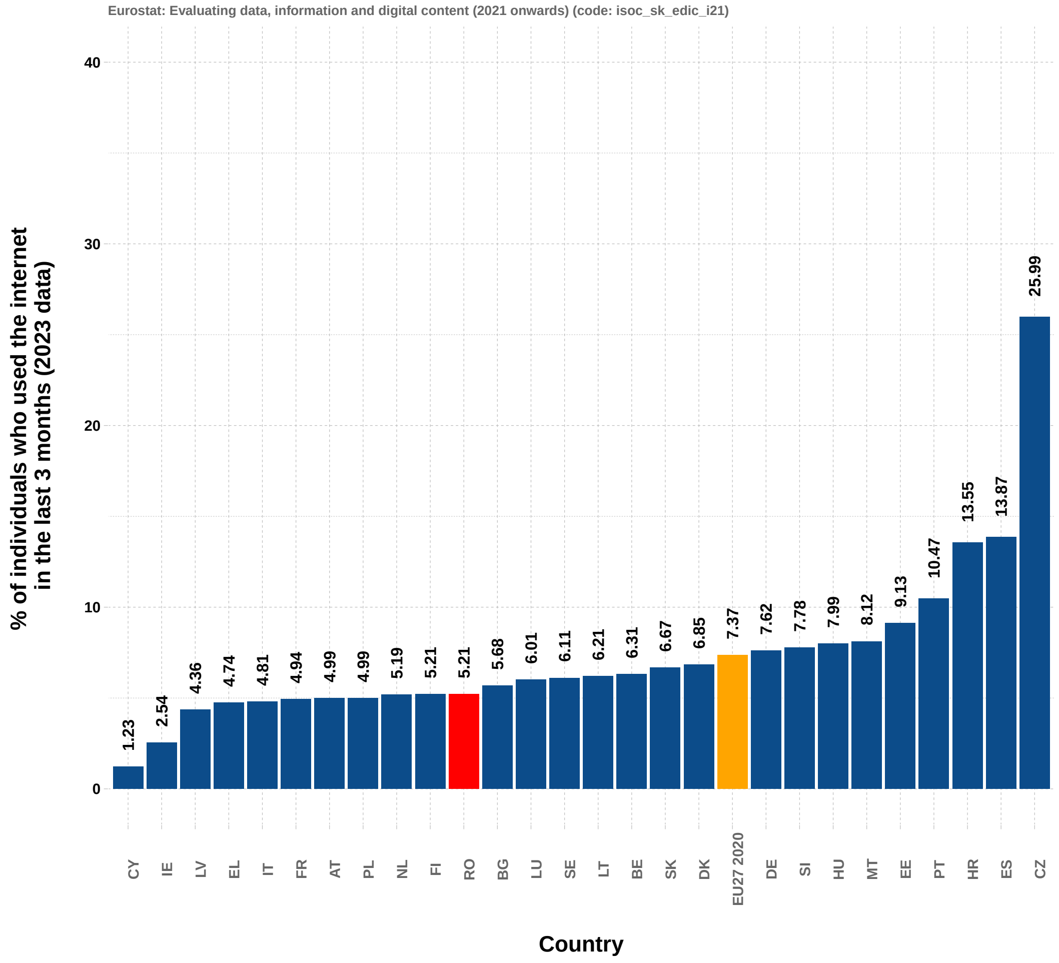

Figure S42: Individuals have not checked the truthfulness of the information or content found on the internet because of other reasons
